# Development of Acridone Derivatives: Targeting *c-MYC* Transcription in Triple-Negative Breast Cancer with Inhibitory Potential

**DOI:** 10.3390/antiox13010011

**Published:** 2023-12-20

**Authors:** Jing-Wei Liang, Zhi-Chao Gao, Lu-Lu Yang, Wei Zhang, Ming-Zhe Chen, Fan-Hao Meng

**Affiliations:** 1 School of Pharmacy, China Medical University, Shenyang 110000, China; hy0207147@hainmc.edu.cn (J.-W.L.); gzc3217@126.com (Z.-C.G.); y18272950945@163.com (L.-L.Y.); zw368241136@163.com (W.Z.);; 2 School of Pharmacy, Hainan Medical University, Haikou 570100, China; 3 Department of Medical Oncology, Cancer Hospital of China Medical University, Shenyang 110044, China

**Keywords:** acridone derivatives, *c-MYC*, G-quadruplex, TNBC, ROS

## Abstract

Breast cancer, especially the aggressive triple-negative subtype, poses a serious health threat to women. Unfortunately, effective targets are lacking, leading to a grim prognosis. Research highlights the crucial role of *c-MYC* overexpression in this form of cancer. Current inhibitors targeting *c-MYC* focus on stabilizing its G-quadruplex (G4) structure in the promoter region. They can inhibit the expression of *c-MYC*, which is highly expressed in triple-negative breast cancer (TNBC), and then regulate the apoptosis of breast cancer cells induced by intracellular ROS. However, the clinical prospects for the application of such inhibitors are not promising. In this research, we designed and synthesized 29 acridone derivatives. These compounds were assessed for their impact on intracellular ROS levels and cell activity, followed by comprehensive QSAR analysis and molecular docking. Compound N**8** stood out, significantly increasing ROS levels and demonstrating potent anti-tumor activity in the TNBC cell line, with excellent selectivity shown in the docking results. This study suggests that acridone derivatives could stabilize the *c-MYC* G4 structure. Among these compounds, the small molecule N**8** shows promising effects and deserves further investigation.

## 1. Introduction

### 1.1. Epidemiology and Character of Triple-Negative Breast Cancer

According to a report from the World Cancer Research Fund International, in 2020, breast cancer surpassed lung cancer as the most common cancer worldwide. Approximately 2.3 million new cases of breast cancer are diagnosed annually, accounting for about 11.7% of all new cancer cases. Breast cancer was associated with approximately 6.9% of cancer-related deaths [1]. In 2013, the International St. Gallen Breast Cancer Conference introduced an important molecular classification system for breast cancer based on immunohistochemistry and molecular biology characteristics. This system categorizes breast cancer into several main molecular subtypes: Luminal A, Luminal B (HER2 positive), Luminal B (HER2 negative), HER2 positive, and triple negative [2]. Triple-negative breast cancer (TNBC) is a subtype that does not express ER, PR, or HER2. Compared to other breast cancer subtypes, TNBC tends to be more aggressive, characterized by a high degree of invasiveness and a propensity to spread to surrounding tissues and lymph nodes. It also carries a higher risk of recurrence [3]. Given the absence of hormone receptors and HER2 expression, TNBC typically does not derive benefit from hormone therapy or targeted therapeutics such as Herceptin [4]. Therefore, standard treatment approaches often involve chemotherapy and radiation therapy, which can impose a significant physical burden [5]. TNBC is currently a focal point of breast cancer research, with scientists actively seeking more effective treatment strategies, including novel therapies targeting specific molecular markers, to improve patient outcomes.

### 1.2. Role of c-MYC in Biology and G-Quadruplex Structure of c-MYC

The *c-MYC* oncoprotein, recognized for its role as the primary orchestrator of gene expression [6], stands as a versatile transcription factor with intricate involvement in the intricate control of numerous physiological processes. The *c-MYC* gene exhibits overexpression in a staggering 70% of human cancers, including TNBC [7]. Consequently, the downregulation of *c-MYC* has emerged as an enticing strategy for cancer treatment.

**Figure 1:**
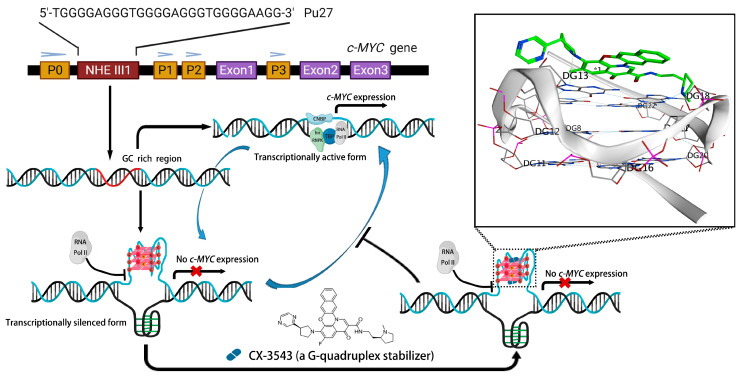
G-quadruplex stabilizer mediates transcription of *c-MYC* gene (CNBP: cellular nucleic acid binding protein, hnRNPK: heterogeneous nuclear ribonucleoprotein K, TBP: TATA-binding protein, RNA Pol II: RNA polymerase II). The black box displays the conformation of the interaction between CX-**3543** (green molecule marked as * 1) and the G4 structure of *c-MYC* (gray white ribbon composed of white molecules).

Within the genetic architecture of the *c-MYC* gene lies a crucial element known as the nuclease hypersensitivity element III1 (NHE III1), which is located upstream of the P1 promoter, responsible for approximately 90% of the gene’s transcriptional activation Notably, the purine-rich strand of DNA in this region can assume a distinctive secondary structure known as the G-quadruplex (G4) (Figure 1). G4 structures are formed from single-stranded guanine (G)-rich sequences and can be considered four-stranded DNA secondary structures. In the presence of monovalent cations, particularly potassium ions (K+) and sodium ions (Na+), four guanines in the same strand can form a G-tetrad structure by the Hoogsteen hydrogen bond. Three G-tetrads further form the G4 structure of *c-MYC* [8]. The G4 structure represents a transient structural entity. A stabilized G4 structure holds the potential to impede the binding of RNA polymerase to NHE III1, consequently curtailing the expression of the *c-MYC* gene [9]. Moreover, the 3′ and 5′ flanking regions of G4 in *c-MYC* contribute to a capping structure that envelops the corresponding terminal tetrads, creating an attractive binding pocket for small molecule ligands [10]. Consequently, the pursuit of small molecule compounds capable of stabilizing the G4 structure of *c-MYC* emerges as a promising avenue for significantly reducing *c-MYC* protein expression, thereby exerting inhibitory effects on the growth and proliferation of TNBC cells.

### 1.3. Challenges Encountered in the Development of c-MYC G4 Stabilizer

In recent years, hundreds of small molecules stabilizing the *c-MYC* G4 structure have been reported [11,12,13,14,15]. However, to the best of our knowledge, none have gained FDA approval. This is attributed in part to drug activity and, on the other hand, to the selectivity of the drugs [6,12]. G4 structures are widely present throughout the human genome, with approximately 700,000 genes having the potential to form G4 structures [16]. This presents a significant challenge in the development of *c-MYC* G4 stabilizers. Fortunately, experimental evidence has confirmed that the binding affinity of the same compound to G4 structures of different genes varies [11,13,17]. Therefore, the development of a drug with high selectivity for *c-MYC* G4 structure and robust anti-tumor activity holds promise as a reliable choice for TNBC therapy, bearing crucial significance in improving the prognosis of TNBC patients [16].

In recent years, hundreds of small molecules stabilizing the G4 structures of *c-MYC* have been reported [11,12,13,14,15]. However, to the best of our knowledge, none have gained FDA approval. This is attributed in part to drug activity and, on the other hand, to the selectivity of the drugs [6,12]. G4 structures are widely present throughout the human genome, with approximately 700,000 genes having the potential to form G4 structures [16]. This presents a significant challenge in the development of *c-MYC* G4 stabilizers. Fortunately, experimental evidence has confirmed that the binding affinity of the same compound to G4 structures of different genes varies [11,13,17]. Therefore, the development of a drug with high selectivity for *c-MYC* G4 structure and robust anti-tumor activity holds promise as a reliable choice for TNBC therapy, bearing crucial significance in improving the prognosis of TNBC patients [12].

### 1.4. Characteristics of Acridone Derivatives and Their Potential Opponent in G4 Stabilizing

Acridone alkaloids such as aconycine [18] are derived from rutaceae and possess three planar rings, enabling their insertion into base pairs of double-stranded DNA—a crucial feature in the development of effective anticancer chemotherapy [19,20,21,22]. Given the structural similarity between the G4 of *c-MYC* and the DNA double helix, both consisting of stacked structures formed by base pairs, recent studies have reported the stabilizing effect of acridone derivatives on the G4 structure of *c-MYC* [23,24]; this further underscores the potential of acridone derivatives as a core scaffold for *c-MYC* G4 stabilizers. Building upon this foundation, the present study employs fragment-based growth techniques to design and synthesize novel acridone derivatives, thereby enhancing the structural diversity of molecules acting as G4 stabilizers. Subsequently, these compounds underwent further screening to identify those capable of inducing ROS production and promoting apoptosis in tumor cells.

## 2. Materials and Methods

### 2.1. Fragment-Based Drug Design

We employed the fragment module of MOE software (MOE 2019.0102) for drug design. Initially, the parent nucleus was subjected to constrained docking to identify conformations capable of forming a π-π stacking interaction with the guanine. Subsequently, this conformation was used for fragment growing: the N atom of the acridone was designated as the growth point, and unoccupied cavities within the active site were marked as the target for fragment growth. Pharmacophore features were constructed based on the hydrophobic/hydrophilic properties of the atoms comprising these cavities. Finally, hit compounds were obtained through screening the linker database in MOE.

### 2.2. General Procedures

^1^H-NMR and ^13^C-NMR spectra were recorded on a Varian NMR spectrometer operating at 600 MHz for ^1^H and 151 MHz for ^13^C. All chemical shifts were measured in DMSO-*d6* as solvents. All chemicals were purchased from Sinoreagent Chemical Reagent (Beijing, China) and were used as received unless stated otherwise. Analytical TLC was performed on Haiyang (Qingdao Haiyang Chemical Co., Ltd., Qingdao, China) silica gel 60 F254 plates and visualized by UV and potassium permanganate staining. Flash column chromatography was performed on Haiyang (Qingdao Haiyang Chemical Co., Ltd.) gel 60 (40–63 mm).

### 2.3. Synthesis

Ethyl azidoacetate (3) was prepared from ethyl acetate and sodium azide according to the reported method [25]. The reaction of azides 4a–k involves a diazotization reaction, which was prepared according to the reported method [26]. To a solution of ethyl 2-azidoacetate (3, 1 mmol) or aryl azide derivative (4a–4k, 1 mmol) and 10-(prop-2-yn-1-yl)acridin-9(10H)-one (2) (1 mmol) in EtOH/H_2_O (3:1, 10 mL) at room temperature, copper (II) sulfate pentahydrate (0.1 mmol) and sodium ascorbate (20 mg) were added. The reaction mixture was stirred at room temperature for 2 h until the starting material disappeared, as indicated by TLC. Then, the mixture was diluted with water (10 mL) and filtered to give a crude product, and the crude product was purified by column chromatography to afford the corresponding L series compounds (70–80%). The NMR data of these compounds and the additional synthesis steps required for L**6**–L**9** were included in the Supplementary Materials (Scheme S1, Figures S1–S58).

The benzoyl amide derivative (1 mmol) was dissolved in toluene (15 mL), and 1,3-dichloropropanone was added, followed by a reflux reaction for 4 h. After removing a small amount of solvent by vacuum evaporation, the mixture was allowed to stand for precipitation. Upon filtration, the intermediates 6a–6h were obtained. The acridone (7) was dissolved in DMF, and NaH (1.2 mmol) was added slowly until no more bubbles were produced. Then, 6a–6h were added, and the mixture was stirred at 60 °C for 1 h. The reaction mixture was poured into water, and filtration yielded off-white solid N**1**–N**8** (30–50%).

### 2.4. Cell Lines and Cell Culture

The human breast cancer MDA-MB-231 cell line was purchased from ATCC (ATCC; Rockville, MD, USA). MDA-MB-231 cells were cultured in DMEM High-Glucose medium (Beyotime, Nanjing, China) containing 10% fetal bovine serum (FBS, Hyclone, UT, USA) and antibiotics (100 IU/mL penicillin and 100 μg/mL streptomycin, Beyotime, Nanjing, China) at 37 °C in a humidified 5% CO_2_ atmosphere. The cells were routinely cultured in cells Petri dishes (Beyotime, Nanjing, China) or were seeded in 96-well plates (Beyotime, Nanjing, China).

### 2.5. ROS Detection Assay

The detection of intracellular ROS levels was performed by a 2′-7′dichlorofluorescin diacetate (DCFH-DA) kit (S0033S; Beyotime, Nanjing, China). Cells were plated in 100 µL of culture medium at a density of 5000 cells per well in 96-well plates with transparent bottoms and black walls (FCP965; Beyotime, Nanjing, China), with 3 replicate wells in each experimental group. The plates were then incubated for 24 h. Subsequently, the compounds were individually dissolved by PBS to 10 mM and then introduced into each well at concentrations of 0.1 µM, 1 µM, 10 µM, 100 µM, and 1000 µM. The plates were further incubated at 37 °C for 1 h. Next, cells were washed with PBS twice and then incubated with DCFH-DA at a concentration of 10 µM. Following a 30 min incubation in darkness, fluorescence measurements were obtained using a multi-function microplate reader (INFINITE E PLEX, Tecan, Männedorf, Switzerland). The EC_50_ values were calculated using GraphPad Prism 8. ROS levels were further assessed utilizing inverted fluorescence microscopy (DMi8; Leica, Wetzlar, Germany).

### 2.6. Cell Viability Assay

We evaluated cell viability using MTT assay (ST316; Beyotime, Nanjing, China). MDA-MB-231 cells were evenly distributed at a density of 5000 cells per well in triplicate within 96-well plates. Following a 24 h incubation period, the cells were subjected to various concentrations of the compounds (0.1, 1, 10, 100, 1000 μM), which were initially dissolved by PBS to 10 mM. The positive control group received Quarfloxin (CX-**3543**) (A12380, Adooq Bioscience, Nanjing, China). The negative control group received an equivalent volume of DMEM. After an additional 48 h incubation, 5% MTT solution was added to each well (20 μm of MTT and 100 μL of medium) and incubated at 37 °C for 4 h. The optical density (OD) was measured using a multi-function microplate reader (INFINITE E PLEX, Tecan, Männedorf, Switzerland) at an absorbance wavelength of 490 nm. The cell viabilities were calculated using GraphPad Prism 8 software as follows: (OD experiment − OD background)/(OD negative − OD background) × 100%.

### 2.7. Quantitative Real-Time PCR

MDA-MB-231 cells were seeded at a density of 4 × 10^5^ cells per well in a 60 mm dish and allowed to adhere for 24 h. Subsequently, the cells underwent treatment with varying concentrations of N-**8** (ranging from 0 to 10 μM) or CX3543 at a fixed concentration of 10 μM for a duration of 24 h. Following the treatments, total RNA extraction was carried out using TRIzol reagent (R0016; Beyotime, Nanjing, China), and complementary DNA (cDNA) was synthesized using the PrimeScript II 1st strand cDNA synthesis kit (6210A; TAKARA Bio, Beijing, China). Quantitative real-time polymerase chain reaction (QRT-PCR) analysis was conducted with the Quanti-Tect SYBR Green PCR kit (RR820A; TAKARA Bio, Beijing, China) on a Roche Light Cycler 480 II sequence detection system (Roche, Basel, Switzerland). The investigation focused on determining the expression levels of *c-MYC* in TNBC cells. Data analyses were performed employing the cycle threshold (Ct) method and the 2^−ΔΔCt^ formula. Primer sequences were synthesized by Synbio Tech (Suzhou, China).

PCR primary pairs sequences:

*c-MYC*: forward primer (FP), 5-GGACGACGAGACCTTCATCAA-3; and reverse primer (RP) 5-CCAGCTTCTCTGAGACGAGCTT-3.

β-actin: forward primer (FP), 5-GCATCCTGTCGGCAATGC-3; and reverse primer (RP) 5-GTTGCTATCCAGGCTGTGC-3.

The qPCR thermal cycling conditions were as follows: initial denaturation at 95 °C for 30 s, followed by 40 amplification cycles consisting of denaturation at 95 °C for 15 s, annealing at 62 °C for 1 min, and extension at 40 °C for 30 s.

### 2.8. Western Blotting Assay

MDA-MB-231 cells were cultured in 6-well plates (1 × 10^6^ cells per well) and incubated with different concentrations of N-**8** (0, 2.5, 5, and 10 μM) or 10 μM CX-**3543** for 24 h. Protein extraction was performed using the protein extraction kit (78835; Thermo Fisher Scientific, Inc., Waltham, MA, USA), and the protein concentrations were determined using the BCA protein assay kit (23225; Thermo Fisher Scientific, Inc.). Subsequently, cytoplasmic protein extracts were reconstituted in a loading buffer and boiled for 5 min. Proteins (20–50 μg per sample) were separated by electrophoresis on 8–12% SDS-PAGE and then transferred onto polyvinylidene difluoride membranes. The membranes were incubated in a 5% nonfat milk solution at room temperature for 1 h, followed by an overnight incubation at 4 °C on a shaker with specific primary antibodies. Subsequently, the membranes were incubated with secondary antibodies for 2 h at room temperature. Immunoblots were developed using chemiluminescence and detected using the ImageQuant Analyzer (ImageQuant LAS 4000, GE Healthcare, Phoenix, AZ, USA). The Western blot assay employed primary antibodies targeting β-actin (AF0003; Beyotime, Nanjing, China), *c-MYC* (ab32072; Abcam, Cambridge, UK), and SOD2 (1ab68155; Abcam, Cambridge, UK). Secondary antibodies utilized in the assay were HRP-conjugated anti-mouse (A0216; Beyotime, Nanjing, China) and anti-rabbit (A0216; Beyotime, Nanjing, China).

### 2.9. Molecular Docking Study

The Genetic Optimization of Ligand Docking (GOLD) module in MOE (version 2019.0102) software was used to perform the molecular docking study and analyze the interaction between the ligand and receptor. The DNA-G4 structures were corrected, protonated, and stage minimized by QuickPrep function in the MOE panel: the Protonate3D was set to on, and the water farther than 4.5 Å from Ligand or receptor was deleted. The binding pocket was defined by the site finder module. The selectivity score is calculated according to the following formula:

$$\mathrm{Selective}\;\mathrm{score}(i)=10^{DockingScore|i-cMYC|}$$

In the formula, *i* is the target with a G4 structure other than *c-MYC*, and this formula is based on the calculation method of docking scoring of MOE software (version 2019.0102, Chemical ComputingGroup ULC, Montreal, QC, Canada). The heat map was drawn using the online website: http://www.heatmapper.ca (accessed on 16 October 2023) [27].

### 2.10. Statistical Analysis

All data were expressed as the mean ± standard deviation (SD) based on a minimum of three independent experiments. Statistical analysis was conducted using ANOVA followed by Dunnett’s test to assess the significance of differences. A *p*-value less than 0.05 was regarded as statistically significant. Statistical computations were carried out utilizing GraphPad Prism 8 software.

## 3. Results

### 3.1. Fragment-Based Drug Design

To design selective *c-MYC* stabilizers, the conformation of the MYC G-quadruplex bound with two small molecules was selected for further investigation (PDBid: 5W77 [28]). The small molecules lay flat on a platform formed by four guanine bases (DG7, DG11, DG16, and DG20). Due to the presence of guanines, the center of this platform constitutes a hydrophilic region, while the periphery forms a hydrophobic region. The trichloromethyl benzene moiety of the small molecule perfectly extends into this region. However, the benzofuran of the small molecule fails to establish reliable hydrogen bonds with this platform despite its structural resemblance to guanine. Inspired by the binding properties of small molecules to DNA, the insertion of a small molecule between the bases, forming π-π stacking, enhances the binding stability. Compared to benzofuran, a larger and more rigid skeleton appears to be more suitable, provided it spans across adjacent bases and engages in π-π stacking interactions with them.

**Figure 2:**
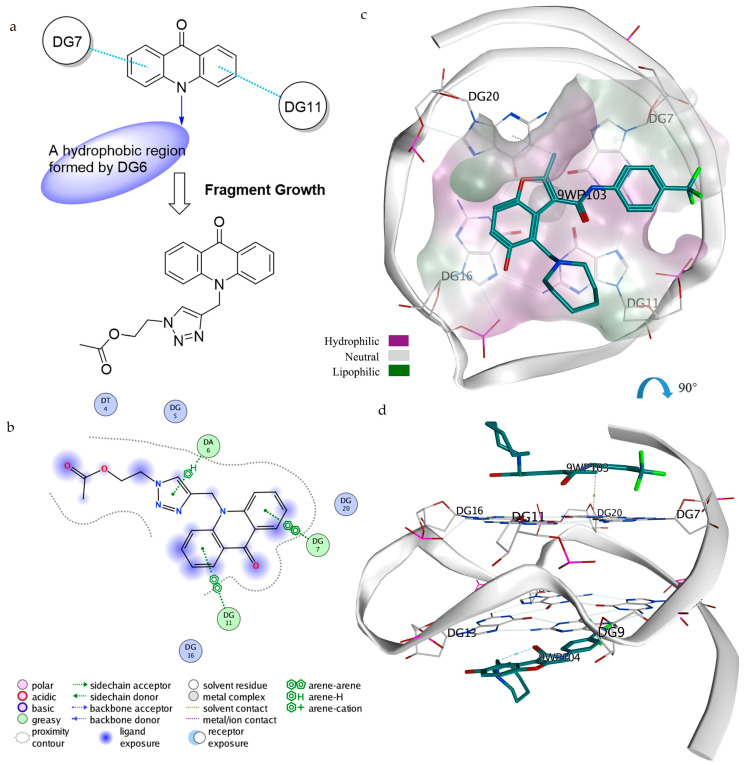
(**a**) Fragment growing-based virtual screening; (**b**) the hit compound docked with G4 structure (the dotted line represents the pocket of G4 structure); (**c**) the hydrophobicity of the platform formed by four guanine bases (Gray white molecules and ribbons: G4 structure of *c-MYC*; cyan molecule: the ligand of 5W77); (**d**) horizontal view of the platform.

In recent years, there have been a series of reports indicating that acridone derivatives exhibit a stabilizing effect on the G-quadruplex (G4) structure of *c-MYC*. Notably, acridone derivatives precisely meet the requirements outlined in the structural analysis of the skeleton [23,24]. We initiated this study by employing acridone as a ligand for docking into the G4 structure of *c-MYC*. The results demonstrated a perfect alignment with our expectations, forming two pivotal pp interactions with DG7 and DG11. Subsequently, we identified pharmacophores in the hydrophobic region of the receptor platform as targets for fragment growth (Figure 2). It is worth noting that we observed successful molecular generation exclusively in regions formed by DG6, while the other regions were hindered by spatial constraints.

### 3.2. Chemical Synthesis

**Scheme 1:**
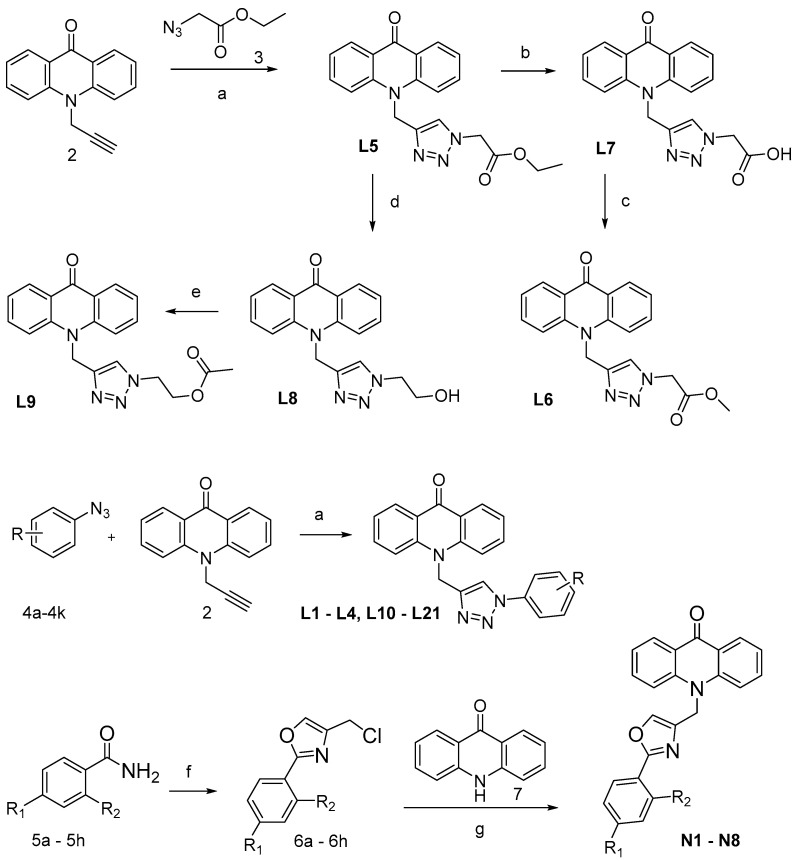
Synthesis of target compounds. Reagents and conditions: (**a**) CuSO_4_·5H_2_O, sodium ascorbate, MW, 5 min; (**b**) NaOH, EtOH/H_2_O, 80 °C, 1 h; (**c**) (Me)_2_SO_4_, MeCN, reflux, 0.5 h; (**d**) NaBH_4_, MeOH, r.t., 1 h; (**e**) Ac_2_O, THF, reflux, 1 h; (**f**) 1,3-dichloroacetone, toluene, 130 °C, reflux, 4 h; (**g**) NaH, DMF, 60 °C, 1 h.

The synthetic route was divided into two due to the structural characteristics of the target compound (Scheme 1). For compounds in the L series, alkynylation on the 10-*N* atom of picolinic ketone was achieved using bromopropyne, resulting in *N*-propargyl picolinic ketone. Subsequently, a copper-catalyzed click chemistry reaction with diazonium derivatives yields the corresponding target compounds. The synthesis of diazonium derivatives involves alkylation and diazotization reactions. For compounds in the N series, derivatives of benzoyl amide were subjected to cyclization with 1,3-dichloropropanone, producing intermediate **6a**–**6h**, which is then alkynylated with acridone to obtain the respective N**1**–N**8** compounds.

### 3.3. Acridone Derivatives Introduce ROS Production in MDA-MB-231 Cell Line

**Figure 3:**
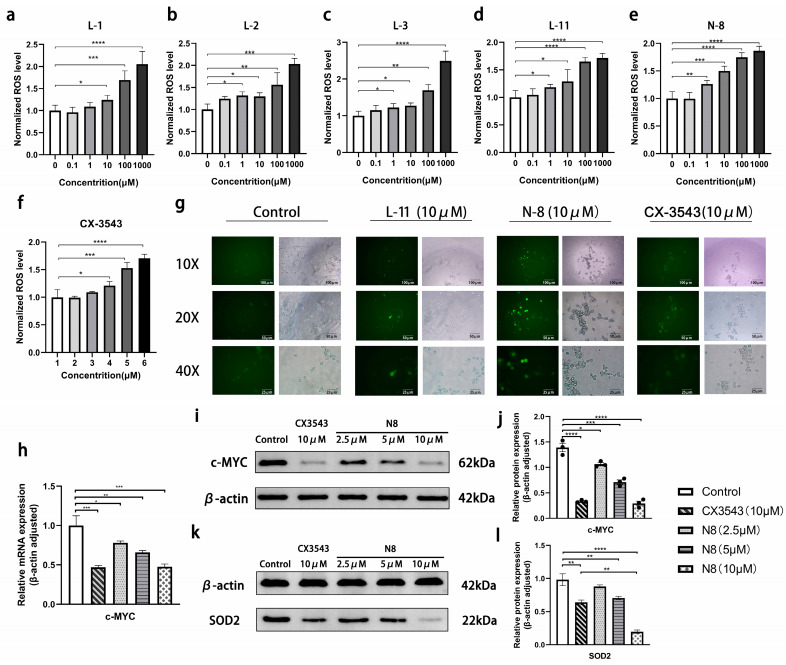
Acridone derivatives induce reactive oxygen species production in the MDA-MB-231 cell line. (**a**–**f**) The ability of compounds L-**1**, L-**2**, L-**3**, L-**11**, N-**8**, CX-**3543** in different concentrations (0 μM, 0.1 μM, 1 μM, 10 μM, 100 μM, 1000 μM) induce MDA-MB-231 cell to produce ROS. (* *p* < 0.05, ** *p* < 0.01, *** *p* < 0.001, **** *p* < 0.0001) (**g**) Fluorescence microscopy of control group; L-**11** and N-**8** treatment group; CX-**3543** treatment group. (**h**) *c-MYC* mRNA levels in MDA-MB-231 cells treated with CX3543 and different concentrations of N**8**. (**i**,**j**) *c-MYC* protein levels in MDA-MB-231 cells treated with CX3543 and different concentrations of N**8**. (**k**,**l**) SOD2 protein levels in MDA-MB-231 cells treated with CX3543 and different concentrations of N**8** (* *p* < 0.05, ** *p* < 0.01, *** *p* < 0.001, **** *p* < 0.0001).

To investigate whether oxidative stress played a pivotal role in the activity of acridone derivatives in the MDA-MB-231 cell line, we performed the DCFH-DA assay. As is shown in Figure 3a–e, compounds L**1**, L**2**, L**3**, L**11**, and N**8** can increase ROS production in a dose-dependent manner, with the EC_50_ (43.0 μM, 161.1 μM, 209.4 μM, 26.05 μM, 4.026 μM, respectively) (Table 1). Among these, compounds L**11** and N**8** exhibited the highest level of activity. Subsequently, we employed fluorescence microscopy to further scrutinize the disparities in DCFH-DA fluorescence between the control and experimental groups (Figure 3f). It became apparent that post-administration, both sets of cells exhibited a marked increase in fluorescence intensity compared to the control group. This observation suggests that under the influence of these compounds, there is a significant elevation in the ROS levels within MDA-MB-231 cells. This finding highlights the potential of compounds L**11** and N**8** as effective agents in modulating cellular oxidative stress. The detailed ROS data are included in the Supplementary Materials (Table S1).

### 3.4. Inhibition of Acridone Derivatives to the Growth of MDA-MB-231 Cell Line

To investigate the cytotoxic effects of all the acridone derivatives we synthesized, we treated the MDA-MB-231 cell line with all 29 compounds at different concentrations separately (0, 0.1, 1, 10, 100, 1000 μM) and analyzed them by performing the MTT assay. The IC_50_ results of all the compounds measured by MTT assay are listed in Table 1. We set 100 μM as the cutoff value. The measurements larger than 100 μM were regarded as low antitumor activity. The analysis shows that the IC_50_ of 9 compounds of session L (21 compounds totally) and 8 compounds of N series (8 compounds totally) is less than 100 μM, and that of 2 compounds of L series and 6 compounds of session N (8 compounds totally) is less than 10 μM. Altogether, the two series of compounds can significantly inhibit the proliferation of MDA-MB-231 cells, and the N series is even better. The detailed data of the MTT assay were included in the Supplementary Materials (Table S2).

### 3.5. N**8** Increases ROS Levels by Downregulating the Expression Levels of the c-MYC/SOD2

To further clarify the mechanism by which these compounds regulate intracellular ROS through *c-MYC*, we used the qRT PCR and Western blot methods to detect changes in the content of related mRNA and proteins. N**8** was selected to explore this mechanism as it is the most active compound. As shown in Figure 3h, the results of the qRT-PCR method indicate that N**8** can downregulate the mRNA of *c-MYC* in MDA-MB-231 cells. The Western blot results showed that the content of *c-MYC* protein in cells also decreased after N**8** treatment (Figure 3i,j and Figure S59). After comparing the above results with the positive drug CX-**3543**, we found that the inhibitory effect of N**8** on *c-MYC* was similar to CX-**3543** and even better than CX3543 at low concentrations. SOD2 is a crucial gene regulated by *c-MYC* and encodes superoxide dismutase, a key enzyme involved in clearing harmful oxidative substances within cells. Reduced SOD2 expression leads to an increase in intracellular ROS levels, inducing apoptosis [29,30]. We assessed the impact of CX-**3543** and different concentrations of N**8** on SOD2 expression using the Western blot method and found a correlation between *c-MYC* expression decreases and SOD2 expression decreases (Figure 3k,l and Figure S60). The experimental results suggest that the compound N**8** can stimulate the ROS generation by regulating the expression of *c-MYC*/SOD2.

## 4. Discussion

The transcription factor *c-MYC* plays a pivotal role in regulating fundamental cellular mechanisms, including but not limited to controlling the cell cycle, modulating apoptosis, promoting protein synthesis, managing cell adhesion, and overseeing various other critical biological processes [31,32,33]. An elevated expression of *c-MYC* is observed in various tumors, marking it as a key oncogene. Theoretically, designing drugs targeting the *c-MYC* protein holds significant promise. However, akin to other transcription factors, *c-MYC* lacks a distinct binding site for potential regulators, compounded by its remarkably short half-life of only 20–30 min [34]. These characteristics pose a formidable challenge in devising compounds capable of directly interacting with the *c-MYC* protein. Consequently, exploiting the G4 structure of *c-MYC* as a novel target for developing anticancer therapeutics holds substantial research value in oncology, with current drug development efforts predominantly concentrated in this arena.

Despite the significance of *c-MYC* G4 as a therapeutic target, it is crucial to note that G4 structures are not exclusive to *c-MYC*. Medications designed to influence *c-MYC* gene transcription may inadvertently impact the expression of other genes, giving rise to a cascade of adverse reactions. This inherent challenge significantly complicates the development of *c-MYC* G4 stabilizers, contributing to the current state where drugs in this category remain confined to clinical trial phases. The dual challenge of achieving specificity while avoiding unintended consequences underscores the intricate nature of advancing *c-MYC*-targeted therapeutic interventions. As research continues, overcoming these hurdles will pave the way for innovative and precise anticancer treatments.

In our investigation, the cell activity results indicate that compounds from the N series generally exhibit superior activity compared to those from the L series, suggesting that oxazole is favorable for activity as a linker between the core pyridinone and the aromatic group. A comparison between compound N**1** and compound L**4** supports this conclusion, with strong evidence also provided by N**2** and L**2**. Among compounds with triazoles, L**11** stands out as the most active, featuring a carboxylic ester adjacent to a methyl-substituted aromatic group. The length of the ester bond significantly impacts molecular activity, with shorter ester bonds resulting in higher activity. However, when the ester bond is removed to create derivatives with shorter carboxylic groups, such as L**12**, activity is noticeably reduced to as low as one-tenth of that of L**11**. Further support comes from L**19** and L**20**. Moreover, compounds with meta-methyl substitutions generally exhibit higher activity compared to those without methyl groups, as seen with L**11** having ten times higher activity than L**20** and L**12**, exhibiting higher activity than L**19**, suggesting a contribution of meta-methyl substitutions to activity. Nevertheless, for compounds with carboxylic esters bearing aromatic groups in ortho positions, the relationship between ester length and activity appears irregular.

Compounds without aromatic groups, such as L**5**–L**9**, are derivatives of ethyl esters linked with triazoles. Among these compounds, L**9** exhibits the highest activity, with only a positional difference in the carbonyl group compared to L**5**, resulting in a 20-fold difference in activity. The molecular docking results show that the pyridinone core of L**9** and the triazole interact with DG7, DG11, and DA6, while the carbonyl of L**5** can form hydrogen bonds with 6DA bases. However, this hydrogen bond seems to induce a change in the position of the entire molecule, preventing interactions between the pyridinone core and DG7 or DG11, possibly explaining the disparity in their activities.

In the N series of compounds, where thiazole substitutes for triazole, the most active compounds are N**8**, N**4**, and N**7**, with substituents on the phenyl ring affecting activity. Compounds with ortho substitutions exhibit higher activity than those with para substitutions, and this activity difference appears unrelated to the electronegativity of the substituent, as observed with N**6** showing higher activity than N**2** and N**8** exhibiting higher activity than N**4**. This trend aligns with the L series of compounds, suggesting a common mode of interaction with the receptor.

To further investigate the structure–activity relationship, we computed molecular descriptors for each compound and established a regression model:

QSAR model:

$${IC}_{50}=11.3409\times {SlogP}_{VSA5}-2.7421\times {ASA}_{p}-2.4401\times E_{rele}+2.3907\times E_{stb}+5.0351$$

where SlogP represents the compound’s partition coefficient between lipids and water, which is positively correlated with the IC_50_ value for small molecules. This suggests that the lipophilicity of compounds is favorable for activity, explaining why carboxylic derivatives are less active compared to their corresponding carboxyl esters. The electrostatic interaction energy of molecules refers to the energy generated by the mutual attraction or repulsion of charges between atoms within a molecule. If the charge distribution within the molecule is uneven, it complicates electrostatic interactions. Polar molecules (those with positively and negatively charged regions) generally have stronger electrostatic interaction energy because of their uneven charge distribution. This indicator is negatively correlated with the IC_50_ value, indicating that fewer heteroatoms or simpler substituents are advantageous for activity. Compounds in the N series, both in the linker and aromatic segments, contain fewer heteroatoms than L series compounds, explaining their generally higher activity.

**Figure 4:**
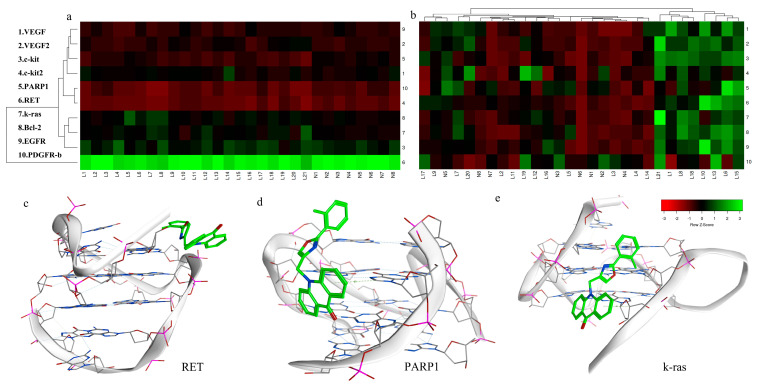
Heat map of selective results of small molecules towards other DNA-G4 structures: (**a**) receptor-based clustering; (**b**) small molecule-based clustering; Z-score is used to evaluate the distance between sample points and population mean), as well as docking results (green molecule: compound N**8**, gray white molecule and ribbon: the G4 structure of (**c**) RET; (**d**) PARP1; (**e**) k-ras, respectively).

In addition to MYC, there are other genes in the genome capable of forming G-quadruplex (G4) structures; the selectivity of MYC-G4 stabilizers is a crucial metric for assessing their performance. In order to further explore the selectivity patterns of the small molecules we have synthesized, we conducted an initial selectivity assessment using molecular docking. We compiled a list of ten genes, apart from MYC, known to form G4 structures, and used them as receptors for docking with the small molecules synthesized in this study (Figure 4; Table S3). The results revealed that, from the receptor perspective, derivatives of pyridinone exhibited significant selectivity towards RET (PDB ID: 7YS7), c-kit (PDB ID: 2O3M), VEGF (PDB ID: 2M27), k-ras (PDB ID: 7X8N), and Bcl-2 (PDB ID: 6ZX7), especially in the case of the RET gene, where all molecules demonstrated selectivity exceeding a tenfold difference. Conversely, selectivity was notably lower with PDB ID: 7NWD and PDB ID: 6V01. From the ligand perspective, it appears that this may partly explain why certain compounds exhibited strong activity in cellular experiments but lacked activity in ROS experiments. For instance, compounds L**20**, N**1**, N**3**, N**6**, and N**7** may be targeting alternative pathways beyond MYC, inhibiting tumor cell proliferation.

In the experiments detailed in this study, by assessing the levels of the transcription factor *c-MYC* and its target gene product SOD2, we observed a significant decrease in the content of the *c-MYC* transcription factor and a corresponding downregulation of its target gene SOD2 expression following treatment with N**8** in breast cancer cells. Despite the adaptation of breast cancer cells to elevated levels of ROS beyond normal levels, the accumulation of ROS can still induce apoptosis in tumor cells if intracellular ROS are not promptly cleared [35,36]. We have confirmed the effectiveness of certain compounds in inhibiting the *c-MYC* protein within MDA-MB-231 cells, resulting in suppressed proliferation, elevated intracellular ROS levels, and the induction of apoptosis. Our findings indicate that compounds based on the acridone scaffold have immense potential as stabilizers of *c-MYC*’s G4 structure. Notably, the N-**8** compound exhibited the most significant activity and demonstrated a high degree of selectivity for the G4 structure of the *c-MYC* gene, presenting itself as a promising candidate for a novel therapy for triple-negative breast cancer. It is worth mentioning that, in the Western blot experiments, N**8** exhibited higher activity at low concentrations. We speculate that this may be attributed to its poor solubility, resulting in the actual concentration of the high-dose group not being as high as indicated. In other words, the activity of N**8** may actually be superior to CX-**3543**, providing a crucial direction for our upcoming structural optimization studies.

Moreover, leveraging the acridone scaffold opens avenues for designing more compounds targeting *c-MYC*’s G4 structure, thereby providing additional potential options for treating triple-negative breast cancer. In the context of our research design, the strength of this study lies in targeting triple-negative breast cancer, which is commonly associated with poor treatment outcomes and a relative lack of therapeutic targets. By focusing on the pivotal gene *c-MYC*, we have designed and synthesized a novel series of compounds, demonstrating high anti-tumor activity and selectivity in preliminary experiments. However, the solubility of these small molecules is not promising enough, as mentioned in the above discussion, which is a key factor limiting their further research, such as in vivo experiments and ADMET experiments. Addressing these gaps in our research constitutes a crucial aspect of our next steps.

## 5. Conclusions

In pursuit of functional molecules with therapeutic potential against TNBC, a series of acridone N-substituted derivatives were synthesized to modulate intracellular ROS levels. The variations in cellular ROS content and their inhibitory effects on TNBC were evaluated. Notably, compounds L**11**, N**7**, and N**8** exhibited significant potentiation of TNBC suppression. In light of the regulatory relationship between MYC gene expression and intracellular ROS levels, promising candidates were identified. By detecting the expression of related genes and proteins, the mechanism of the promising candidate increasing intracellular ROS and inducing apoptosis of breast cancer cells through the *c-MYC*/SOD2 pathway was clarified. Utilizing QSAR modeling, a structure-activity relationship study was conducted, revealing that lipophilic groups with fewer heteroatoms constitute advantageous pharmacophores. Computational docking studies provided insights into the selectivity of MYC-G4 stabilizers. In conclusion, our endeavors highlight compounds L**11** and N**8** as potential small molecules for promoting TNBC apoptosis through ROS modulation, offering a promising avenue for the treatment of TNBC.

## Figures and Tables

**Table 1:**
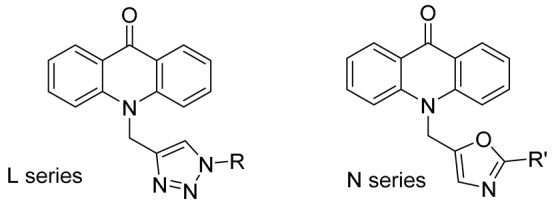
EC_50_ (ROS) and IC_50_ (MTT) of 30 compounds to MDA-MB-231 cell lines.

Compound	R	EC_50_ (95%CI) ROS	IC_50_ (95%CI) MTT	Compound	R	EC_50_ (95%CI) ROS	IC_50_ (95%CI) MTT
L**1**	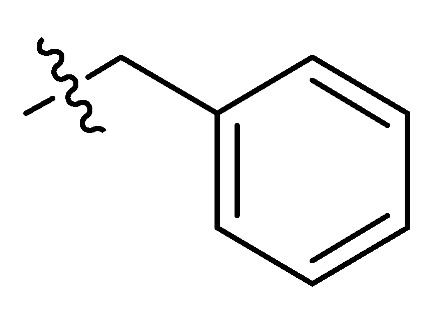	43.0 μM (10.48–95.29)	33.28 μM (16.46–66.36)	L**16**	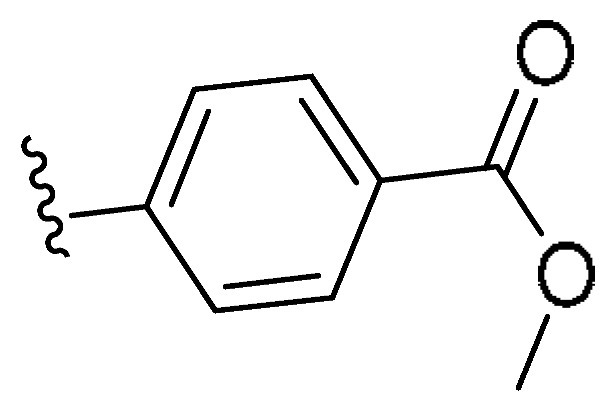	--	\
L**2**	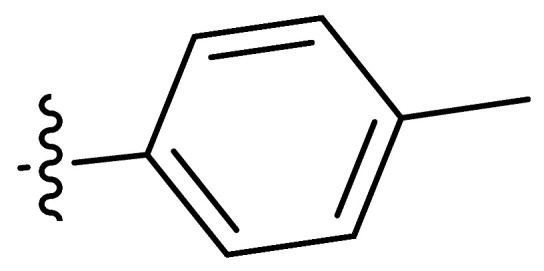	161.1 μM (54.01–523.5)	\ ^2^	L**17**	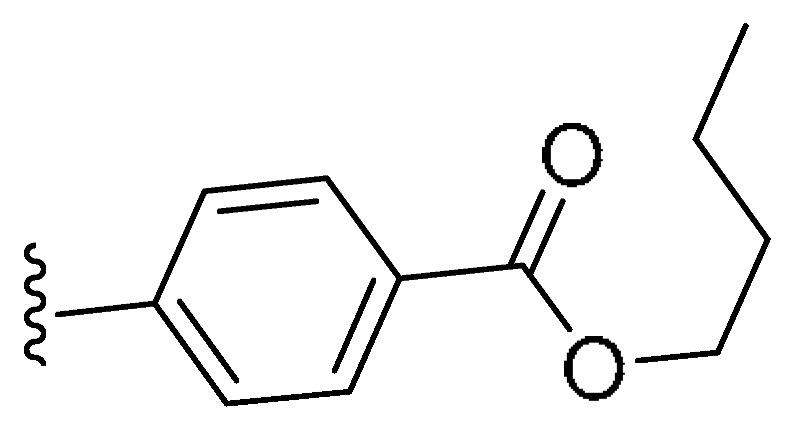	--	\
L**3**	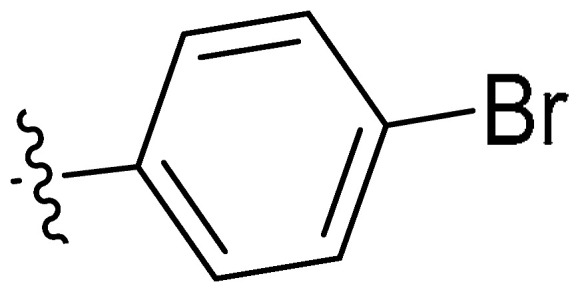	209.4 μM (95.68–425.7)	\	L**18**	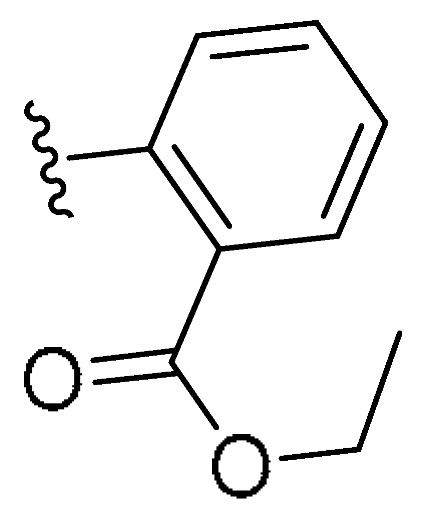	--	\
L**4**	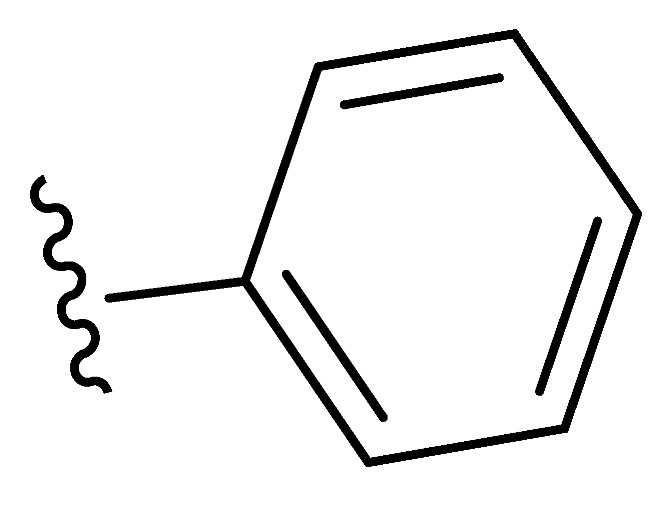	-- ^1^	\	L**19**	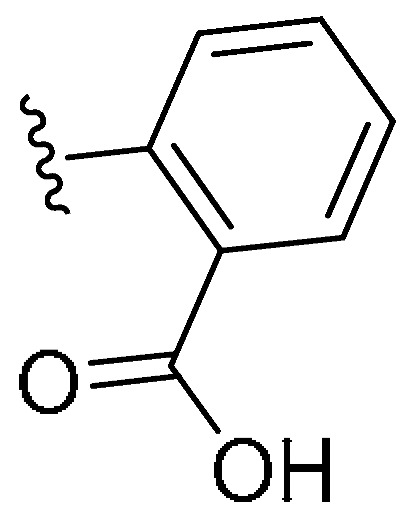	--	\
L**5**	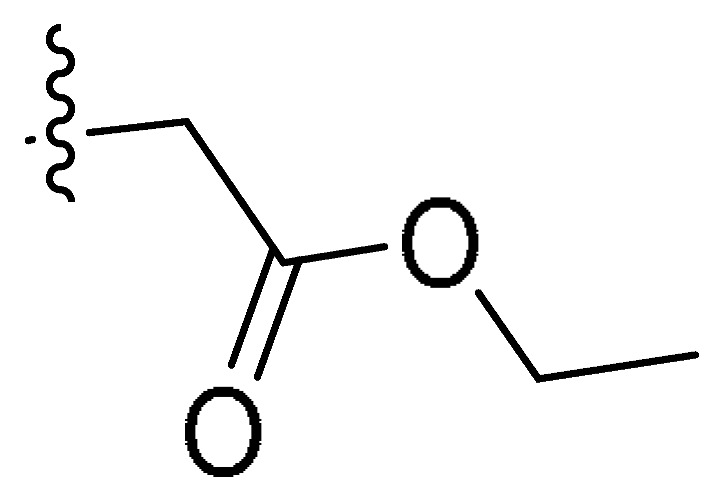	--	\	L**20**	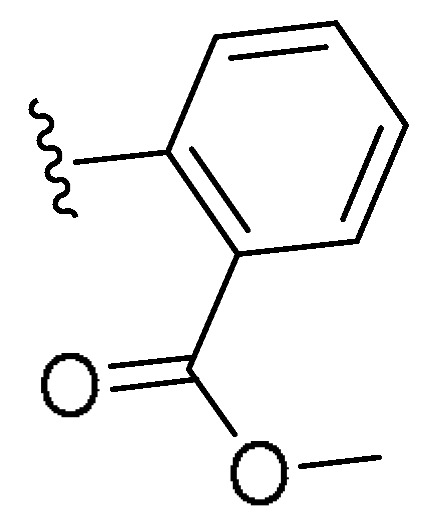	--	22.95 μM (5.758–87.58)
L**6**	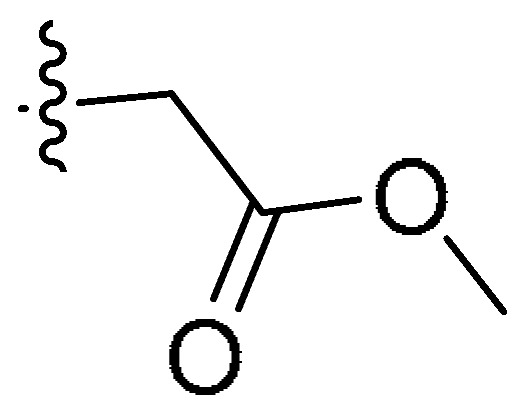	--	\	L**21**	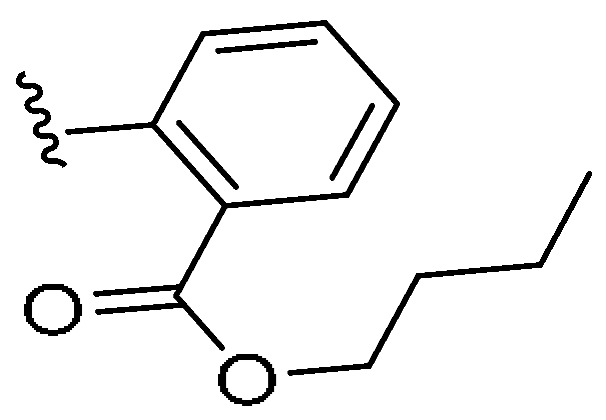	--	\
L**7**	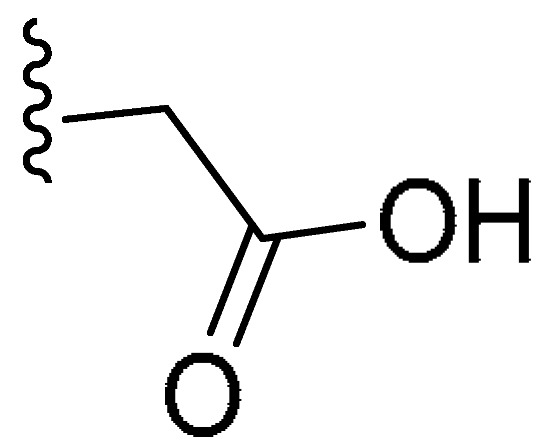	--	\	N**1**	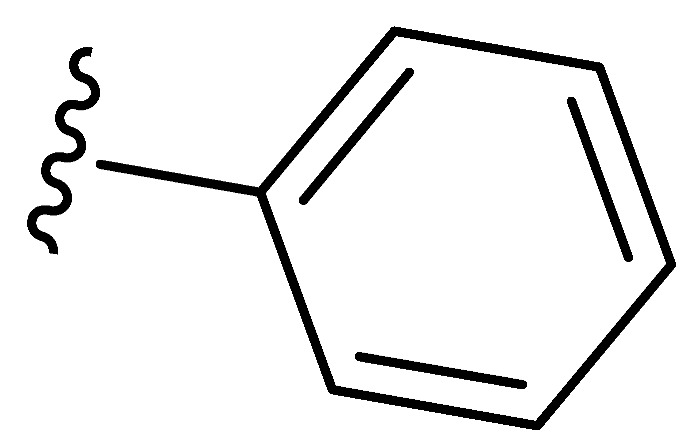	--	7.581 μM (4.268–13.32)
L**8**	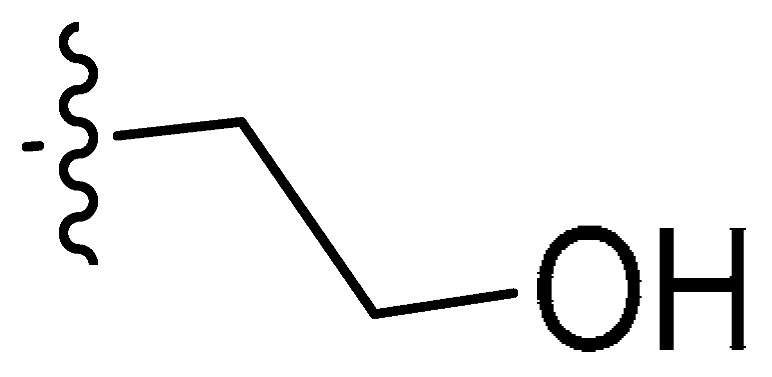	--	\	N**2**	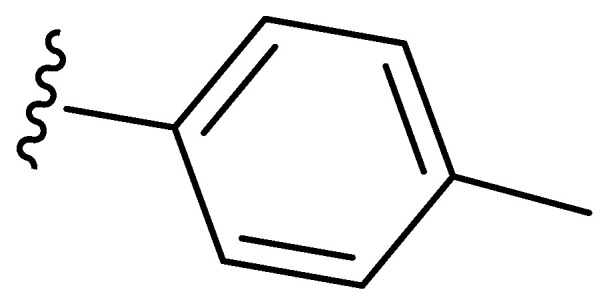	--	6.017 μM (1.284–25.11)
L**9**	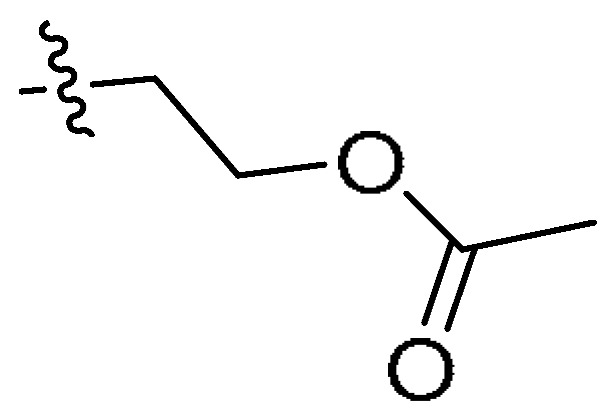	--	21.61 μM (11.95–31.96)	N**3**	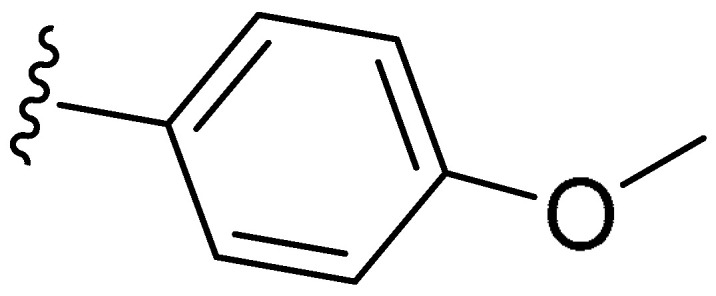	--	71.99 μM (10.02–911.1)
L**10**	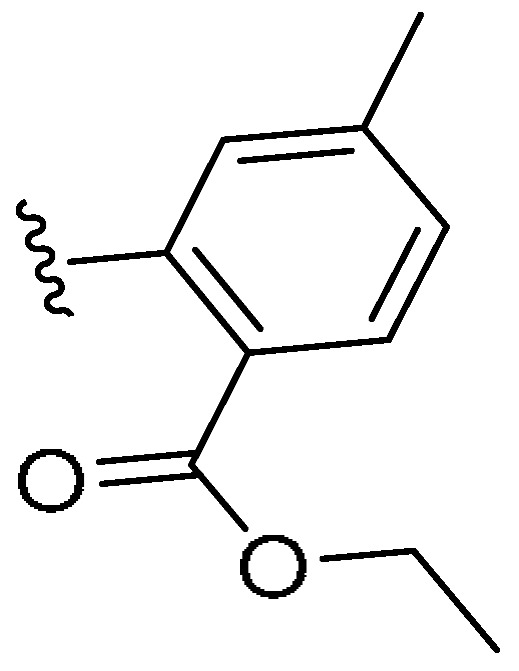	--	\	N**4**	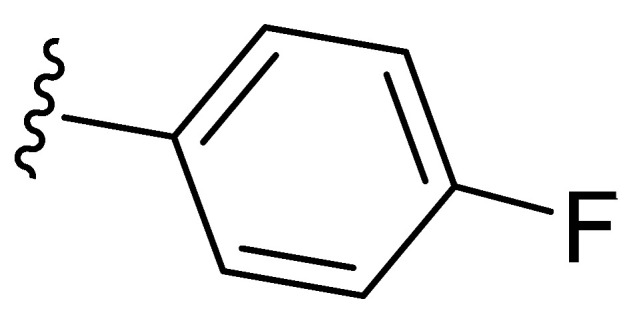	--	1.003 μM (0.5610–1.698)
L**11**	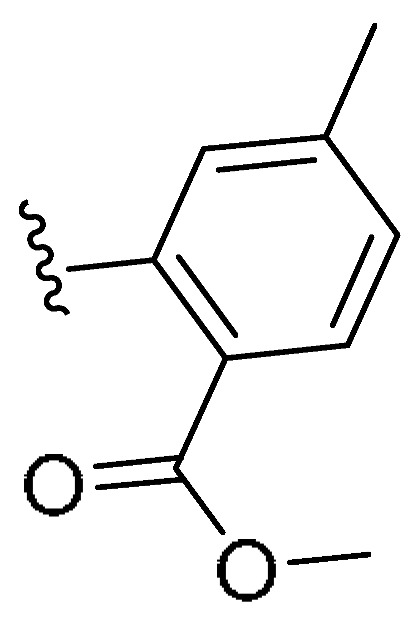	26.05 μM (12.36–57.80)	2.377 μM (1.249–4.366)	N**5**	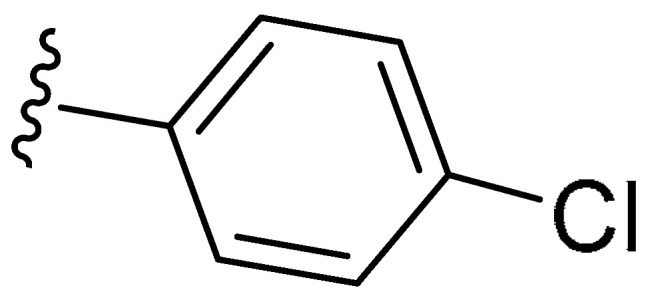	--	42.86 μM (12.18–143.4)
L**12**	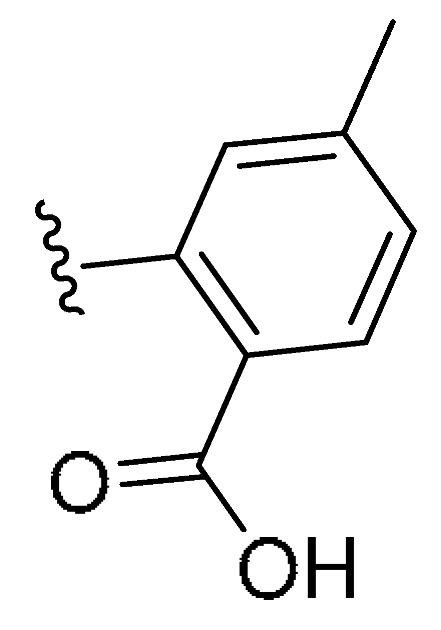	--	20.89 μM (10.98–39.64)	N**6**	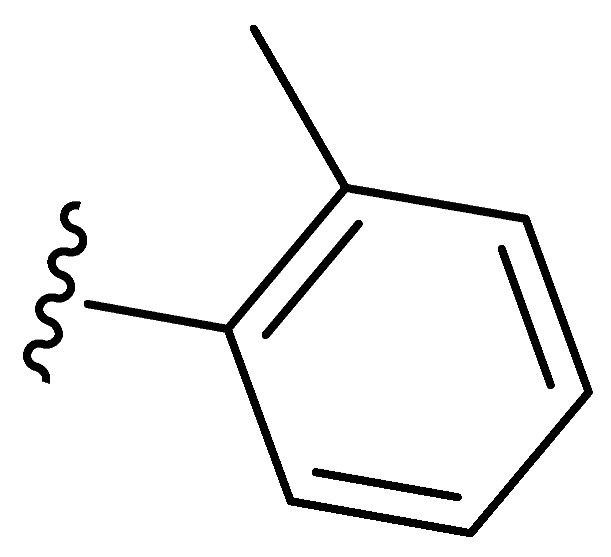	--	2.638 μM (0.7022–8.810)
L**13**	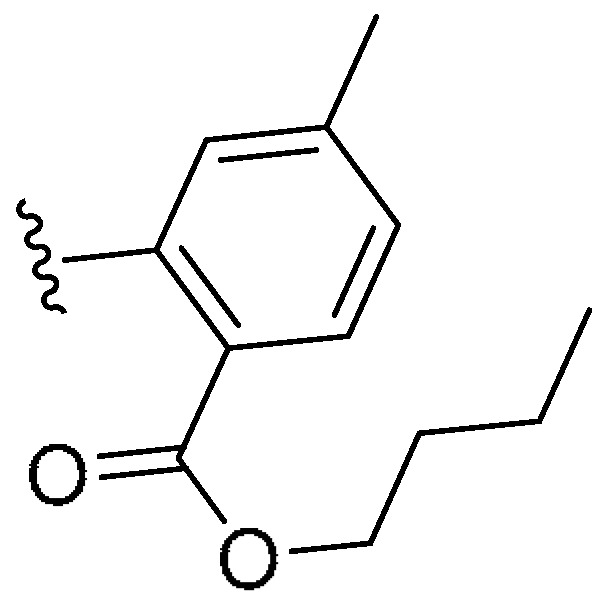	--	\	N**7**	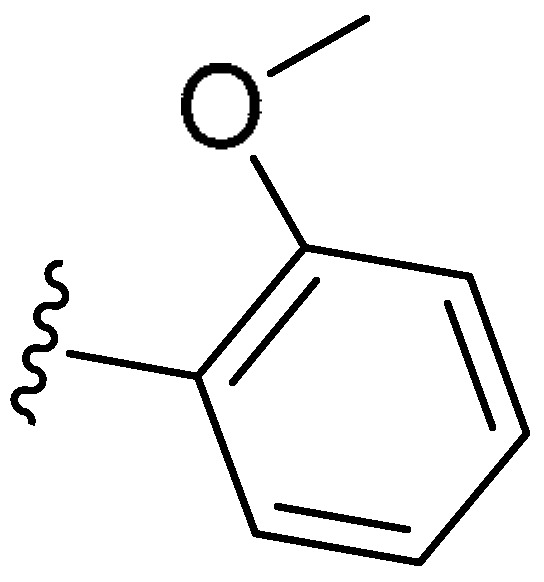	--	0.8802 μM (0.3418–1.916)
L**14**	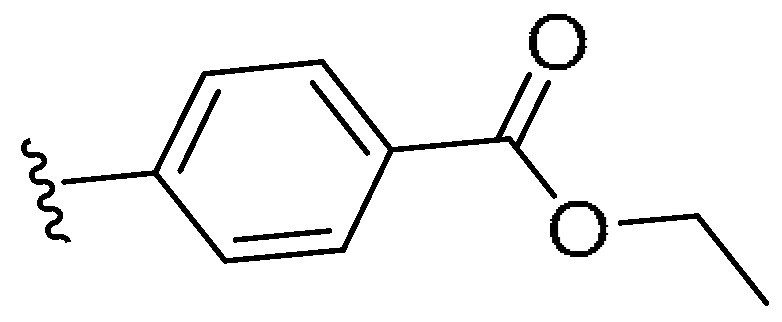	--	12.76 μM (3.52–43.96)	N**8**	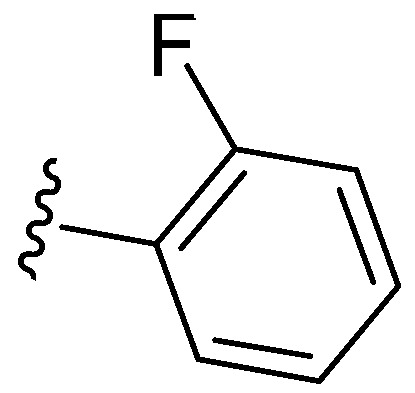	4.026 μM (1.299–13.46)	0.7222 μM (0.003539–6.103)
L**15**	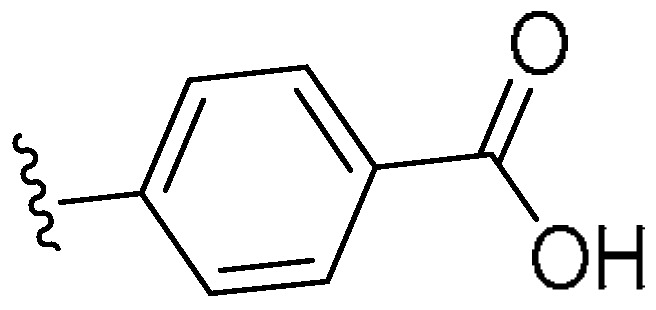	--	75.41 μM (23.41–238.1)	CX-**3543**		37.48 μM (13.8–104.2)	1.934 μM (0.9373–3.924)

^1^ “--”: EC_50_ (ROS) > 300 μM or not available. ^2^ “\”: IC_50_ (MTT) > 100 μM.

## Data Availability

Data are contained within the article and supplementary materials.

## Supplementary Materials

The following supporting information can be downloaded at: https://www.mdpi.com/article/10.3390/antiox13010011/s1, Scheme S1: The NMR data of our compounds and the additional synthesis steps required for L**6**–L**9**. Figure S1: ^13^C NMR of compound L**1**; Figure S2: ^1^H NMR of compound L**1**; Figure S3: ^13^C NMR of compound L**2**; Figure S4: ^1^H NMR of compound L**2**; Figure S5: ^13^C NMR of compound L**3**; Figure S6: ^1^H NMR of compound L**3**; Figure S7: ^13^C NMR of compound L**4**; Figure S8: ^1^H NMR of compound L**4**; Figure S9: ^13^C NMR of compound L**5**; Figure S10: ^1^H NMR of compound L**5**; Figure S11: ^13^C NMR of compound L**6**; Figure S12: ^1^H NMR of compound L**6**; Figure S13: ^13^C NMR of compound L**7**; Figure S14: ^1^H NMR of compound L**7**; Figure S15: ^13^C NMR of compound L**8**; Figure S16: ^1^H NMR of compound L**8**; Figure S17: ^13^C NMR of compound L**9**; Figure S18: ^1^H NMR of compound L**9**; Figure S19: ^13^C NMR of compound L**10**; Figure S20: ^1^H NMR of compound L**10**; Figure S21: ^13^C NMR of compound L**11**; Figure S22: ^1^H NMR of compound L**11**; Figure S23: ^13^C NMR of compound L**12**; Figure S24: ^1^H NMR of compound L**12**; Figure S25: ^13^C NMR of compound L**13**; Figure S26: ^1^H NMR of compound L**13**; Figure S27: ^13^C NMR of compound L**14**; Figure S28: ^1^H NMR of compound L**14**; Figure S29: ^13^C NMR of compound L**15**; Figure S30: ^1^H NMR of compound L**15**; Figure S31: ^13^C NMR of compound L**16**; Figure S32: ^1^H NMR of compound L**16**; Figure S33: ^13^C NMR of compound L**17**; Figure S34: ^1^H NMR of compound L**17**; Figure S35: ^13^C NMR of compound L**18**; Figure S36: ^1^H NMR of compound L**18**; Figure S37: ^13^C NMR of compound L**19**; Figure S38: ^1^H NMR of compound L**19**; Figure S39: ^13^C NMR of compound L**20**; Figure S40: ^1^H NMR of compound L**20**; Figure S41: ^13^C NMR of compound L**21**; Figure S42: ^1^H NMR of compound L**21**; Figure S43: ^13^C NMR of compound N**1**; Figure S44: ^1^H NMR of compound N**1**; Figure S45: ^13^C NMR of compound N**2**; Figure S46: ^1^H NMR of compound N**2**; Figure S47: ^13^C NMR of compound N**3**; Figure S48: ^1^H NMR of compound N**3**; Figure S49: ^13^C NMR of compound N**4**; Figure S50: ^1^H NMR of compound N**4**; Figure S51: ^13^C NMR of compound N**5**; Figure S52: ^1^H NMR of compound N**5**; Figure S53: ^13^C NMR of compound N**6**; Figure S54: ^1^H NMR of compound N**6**; Figure S55: ^13^C NMR of compound N**7**; Figure S56: ^1^H NMR of compound N**7**; Figure S57: ^13^C NMR of compound N**8**; Figure S58: ^1^H NMR of compound N**8**; Figure S59: *c-MYC* expression in CX3543 or different concentration of N**8** treated MDA-MB-231 cells. (The full image of Figure 3i); Figure S60: SOD2 expression in CX3543 or different concentrations of N**8**-treated MDA-MB-231 cells. (The full image of Figure 3k); Table S1: Detailed data of ROS test by microplate reader; Table S2: Detailed data of MTT test by microplate reader; Table S3: Scores of molecular docking by Acridone derivatives and diverse genes with G4 structure.
