# Supplementary material for: Development of Acridone Derivatives: Targeting c-MYC Transcription in Triple-Negative Breast Cancer with Inhibitory Potential
Source: Antioxidants (Basel). 2023 Dec 20;13(1):11. doi: 10.3390/antiox13010011 (PMC10812579; doi:10.3390/antiox13010011)
Supplement: Supplementary file 1 [file antioxidants-13-00011-s001.zip › Scheme S1&Figure S1-S60&Table S3.pdf]

Scheme S1.The NMR data of our compounds and the additional synthesis steps required for L6-L9.

10-((1-benzyl-1H-1,2,3-triazol-4-yl)methyl)acridin-9(10H)-one (L1)

^{1}H NMR (600 MHz, DMSO-d_6) δ 8.36 (dd, *J* = 7.8, 1.8 Hz, 2H), 8.21 (s, 1H), 7.95 (d, *J* = 8.7 Hz, 2H), 7.84 – 7.78 (m, 2H), 7.37 – 7.32 (m, 4H), 7.31 (d, *J* = 6.7 Hz, 1H), 7.28 – 7.24 (m, 2H), 5.80 (s, 2H), 5.54 (s, 2H). ^{13}C NMR (151 MHz, DMSO-d_6) δ 177.10, 143.28, 142.29, 136.41, 134.64, 129.18, 128.58, 128.36, 127.11, 124.00, 122.20, 121.98, 116.78, 53.29, 42.13.

10-((1-(p-tolyl)-1H-1,2,3-triazol-4-yl)methyl)acridin-9(10H)-one (L2)

^{1}H NMR (600 MHz, DMSO-d_6) δ 2.34 (s, 3 H), 5.87 (s, 2 H), 7.30 - 7.42 (m, 4 H), 7.72 (d, *J* = 8.28 Hz, 2 H), 7.82 (t, *J* = 7.34 Hz, 2 H), 7.96 (d, *J* = 8.66 Hz, 2 H), 8.37 (d, *J* = 7.34 Hz, 2 H), 8.78 (s, 1 H); ^{13}C NMR (151 MHz, DMSO-d_6) δ 20.9, 40.5, 42.2, 116.7, 120.3, 121.8, 121.9, 122.2, 127.0, 130.5, 134.6, 134.6, 138.7, 142.3, 144.1, 177.1

10-((1-(4-bromophenyl)-1H-1,2,3-triazol-4-yl)methyl)acridin-9(10H)-one (L3)

^{1}H NMR (600 MHz, DMSO-d_6) δ 5.89 (s, 2 H), 7.36 (t, *J* = 7.34 Hz, 2 H), 7.72 - 7.76 (m, *J* = 8.66 Hz, 2 H), 7.80 - 7.85 (m, 4 H), 7.91 - 7.98 (m, *J* = 8.66 Hz, 2 H), 8.37 (d, *J* = 7.53 Hz, 2 H), 8.85 (s, 1 H); ^{13}C NMR (151 MHz, DMSO-d_6) δ 42.2, 116.7, 121.7, 121.9, 122.0, 122.2, 122.3, 127.0, 133.1, 134.7, 136.0, 142.3, 144.4, 177.1

10-((1-phenyl-1H-1,2,3-triazol-4-yl)methyl)acridin-9(10H)-one (L4)

^{1}H NMR (600 MHz, DMSO-d_6) δ 5.89 (s, 2 H), 7.33 - 7.39 (m, 2 H), 7.42 - 7.48 (m, 1 H), 7.52 - 7.58 (m, 2 H), 7.79 - 7.87 (m, 4 H), 7.96 (d, *J* = 8.85 Hz, 2 H), 8.37 (dd, *J* = 8.00, 1.60 Hz, 2 H), 8.83 (s, 1 H); ^{13}C NMR (151 MHz, DMSO-d_6) δ 42.2, 116.7, 120.4, 121.9, 122.0, 122.2, 127.0, 129.1, 130.2, 134.6, 136.8, 142.3, 144.2, 177.1.

ethyl 2-(4-((9-oxoacridin-10(9H)-yl)methyl)-1H-1,2,3-triazol-1-yl)acetate (L5)

^{1}H NMR (600 MHz, DMSO-d_6) δ 1.15 (t, *J* = 7.15 Hz, 3 H), 4.11 (q, *J* = 7.09 Hz, 2 H), 5.31 (s, 2 H), 5.85 (s, 2 H), 7.35 (t, *J* = 7.34 Hz, 2 H), 7.82 (ddd, *J* = 8.66, 6.96, 1.69 Hz, 2 H), 7.96 (d, *J* = 8.66 Hz, 2 H), 8.10 (s, 1 H), 8.36 (dd, *J* = 7.91, 1.69 Hz, 2 H); ^{13}C NMR (151 MHz, DMSO-d_6) δ 14.3, 41.8, 50.8, 61.8, 116.7, 121.9, 122.1, 125.2, 127.0, 134.6, 142.1, 142.9, 167.5, 177.0

methyl 2-(4-((9-oxoacridin-10(9H)-yl)methyl)-1H-1,2,3-triazol-1-yl)acetate(L6)

To a solution of 12 (1 mmol) in MeCN (10 mL) at room temperature, K_2CO_3 (2 mmol) was added. The reaction mixture was refluxed for 0.5 h. Then, the mixture was diluted with water (10 mL) and filtered to give the crude product, and the crude product was purified by column chromatography to afford product 13 (68 %). ^{1}H NMR (600 MHz, DMSO-d_6) δ 3.66 (s, 3 H), 5.34 (s, 2 H), 5.85 (s, 2 H), 7.30 - 7.38 (m, 2 H), 7.83 (ddd, *J* = 8.66, 6.96, 1.69 Hz, 2 H), 7.96 (d, *J* = 8.85 Hz, 2 H), 8.11 (s, 1 H), 8.36 (dd, *J* = 7.91, 1.69 Hz, 2 H); ^{13}C NMR (151 MHz, DMSO-d_6) δ 41.8, 50.6, 52.9, 116.7, 121.9, 122.1, 125.2, 127.0, 134.6, 142.1, 142.9, 168.0, 177.0.

2-(4-((9-oxoacridin-10(9H)-yl)methyl)-1H-1,2,3-triazol-1-yl)acetic acid(L7)

To a solution of 11 (1 mmol) in EtOH/H2O (1:10, 10 mL) at room temperature, NaOH (0.5 mmol) was added. The reaction mixture was stirred at 80 ℃ for 2 h until the starting material disappeared, as indicated by TLC. Then, the mixture was acidified with hydrochloric acid and filtered to give crude product, and the crude product was purified by column chromatography to afford product 12 (73 %).^{1}H NMR (600 MHz, DMSO-d_6) δ 5.17 - 5.26 (m, 2 H), 5.83 (s, 2 H), 7.34 (t, *J* = 7.44 Hz, 2 H), 7.77 - 7.86 (m, 2 H), 7.97 (d, *J* = 8.66 Hz, 2 H), 8.13 (d, *J* = 2.45 Hz, 1 H), 8.32 - 8.40 (m, 2 H), 13.36 (br. s., 1 H); ^{13}C NMR (151 MHz, DMSO-d_6) δ 41.8, 50.9, 116.7, 121.9, 122.1, 125.1, 127.0, 134.6, 142.1, 142.7, 168.8, 177.0.

10-((1-(2-hydroxyethyl)-1H-1,2,3-triazol-4-yl)methyl)acridin-9(10H)-one(L8)

To a solution of 11 (1 mmol) in MeOH (15 mL) at room temperature, $NaBH_4$ (10 mmol) was added. The reaction mixture was stirred at room temperature for 1 h until the starting material disappeared, as indicated by TLC. Then, the mixture was diluted with water (10 mL) and filtered to give the crude product, and the crude product was purified by column chromatography to afford product 13 (55 %).^{1}H NMR (600 MHz, DMSO-d_6) δ 3.71 (t, *J* = 5.36 Hz, 2 H), 4.34 (t, *J* = 5.36 Hz, 2 H), 4.96 (br. s., 1 H), 5.79 (s, 2 H), 7.35 (t, *J* = 7.43 Hz, 2 H), 7.82 (ddd, *J* = 8.66, 6.96, 1.69 Hz, 2 H), 7.97 (d, *J* = 8.66 Hz, 2 H), 8.08 (s, 1 H), 8.35 (dd, *J* = 7.91, 1.51 Hz, 2 H); ^{13}C NMR (151 MHz, DMSO-d_6) δ 42.0, 52.6, 60.1, 116.8, 121.9, 122.1, 124.1, 127.0, 134.6, 142.2, 142.5, 177.0.

2-(4-((9-oxoacridin-10(9H)-yl)methyl)-1H-1,2,3-triazol-1-yl)ethyl acetate (L9)

To a solution of 14 (1 mmol) in THF (10 mL), Ac_2O (1.1 mmol) was added. The reaction mixture was refluxed for 0.5 h until the starting material disappeared as indicated by TLC. Then, the mixture was diluted with water (10 mL) and filtered to give the crude product, and the crude product was purified by column chromatography to afford product 13 (87 %). ^{1}H NMR (600 MHz, DMSO-d_6) *δ* 1.85 (s, 3 H), 4.32 (t, *J* = 5.18 Hz, 2 H), 4.56 (t, *J* = 5.18 Hz, 2 H), 5.80 (s, 2 H), 7.35 (t, *J* = 7.34 Hz, 2 H), 7.81 (ddd, *J* = 8.61, 7.01, 1.69 Hz, 2 H), 7.94 (d, *J* = 8.85 Hz, 2 H), 8.13 (s, 1 H), 8.35 (dd, *J* = 8.09, 1.51 Hz, 2 H); ^{13}C NMR (151 MHz, DMSO-d_6) *δ* 20.8, 42.0, 48.9, 62.5, 116.7, 121.9, 122.1, 124.3, 127.0, 134.5, 142.2, 142.9, 170.2, 177.0.

ethyl 4-methyl-2-(4-((9-oxoacridin-10(9H)-yl)methyl)-1H-1,2,3-triazol-1-yl)benzoate (L10)

^{1}H NMR (600 MHz, DMSO-d6) δ 8.61 (s, 1H), 8.36 (dd, J = 8.0, 1.7 Hz, 2H), 8.05 (d, J = 8.8 Hz, 2H), 7.84 (ddd, J = 8.7, 6.9, 1.8 Hz, 2H), 7.68 (d, J = 2.0 Hz, 1H), 7.55 (dd, J = 8.3, 2.0 Hz, 1H), 7.48 (d, J = 8.1 Hz, 1H), 7.36 (t, J = 7.4 Hz, 2H), 5.89 (s, 2H), 3.82 (q, J = 7.1 Hz, 2H), 2.41 (s, 3H), 0.68 (t, J = 7.1 Hz, 3H). ^{13}C NMR (151 MHz, DMSO-d6) δ 177.13, 165.68, 143.10, 142.31, 140.62, 134.60, 133.60, 133.20, 131.20, 127.79, 127.11, 126.69, 125.44, 122.24, 121.99, 116.91, 61.45, 41.76, 20.90, 13.74.

methyl 4-methyl-2-(4-((9-oxoacridin-10(9H)-yl)methyl)-1H-1,2,3-triazol-1-yl)benzoate (L11)

^{1}H NMR (600 MHz, DMSO-d_6) *δ* 8.58 (s, 1H), 8.38 (dd, *J* = 8.0, 1.7 Hz, 2H), 8.01 (d, *J* = 8.7 Hz, 2H), 7.85 (ddd, *J* = 8.7, 6.8, 1.8 Hz, 2H), 7.71 (d, *J* = 2.0 Hz, 1H), 7.55 (dd, *J* = 8.1, 2.2 Hz, 1H), 7.50 (d, *J* = 8.1 Hz, 1H), 7.37 (t, *J* = 7.4 Hz, 2H), 5.90 (s, 2H), 3.43 (s, 3H), 2.41 (s, 3H). ^{13}C NMR (151 MHz, DMSO-d_6) *δ* 177.17, 165.99, 143.18, 142.36, 140.58, 134.64, 133.72, 133.30, 131.22, 127.25, 127.10, 126.74, 125.43, 122.26, 121.99, 116.87, 52.59, 42.04, 20.89.

4-methyl-2-(4-((9-oxoacridin-10(9H)-yl)methyl)-1H-1,2,3-triazol-1-yl)benzoic acid (L12)

^{1}H NMR (600 MHz, DMSO-d_6) *δ* 8.59 (s, 1H), 8.37 (dd, *J* = 8.0, 1.8 Hz, 2H), 8.02 (d, *J* = 8.7 Hz, 2H), 7.83 (ddd, *J* = 8.8, 6.8, 1.7 Hz, 2H), 7.70 (d, *J* = 2.1 Hz, 1H), 7.49 (dd, *J* = 8.1, 2.0 Hz, 1H), 7.42 (d, *J* = 8.1 Hz, 1H), 7.36 (t, *J* = 7.4 Hz, 2H), 5.86 (s, 2H), 2.41 (s, 3H). ^{13}C NMR (151 MHz, DMSO-d_6) *δ* 177.16, 167.09, 142.77, 142.42, 140.29, 134.65, 133.28, 132.88, 131.14, 129.25, 127.06, 126.80, 125.65, 122.22, 121.97, 116.94, 42.14, 20.95.

butyl 4-methyl-2-(4-((9-oxoacridin-10(9H)-yl)methyl)-1H-1,2,3-triazol-1-yl)benzoate (L13)

^{1}H NMR (600 MHz, DMSO-d_6) *δ* 8.68 (s, 1H), 8.38 (d, *J* = 8.0 Hz, 2H), 8.07 (d, *J* = 8.8 Hz, 2H), 7.85 (t, *J* = 8.1 Hz, 2H), 7.69 (s, 1H), 7.56 (d, *J* = 8.2 Hz, 1H), 7.50 (d, *J* = 8.1 Hz, 1H), 7.37 (t, *J* = 7.6 Hz, 2H), 5.87 (s, 2H), 3.78 (t, *J* = 6.8 Hz, 2H), 2.42 (s, 3H), 1.03 (q, *J* = 7.2 Hz, 2H), 0.90 (q, *J* = 7.5 Hz, 2H), 0.58 (t, *J* = 7.3 Hz, 3H). ^{13}C NMR (151 MHz, DMSO-d_6) *δ* 177.16, 165.83, 143.09, 142.35, 140.63, 134.59, 133.62, 133.16, 131.20, 127.76, 127.12, 126.67, 125.49, 122.26, 122.00, 116.93, 65.15, 41.84, 29.98, 20.91, 18.64, 13.68.

ethyl 4-(4-((9-oxoacridin-10(9H)-yl)methyl)-1H-1,2,3-triazol-1-yl)benzoate (L14)

^{1}H NMR (600 MHz, DMSO-d_6) δ 1.32 (t, *J* = 7.06 Hz, 3 H), 4.32 (q, *J* = 7.15 Hz, 2 H), 5.91 (s, 2 H), 7.36 (t, *J* = 7.34 Hz, 2 H), 7.78 - 7.86 (m, 2 H), 7.94 (d, *J* = 8.66 Hz, 2 H), 8.01 - 8.06 (m, *J* = 8.66 Hz, 2 H), 8.06 - 8.12 (m, *J* = 8.85 Hz, 2 H), 8.37 (dd, *J* = 8.00, 1.41 Hz, 2 H), 8.94 (s, 1 H); ^{13}C NMR (151 MHz, DMSO-d_6) δ 14.5, 42.2, 61.5, 116.7, 120.3, 122.0, 122.1, 122.2, 127.0, 130.0, 131.2, 134.7, 139.9, 142.3, 144.7, 165.2, 177.1.

4-(4-((9-oxoacridin-10(9H)-yl)methyl)-1H-1,2,3-triazol-1-yl)benzoic acid (L15)

^{1}H NMR (600 MHz, DMSO-d_6) δ 5.90 (s, 2 H), 7.35 (t, *J* = 7.43 Hz, 2 H), 7.82 (td, *J* = 7.86, 1.60 Hz, 2 H), 7.95 (d, *J* = 8.66 Hz, 2 H), 7.98 - 8.03 (m, *J* = 8.85 Hz, 2 H), 8.04 - 8.11 (m, *J* = 8.66 Hz, 2 H), 8.37 (dd, *J* = 7.91, 1.51 Hz, 2 H), 8.98 (s, 1 H), 13.24 (br. s., 1 H); ^{13}C NMR (151 MHz, DMSO-d_6) δ 42.2, 116.7, 120.1, 121.9, 122.2, 127.0, 131.1, 131.4, 134.7, 139.7, 142.3, 144.6, 166.7, 177.1.

methyl 4-(4-((9-oxoacridin-10(9H)-yl)methyl)-1H-1,2,3-triazol-1-yl)benzoate (L16)

^{1}H NMR (600 MHz, DMSO-d_6) δ 3.86 (s, 3 H), 5.91 (s, 2 H), 7.36 (t, *J* = 7.34 Hz, 2 H), 7.82 (td, *J* = 7.81, 1.51 Hz, 2 H), 7.94 (d, *J* = 8.85 Hz, 2 H), 8.04 (d, *J* = 8.66 Hz, 2 H), 8.10 (d, *J* = 8.66 Hz, 2 H), 8.37 (dd, *J* = 7.91, 1.51 Hz, 2 H), 8.94 (s, 1 H); ^{13}C NMR (151 MHz, DMSO-d_6) δ 42.2, 52.8, 116.7, 120.3, 122.0, 122.1, 122.2, 127.0, 129.8, 131.3, 134.7, 140.0, 142.3, 144.7, 165.7, 177.1.

butyl 4-(4-((9-oxoacridin-10(9H)-yl)methyl)-1H-1,2,3-triazol-1-yl)benzoate (L17)

^{1}H NMR (600 MHz, DMSO-*d6*) δ 0.92 (t, J = 7.43 Hz, 3 H), 1.41 (dq, J = 14.94, 7.44 Hz, 2 H), 1.64 - 1.72 (m, 2 H), 4.28 (t, J = 6.49 Hz, 2 H), 5.90 (s, 2 H), 7.36 (t, J = 7.34 Hz, 2 H), 7.78 - 7.85 (m, 2 H), 7.94 (d, J = 8.66 Hz, 2 H), 8.00 - 8.05 (m, J = 8.66 Hz, 2 H), 8.06 - 8.13 (m, J = 8.66 Hz, 2 H), 8.37 (dd, J = 7.91, 1.32 Hz, 2 H), 8.94 (s, 1 H); ^{13}C NMR (151 MHz, DMSO-*d6*) δ 14.0, 19.1, 30.6, 42.2, 65.1, 116.7, 120.3, 121.9, 122.1, 122.2, 127.0, 130.0, 131.2, 134.7, 140.0, 142.3, 144.7, 165.2, 177.1.

ethyl 2-(4-((9-oxoacridin-10(9H)-yl)methyl)-1H-1,2,3-triazol-1-yl)benzoate (L18)

^{1}H NMR (600 MHz, DMSO-d_6) δ 0.68 (t, *J* = 7.15 Hz, 3 H), 3.84 (q, *J* = 7.03 Hz, 2 H), 5.90 (s, 2 H), 7.36 (t, *J* = 7.43 Hz, 2 H), 7.61 (d, *J* = 7.91 Hz, 1 H), 7.67 (t, *J* = 7.53 Hz, 1 H), 7.76 (t, *J* = 7.62 Hz, 1 H), 7.81 - 7.90 (m, 3 H), 8.05 (d, *J* = 8.85 Hz, 2 H), 8.37 (d, *J* = 7.15 Hz, 2 H), 8.67 (s, 1 H); ^{13}C NMR (151 MHz, DMSO-d_6) δ 13.6, 41.6, 61.4, 116.8, 121.9, 122.1, 125.4, 126.7, 127.0, 128.0, 130.5, 130.9, 133.2, 134.5, 135.4, 142.2, 143.1, 165.6, 177.0.

2-(4-((9-oxoacridin-10(9H)-yl)methyl)-1H-1,2,3-triazol-1-yl)benzoic acid (L19)

^{1}H NMR (600 MHz, DMSO-d_6) δ 5.87 (s, 2 H), 7.33 - 7.39 (m, 2 H), 7.55 (dd, *J* = 7.91, 1.13 Hz, 1 H), 7.65 (td, *J* = 7.58, 1.22 Hz, 1 H), 7.69 - 7.73 (m, 1 H), 7.83 (ddd, *J* = 8.71, 6.92, 1.69 Hz, 2 H), 7.90 (dd, *J* = 7.62, 1.41 Hz, 1 H), 8.01 (d, *J* = 8.66 Hz, 2 H), 8.36 (dd, *J* = 8.09, 1.69 Hz, 2 H), 8.64 (s, 1 H), 13.12 (br. s., 1 H); ^{13}C NMR (151 MHz, DMSO-d_6) δ 42.0, 116.9, 121.9, 122.1, 125.6, 127.0, 128.8, 130.4, 130.8, 132.8, 134.6, 135.6, 142.3, 142.9, 166.8, 177.1

methyl 2-(4-((9-oxoacridin-10(9H)-yl)methyl)-1H-1,2,3-triazol-1-yl)benzoate (L20)

^{1}H NMR (600 MHz, DMSO-d_6) δ 3.42 (s, 3 H), 5.90 (s, 2 H), 7.36 (t, *J* = 7.43 Hz, 2 H), 7.62 (d, *J* = 7.91 Hz, 1 H), 7.67 (t, *J* = 7.43 Hz, 1 H), 7.75 (t, *J* = 7.43 Hz, 1 H), 7.84 (t, *J* = 7.34 Hz, 2 H), 7.89 (d, *J* = 7.34 Hz, 1 H), 8.00 (d, *J* = 8.85 Hz, 2 H), 8.37 (dt, *J* = 7.95, 0.92 Hz, 2 H), 8.62 (s, 1 H); ^{13}C NMR (151 MHz, DMSO-d_6) δ 41.9, 52.6, 116.8, 121.9, 122.2, 125.4, 126.8, 127.0, 127.4, 130.4, 130.9, 133.3, 134.6, 135.5, 142.3, 143.2, 165.9, 177.1

butyl 2-(4-((9-oxoacridin-10(9H)-yl)methyl)-1H-1,2,3-triazol-1-yl)benzoate (L21)

^{1}H NMR (600 MHz, DMSO-d_6) δ 0.57 (t, *J* = 7.43 Hz, 3 H), 0.89 (dq, *J* = 14.92, 7.39 Hz, 2 H), 0.99 - 1.08 (m, 2 H), 3.78 (t, *J* = 6.78 Hz, 2 H), 5.87 (s, 2 H), 7.36 (t, *J* = 7.25 Hz, 2 H), 7.60 - 7.63 (m, 1 H), 7.67 (td, *J* = 7.62, 1.13 Hz, 1 H), 7.74 - 7.78 (m, 1 H), 7.84 (ddd, *J* = 8.71, 6.92, 1.69 Hz, 2 H), 7.87 (dd, *J* = 7.72, 1.32 Hz, 1 H), 8.06 (d, *J* = 8.85 Hz, 2 H), 8.37 (dd, *J* = 8.00, 1.60 Hz, 2 H), 8.72 (s, 1 H); ^{13}C NMR (151 MHz, DMSO-d_6) δ 13.6, 18.5, 29.9, 41.7, 65.1, 116.8, 121.9, 122.2, 125.4, 126.7, 127.0, 127.9, 130.5, 130.9, 133.2, 134.5, 135.3, 142.2, 143.1, 165.7, 177.0.

10-((2-phenyloxazol-4-yl)methyl)acridin-9(10H)-one (N1)

^{1}H NMR (600 MHz, DMSO-d_6) δ 8.39 (d, J = 7.9 Hz, 2H), 8.19 (s, 1H), 7.93 (t, J = 7.6 Hz, 4H), 7.83 (t, J = 7.8 Hz, 2H), 7.51 (d, J = 5.3 Hz, 3H), 7.37 (t, J = 7.4 Hz, 2H), 5.73 (s, 2H). ^{13}C NMR (151 MHz, DMSO-d_6) δ 177.24, 161.57, 142.43(2C), 138.03(2C), 137.90, 134.64(2C), 131.28, 129.64(2C), 127.08(2C), 126.99, 126.41(2C), 122.25, 122.03(2C), 116.84(2C), 43.12.

10-((2-(p-tolyl)oxazol-4-yl)methyl)acridin-9(10H)-one (N2)

^{1}H NMR (600 MHz, DMSO-d_6) δ 8.37 (dd, J = 8.0, 1.7 Hz, 2H), 8.14 (d, J = 1.1 Hz, 1H), 7.93 (d, J = 8.7 Hz, 2H), 7.85 – 7.80 (m, 3H), 7.80 (d, J = 1.9 Hz, 1H), 7.36 (ddd, J = 7.9, 6.8, 0.8 Hz, 2H), 7.31 (d, J = 8.0 Hz, 2H), 5.71 (d, J = 1.3 Hz, 2H), 2.35 (s, 3H). ^{13}C NMR (151 MHz, DMSO-d_6) δ 177.21, 161.75, 142.46(2C), 141.22(2C), 137.90, 137.56(2C), 134.64, 130.21(2C), 127.07(2C), 126.41, 124.40(2C), 122.26, 122.03(2C), 116.89(2C), 43.17, 21.49.

10-((2-(4-fluorophenyl)oxazol-4-yl)methyl)acridin-9(10H)-one (N3)

^{1}H NMR (600 MHz, DMSO-d_6) δ 8.38 (dd, J = 8.0, 1.7 Hz, 2H), 8.19 (d, J = 1.2 Hz, 1H), 7.97 – 7.93 (m, 3H), 7.92 (s, 1H), 7.83 (ddd, J = 8.8, 6.9, 1.7 Hz, 2H), 7.39 – 7.36 (m, 2H), 7.36 – 7.33 (m, 2H), 5.72 (d, J = 1.3 Hz, 2H). ^{13}C NMR (151 MHz, DMSO-d_6) δ 177.21,(164.76, 163.12), 160.79, 142.44(2C), 138.04, 138.01, 134.63(2C), 128.99, 128.93, 127.08(2C), (123.71, 123.69), 122.26(2C), 122.02(2C), 116.89, 116.86(2C), 116.74, 43.06.

10-((2-(4-chlorophenyl)oxazol-4-yl)methyl)acridin-9(10H)-one (N4)

^{1}H NMR (600 MHz, DMSO-d_6) δ 8.38 (dd, J = 8.0, 1.8 Hz, 2H), 8.22 (d, J = 1.3 Hz, 1H), 7.93 (d, J = 8.8 Hz, 2H), 7.91 (d, J = 8.7 Hz, 2H), 7.84 (dd, J = 6.9, 1.8 Hz, 2H), 7.58 (d, J = 8.7 Hz, 2H), 7.37 (ddd, J = 7.8, 6.8, 0.8 Hz, 2H), 5.72 (d, J = 1.3 Hz, 2H). ^{13}C NMR (151 MHz, DMSO-d_6) δ 177.22, 142.44(2C), 138.27, 138.22, 135.98(2C), 134.66(2C), 129.83(2C), 128.20(2C), 127.08(2C), 125.82, 122.25, 122.06(2C), 116.87(2C), 43.01.

10-((2-(4-bromophenyl)oxazol-4-yl)methyl)acridin-9(10H)-one (N5)

^{1}H NMR (600 MHz, DMSO-d_6) δ 8.37 (dd, J = 8.0, 1.8 Hz, 2H), 8.23 (s, 1H), 7.93 (d, J = 8.8 Hz, 2H), 7.83 (ddd, J = 8.7, 5.5, 1.9 Hz, 4H), 7.74 – 7.69 (m, 2H), 7.40 – 7.33 (m, 2H), 5.85 – 5.63 (m, 2H). ^{13}C NMR (151 MHz, DMSO-d_6) δ 177.23, 160.77, 142.43(2C), 138.29, 138.24, 134.67(2C), 132.75(2C), 132.72(2C), 128.36(2C), 127.08(2C), 126.15, 124.82, 122.24, 122.06, 116.86(2C), 43.01.

10-((2-(4-methoxyphenyl)oxazol-4-yl)methyl)acridin-9(10H)-one (N6)

^{1}H NMR (600 MHz, DMSO-d_6) δ 8.38 (dd, J = 8.0, 1.7 Hz, 2H), 8.09 (d, J = 1.2 Hz, 1H), 7.92 (d, J = 8.7 Hz, 2H), 7.87 – 7.81 (m, 4H), 7.36 (ddd, J = 7.9, 6.9, 0.9 Hz, 2H), 7.09 – 7.00 (m, 2H), 5.69 (d, J = 1.3 Hz, 2H), 3.81 (s, 3H). ^{13}C NMR (151 MHz, DMSO-d_6) δ 177.21, 161.69, 161.66, 142.45(2C), 137.77, 137.18(2C), 134.61(2C), 128.19(2C), 127.07(2C), 122.26, 122.00(2C), 119.69, 116.88(2C), 115.05(2C), 55.83, 43.21.

10-((2-(2-fluorophenyl)oxazol-4-yl)methyl)acridin-9(10H)-one (N7)

^{1}H NMR (600 MHz, DMSO-d_6) δ 8.37 (dd, J = 8.0, 1.8 Hz, 2H), 8.24 (s, 1H), 7.95 – 7.88 (m, 3H), 7.82 (ddd, J = 8.7, 6.9, 1.8 Hz, 2H), 7.56 (dddd, J = 8.5, 7.0, 5.0, 1.8 Hz, 1H), 7.42 – 7.26 (m, 4H), 5.73 (d, J = 1.2 Hz, 2H). ^{13}C NMR (151 MHz, DMSO-d_6) δ 177.23, (160.43, 158.73), (158.04, 158.01), 142.43(2C), 138.30, 138.01, 134.64(2C), (133.44, 133.38), 129.88, 127.08(2C), (125.54, 125.52), 122.26(2C), 122.03(2C), (117.55, 117.41), 116.84(2C), (115.14, 115.07), 43.11.

10-((2-(o-tolyl)oxazol-4-yl)methyl)acridin-9(10H)-one (N8)

^{1}H NMR (600 MHz, DMSO-d_6) δ 8.37 (dd, J = 8.0, 1.8 Hz, 2H), 8.25 (s, 1H), 8.00 (d, J = 8.7 Hz, 2H), 7.88 – 7.80 (m, 3H), 7.42 – 7.28 (m, 5H), 5.74 (s, 2H), 2.53 (s, 3H). ^{13}C NMR (151 MHz, DMSO-d_6) δ 177.20, 161.67, 142.48(2C), 137.54, 137.42, 137.29, 134.55(2C), 132.21(2C), 130.83, 128.69, 127.05(2C), 126.78, 125.96, 122.24, 122.01(2C), 117.04(2C), 42.95, 21.90.

The NMR spectrum of our compounds.

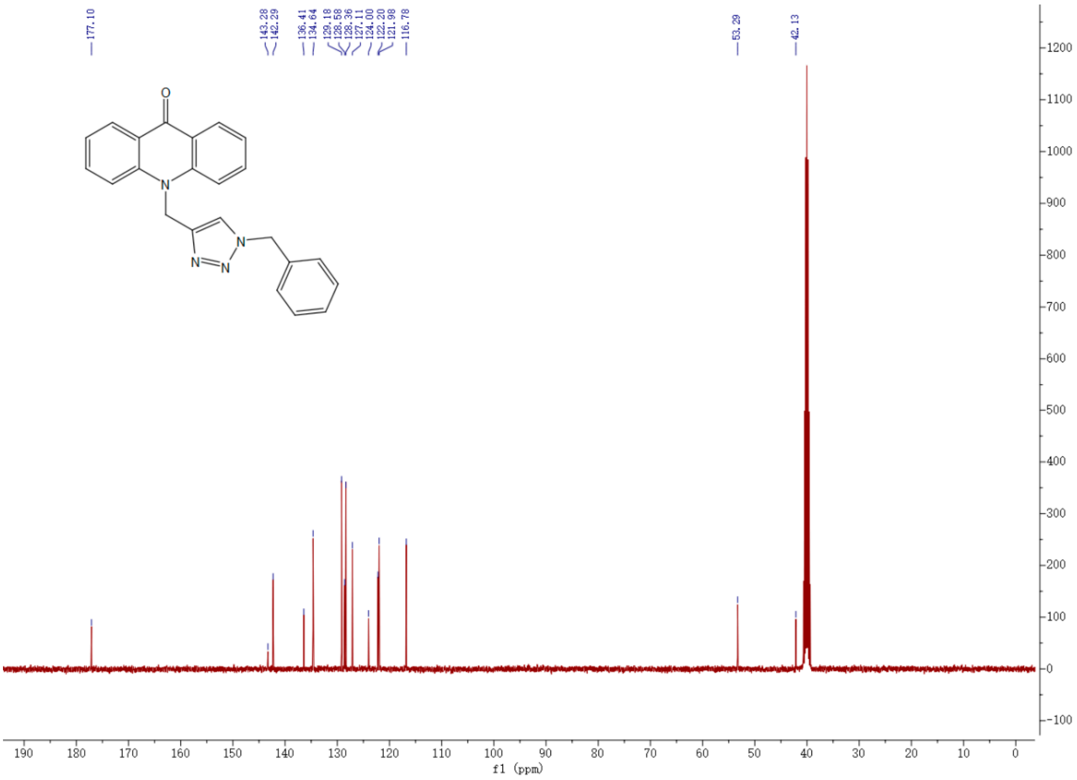

Figure S1. ^{13}C NMR of compound **L1**.

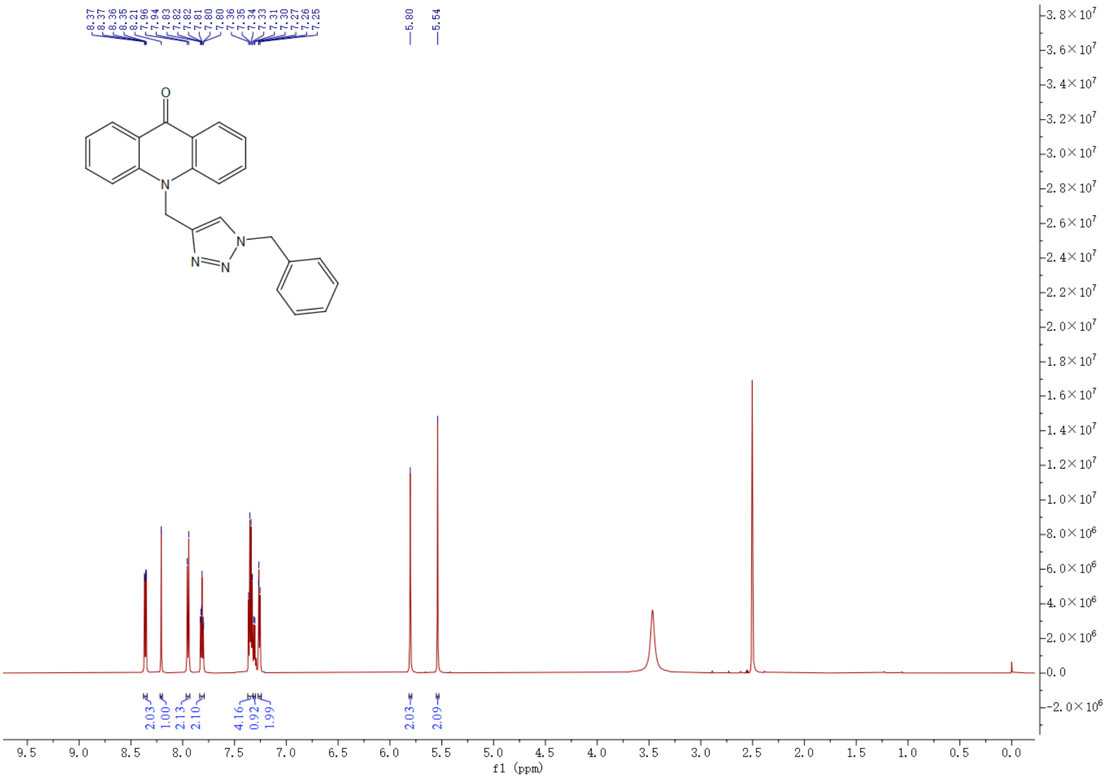

Figure S2. ^{1}H NMR of compound **L1**.

This report was created by ACD/NMR Processor Academic Edition. For more information go to www.acdlabs.com/nmrproc/

2015/10/27 16:44:04

Acquisition Time (sec)	0.8389	*Comment*	AVANCE-13C IN DMSO BY BBO Sample:L4			*Date*	26 Oct 2015 10:50:40
Date Stamp	26 Oct 2015 10:50:40			*File Name*	C:\Users\yaohua01\AppData\Local\Temp\RaRa0.088\L4 C\2\PDATA\1\1r		
Frequency (MHz)	150.90	*Nucleus*	13C	*Number of Transients*	2278	*Origin*	spect
Original Points Count	32768	*Owner*	nmr	*Points Count*	32768	*Pulse Sequence*	zgpg30
Receiver Gain	174.88	*SW(cyclical) (Hz)*	39062.50	*Solvent*	DMSO-d6	*Spectrum Offset (Hz)*	16585.1211
Spectrum Type	STANDARD	*Sweep Width (Hz)*	39061.31	*Temperature (degree C)*	24.897		

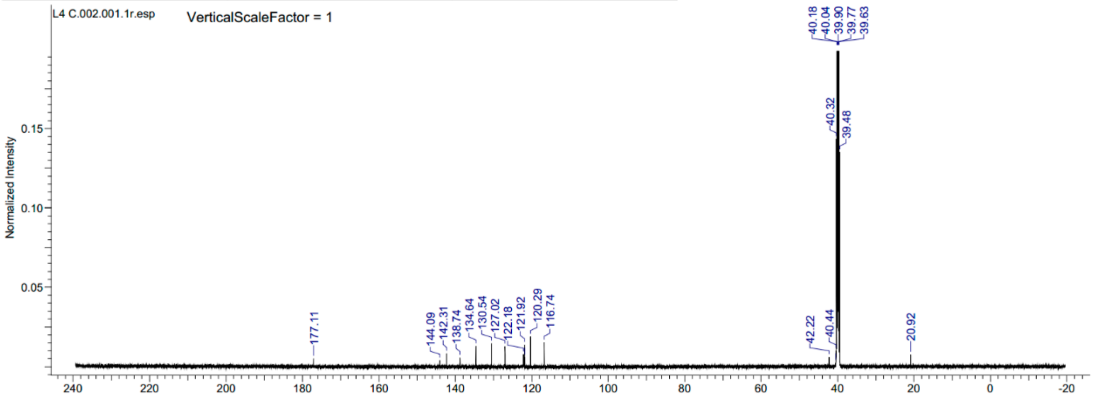

Figure S3. ^{13}C NMR of compound **L2**

This report was created by ACD/NMR Processor Academic Edition. For more information go to www.acdlabs.com/nmrproc/

2015/10/26 18:17:04

Acquisition Time (sec)	2.6564	*Comment*	AVANCE-1H IN DMSO By BBO Sample:DJ-L-04			*Date*	29 May 2014 12:56:32
Date Stamp	29 May 2014 12:56:32			*File Name*	C:\Users\yaohua01\AppData\Local\Temp\RaRa0.396\DJ-L-04\1\PDATA\1\1r		
Frequency (MHz)	600.13	*Nucleus*	1H	*Number of Transients*	32	*Origin*	spect
Original Points Count	32768	*Owner*	nmr	*Points Count*	65536	*Pulse Sequence*	zg30
Receiver Gain	174.88	*SW(cyclical) (Hz)*	12335.53	*Solvent*	DMSO-d6	*Spectrum Offset (Hz)*	3694.9656
Spectrum Type	STANDARD	*Sweep Width (Hz)*	12335.34	*Temperature (degree C)*	24.884		

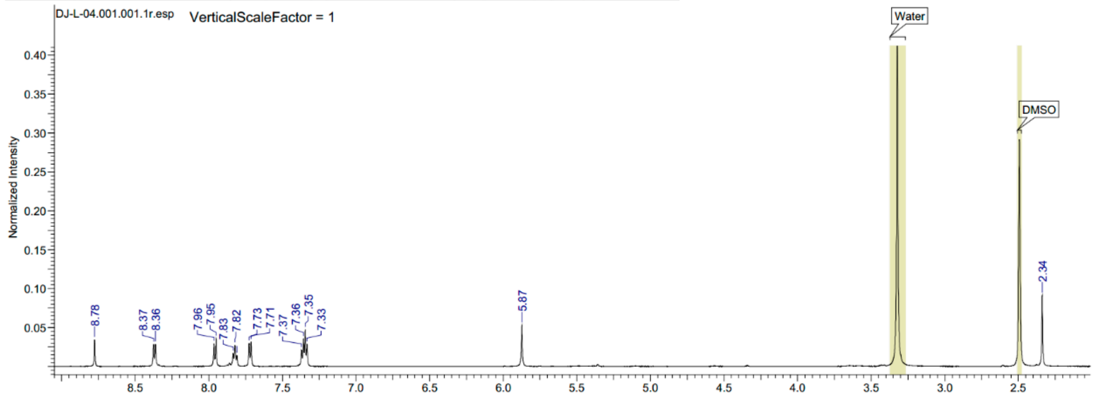

Figure S4. ^{1}H NMR of compound **L2**.

13-C.pdf

This report was created by ACD/NMR Processor Academic Edition. For more information go to www.acdlabs.com/nmrproc/

2015/10/27 15:27:45

Acquisition Time (sec)	0.8389	*Comment*	AVANCE-13C IN DMSO BY BBO Sample:L5			*Date*	26 Oct 2015 14:26:08
Date Stamp	26 Oct 2015 14:26:08			*File Name*	C:\Users\yaohua01\AppData\Local\Temp\RaRa0.885\L5 C\2\PDATA\1\1r		
Frequency (MHz)	150.90	*Nucleus*	13C	*Number of Transients*	1034	*Origin*	spect
Original Points Count	32768	*Owner*	nmr	*Points Count*	32768	*Pulse Sequence*	zgpg30
Receiver Gain	174.88	*SW(cyclical) (Hz)*	39062.50	*Solvent*	DMSO-d6	*Spectrum Offset (Hz)*	16585.1211
Spectrum Type	STANDARD	*Sweep Width (Hz)*	39061.31	*Temperature (degree C)*	24.887		

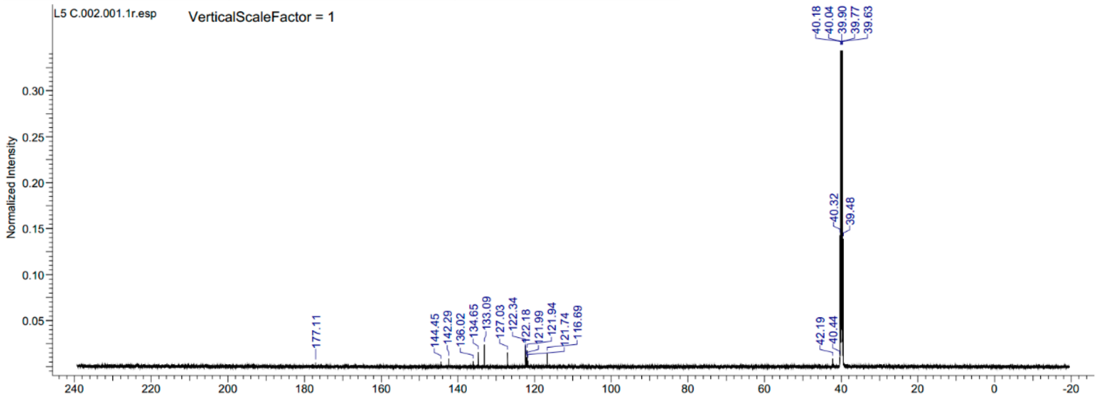

Figure S5. ^{13}C NMR of compound **L3**.

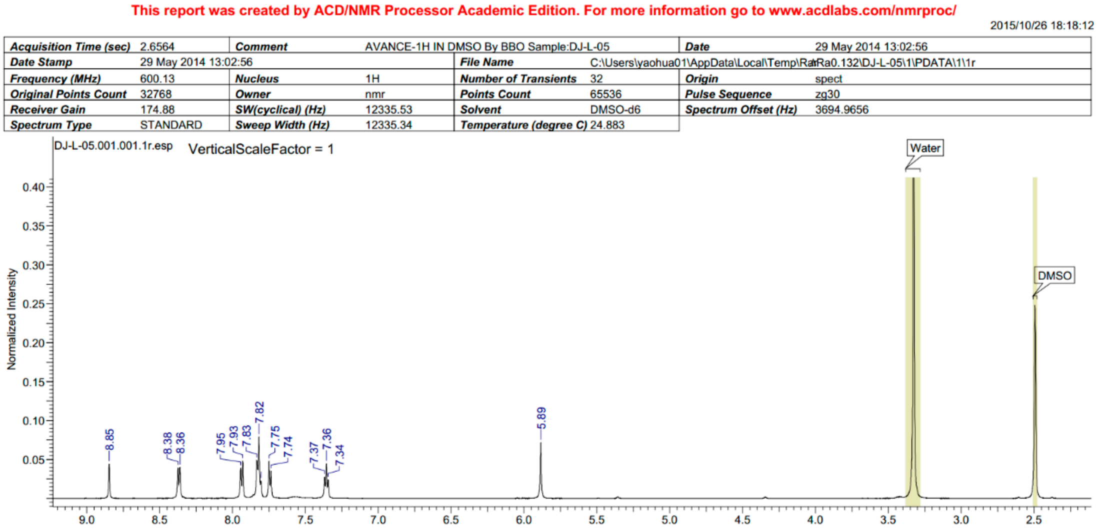

Figure S6. ^{1}H NMR of compound **L3**.

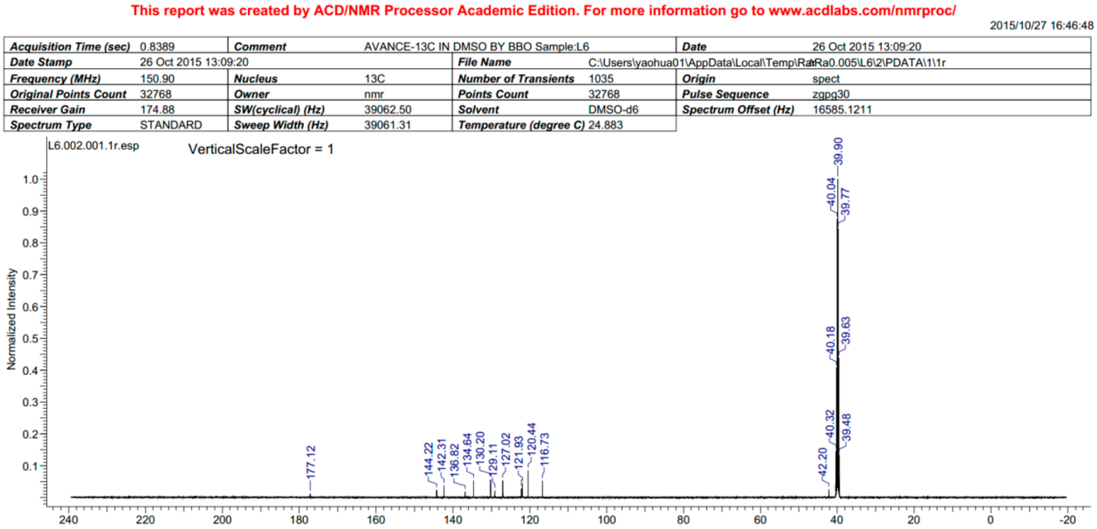

Figure S7. ^{13}C NMR of compound **L4**.

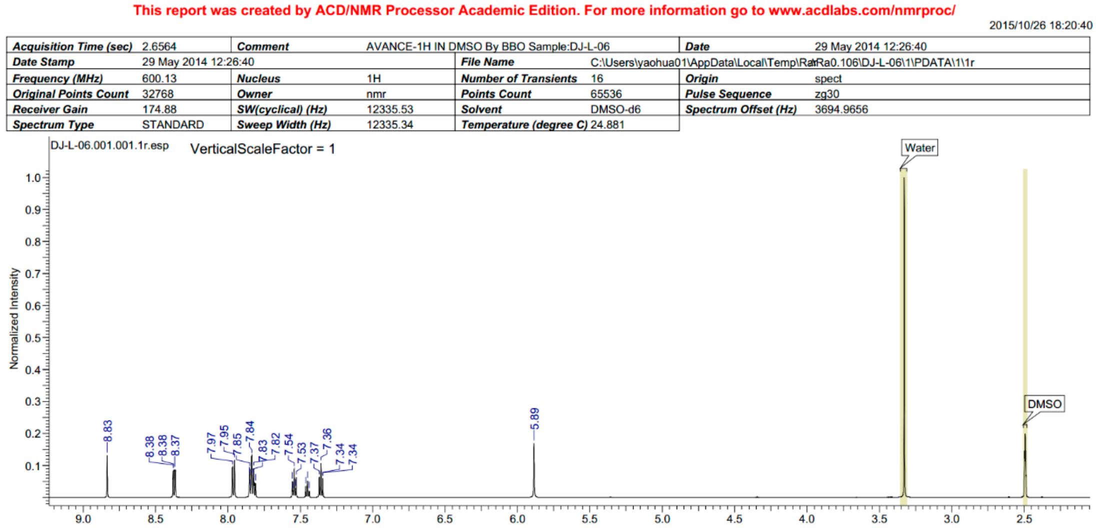

Figure S8. ^{1}H NMR of compound **L4**.

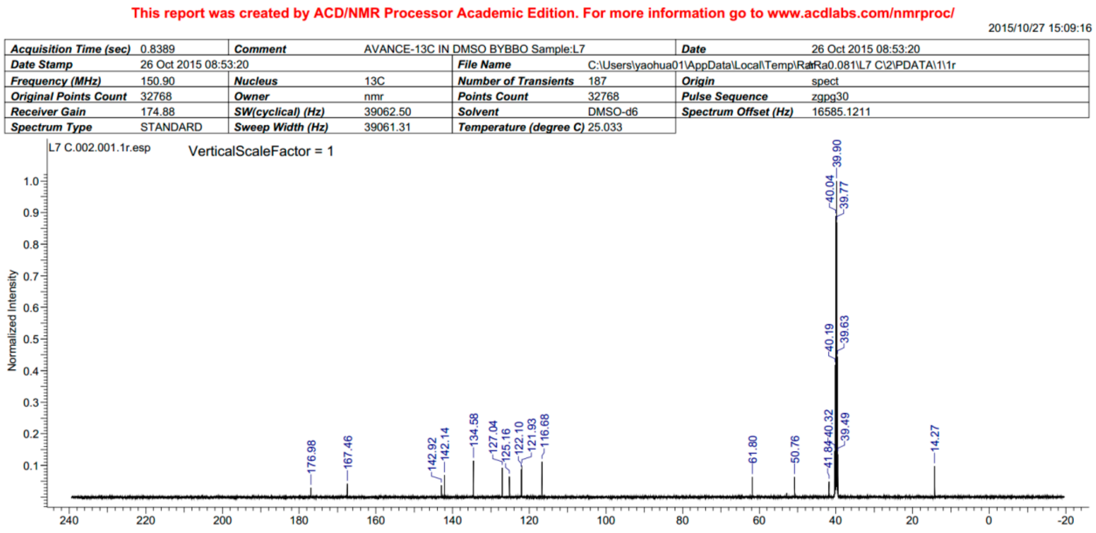

Figure S9. ^{13}C NMR of compound **L5**.

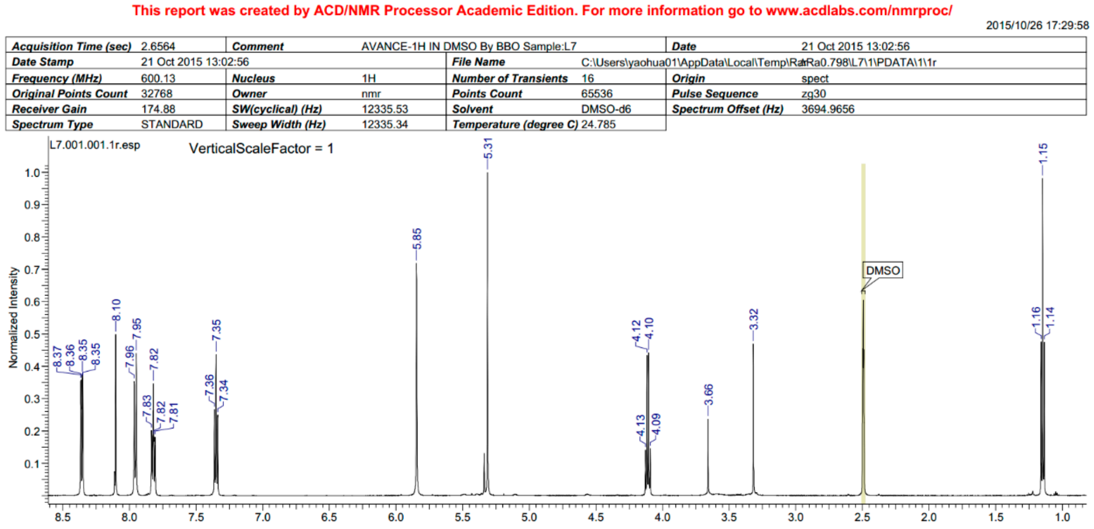

Figure S10. ^{1}H NMR of compound **L5**.

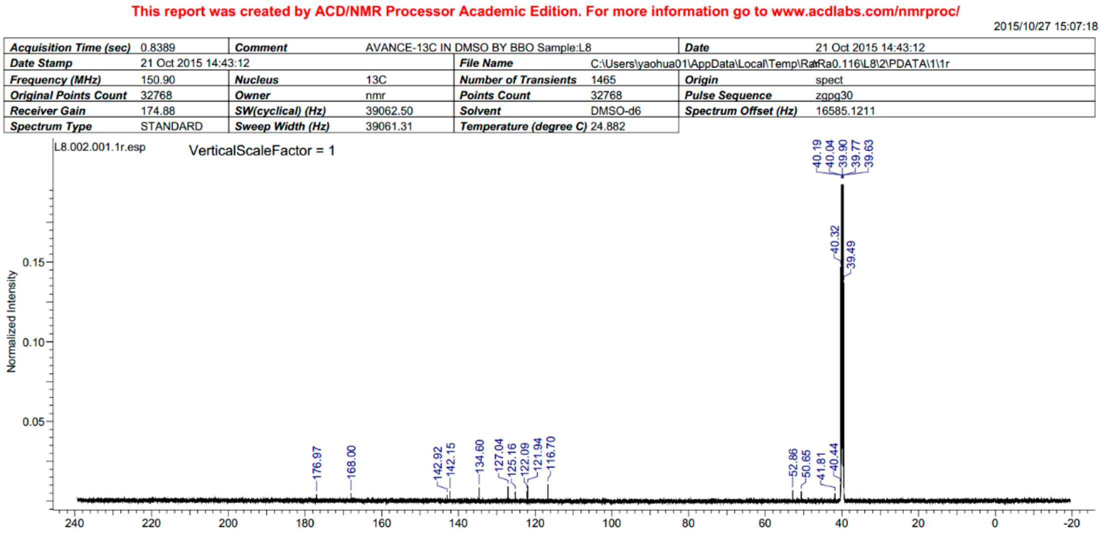

Figure S11. ^{13}C NMR of compound **L6**.

This report was created by ACD/NMR Processor Academic Edition. For more information go to www.acdlabs.com/nmrproc/

2015/10/26 18:23:14

**Acquisition Time (sec)**	2.6564	**Comment**	AVANCE-1H IN DMSO By BBO Sample:DJ-L-08	**Date**	29 May 2014 12:45:52
**Date Stamp**	29 May 2014 12:45:52			**File Name**	C:\Users\yaohua01\AppData\Local\Temp\Rar$DRa0.746\DJ-L-08\1\PDATA\1\1r
**Frequency (MHz)**	600.13	**Nucleus**	1H	**Number of Transients**	16
**Origin**	spect				
**Original Points Count**	32768	**Owner**	nmr	**Points Count**	65536
**Pulse Sequence**	zg30				
**Receiver Gain**	174.88	**SW(cyclical) (Hz)**	12335.53	**Solvent**	DMSO-d6
**Spectrum Offset (Hz)**	3694.9656				
**Spectrum Type**	STANDARD	**Sweep Width (Hz)**	12335.34	**Temperature (degree C)**	24.881

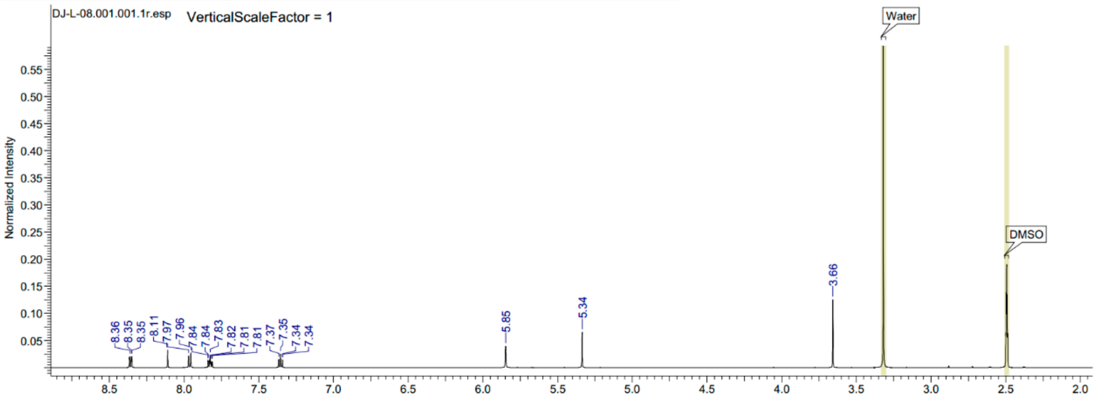

Figure S12. ^{1}H NMR of compound **L6**.

This report was created by ACD/NMR Processor Academic Edition. For more information go to www.acdlabs.com/nmrproc/

2015/10/27 16:49:55

**Acquisition Time (sec)**	0.8389	**Comment**	AVANCE-13C IN DMSO BY BBO Sample:L9	**Date**	26 Oct 2015 09:40:16
**Date Stamp**	26 Oct 2015 09:40:16			**File Name**	C:\Users\yaohua01\AppData\Local\Temp\Rar$DRa0.187\L9 C\2\PDATA\1\1r
**Frequency (MHz)**	150.90	**Nucleus**	13C	**Number of Transients**	192
**Origin**	spect				
**Original Points Count**	32768	**Owner**	nmr	**Points Count**	32768
**Pulse Sequence**	zgpg30				
**Receiver Gain**	174.88	**SW(cyclical) (Hz)**	39062.50	**Solvent**	DMSO-d6
**Spectrum Offset (Hz)**	16585.1211				
**Spectrum Type**	STANDARD	**Sweep Width (Hz)**	39061.31	**Temperature (degree C)**	24.903

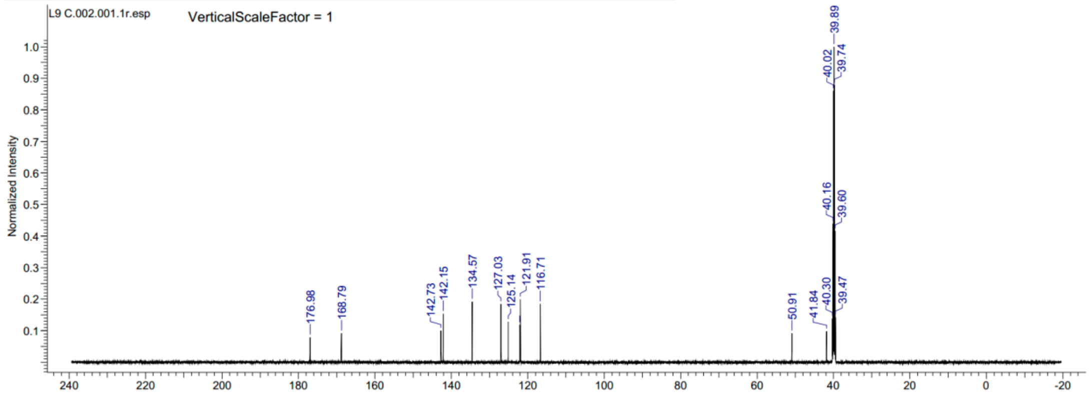

Figure S13. ^{13}C NMR of compound **L7**

This report was created by ACD/NMR Processor Academic Edition. For more information go to www.acdlabs.com/nmrproc/

2015/10/26 17:32:24

**Acquisition Time (sec)**	2.6564	**Comment**	AVANCE-1H IN DMSO By BBO Sample:L9	**Date**	21 Oct 2015 12:26:40
**Date Stamp**	21 Oct 2015 12:26:40			**File Name**	C:\Users\yaohua01\AppData\Local\Temp\Rar$DRa0.101\L9\1\PDATA\1\1r
**Frequency (MHz)**	600.13	**Nucleus**	1H	**Number of Transients**	16
**Origin**	spect				
**Original Points Count**	32768	**Owner**	nmr	**Points Count**	65536
**Pulse Sequence**	zg30				
**Receiver Gain**	174.88	**SW(cyclical) (Hz)**	12335.53	**Solvent**	DMSO-d6
**Spectrum Offset (Hz)**	3694.9656				
**Spectrum Type**	STANDARD	**Sweep Width (Hz)**	12335.34	**Temperature (degree C)**	24.880

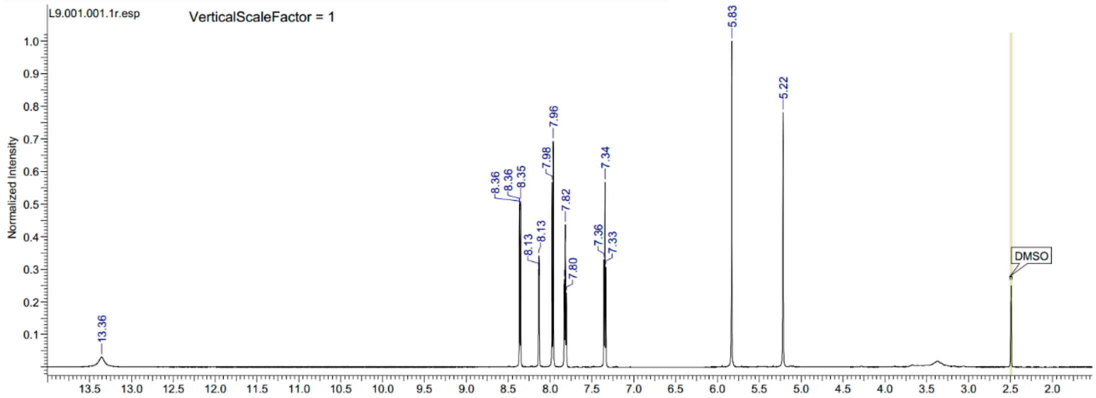

Figure S14. ^{1}H NMR of compound **L7**.

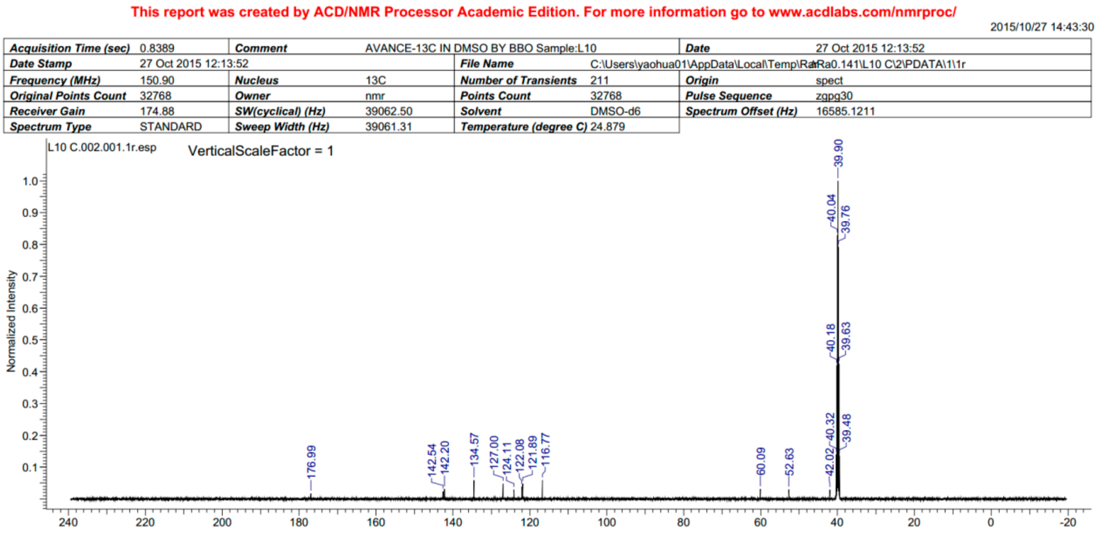

Figure S15. ^{13}C NMR of compound **L8**.

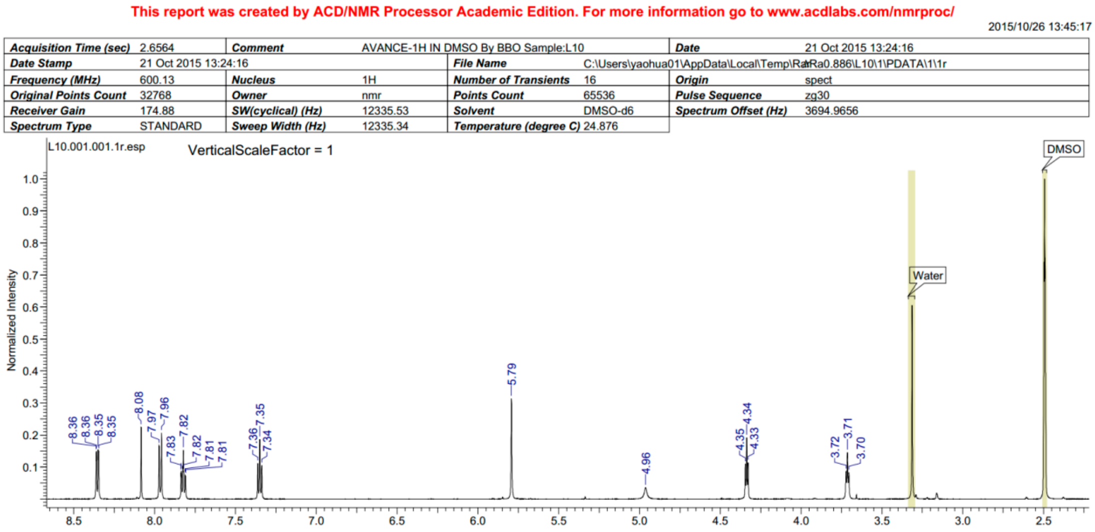

Figure S16. ^{1}H NMR of compound **L8**.

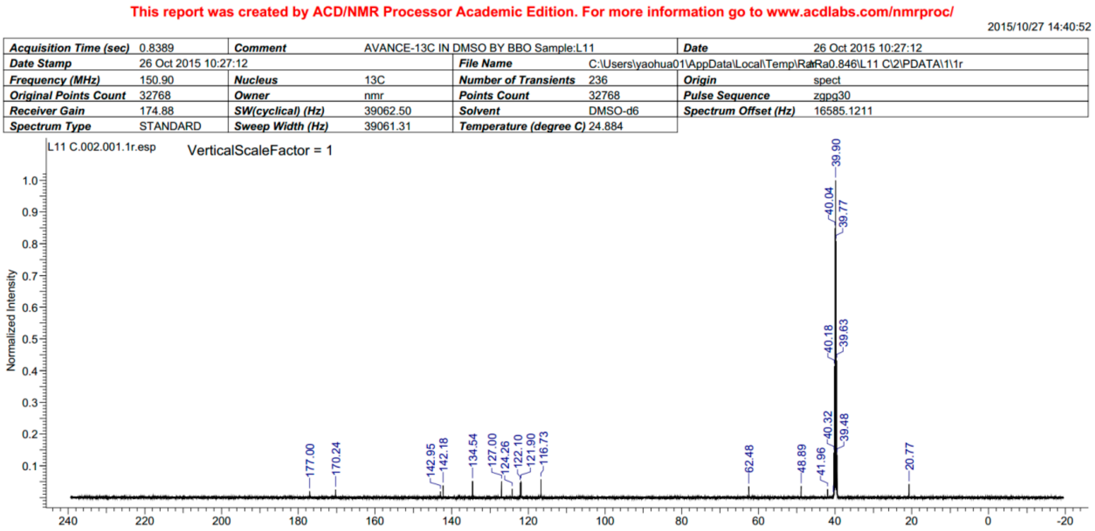

Figure S17. ^{13}C NMR of compound **L9**.

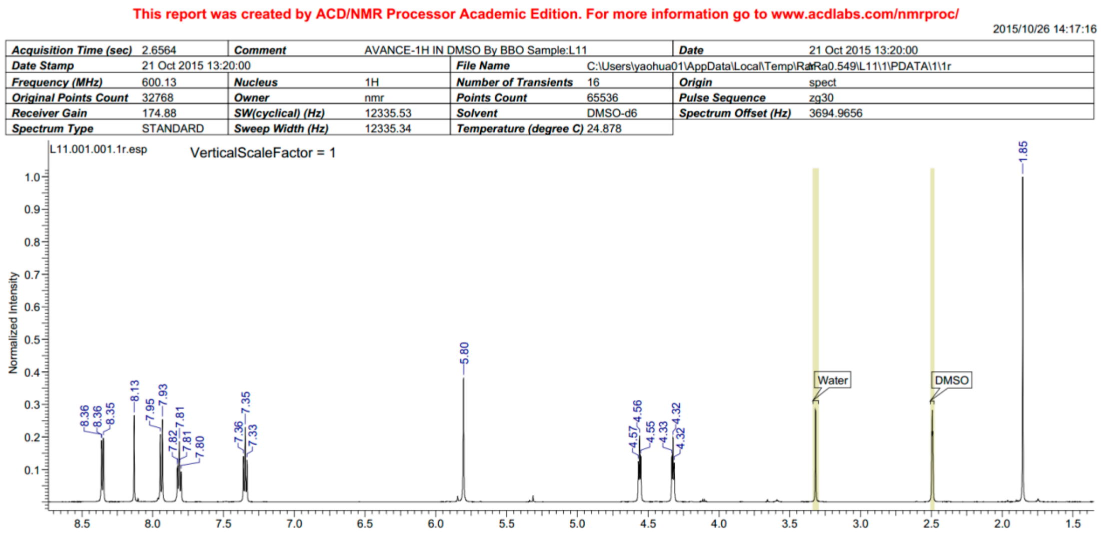

Figure S18. ^{1}H NMR of compound **L9**.

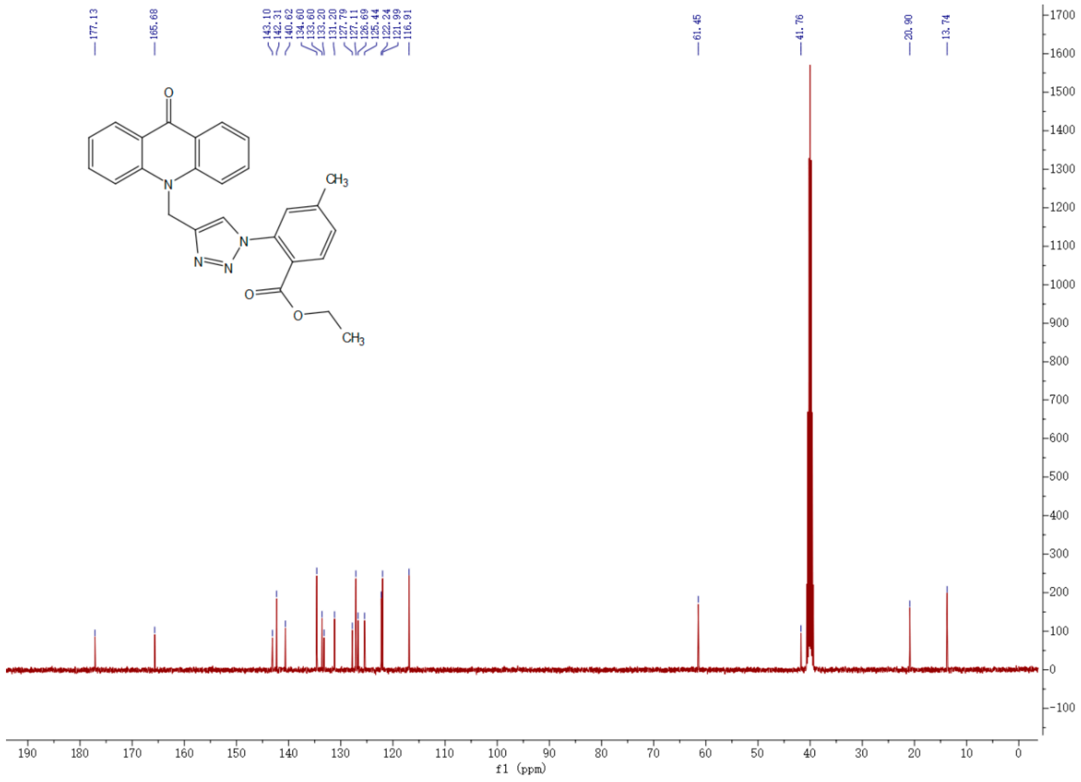

Figure S19. ^{13}C NMR of compound **L10**.

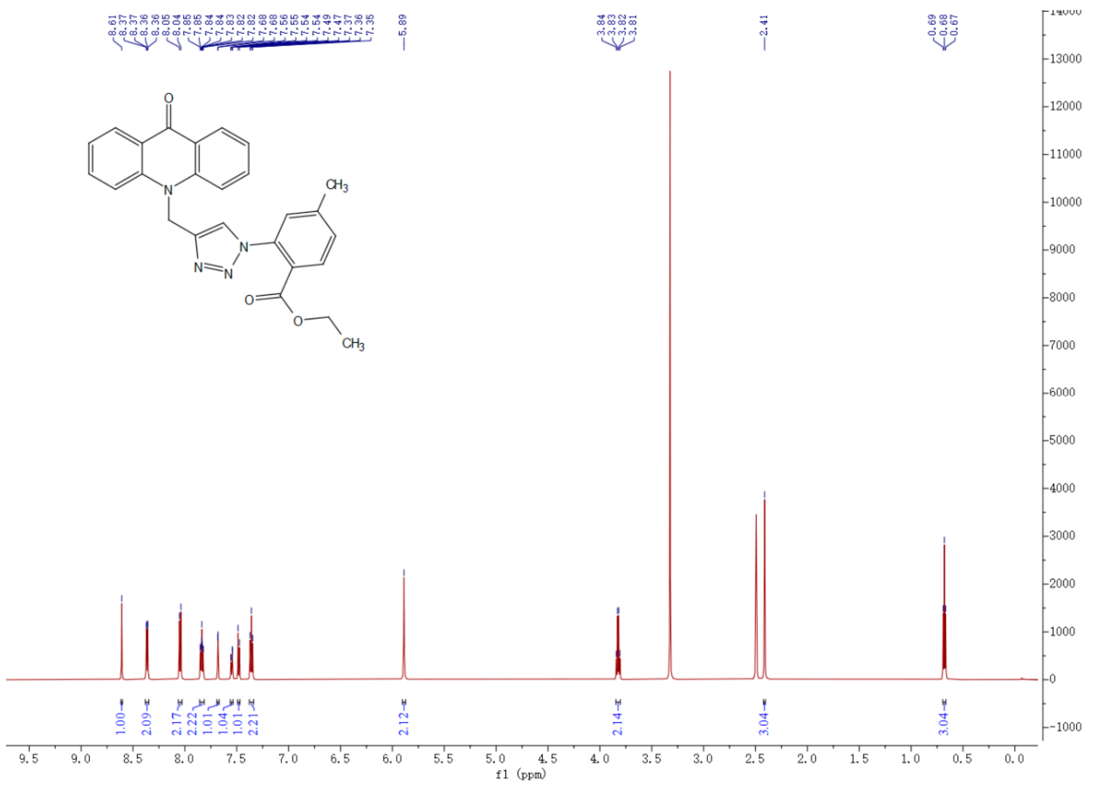

Figure S20. ^{1}H NMR of compound **L10**.

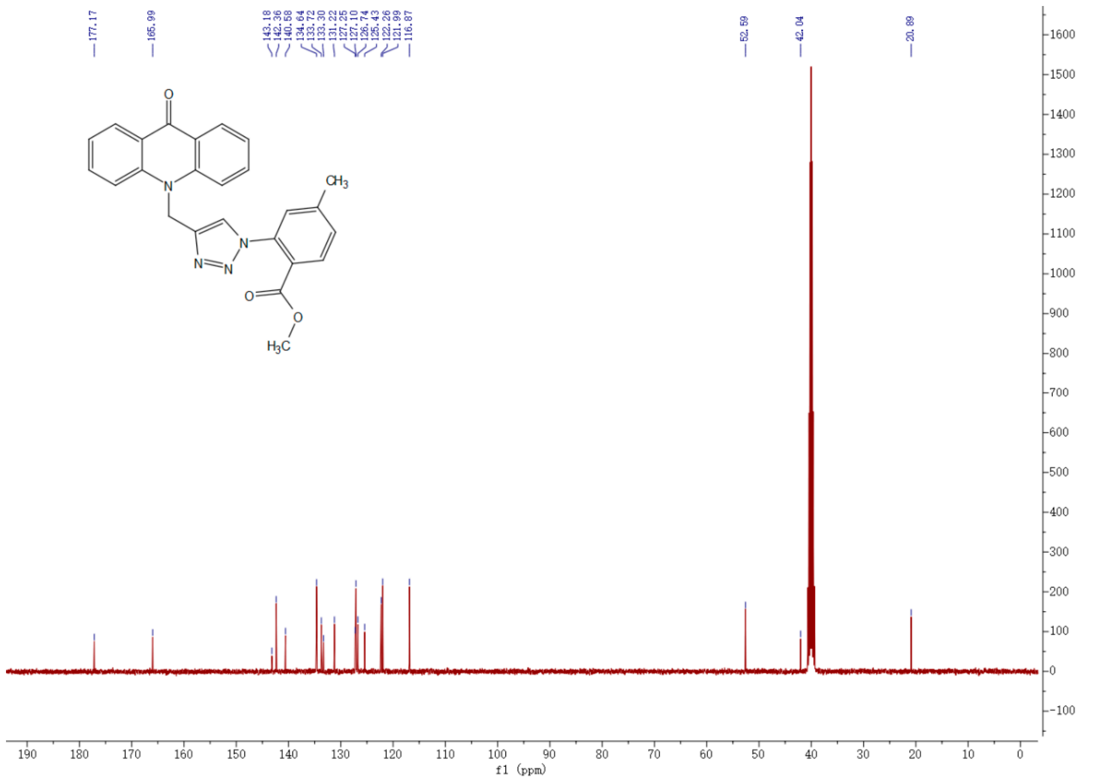

Figure S21. ^{13}C NMR of compound **L11**.

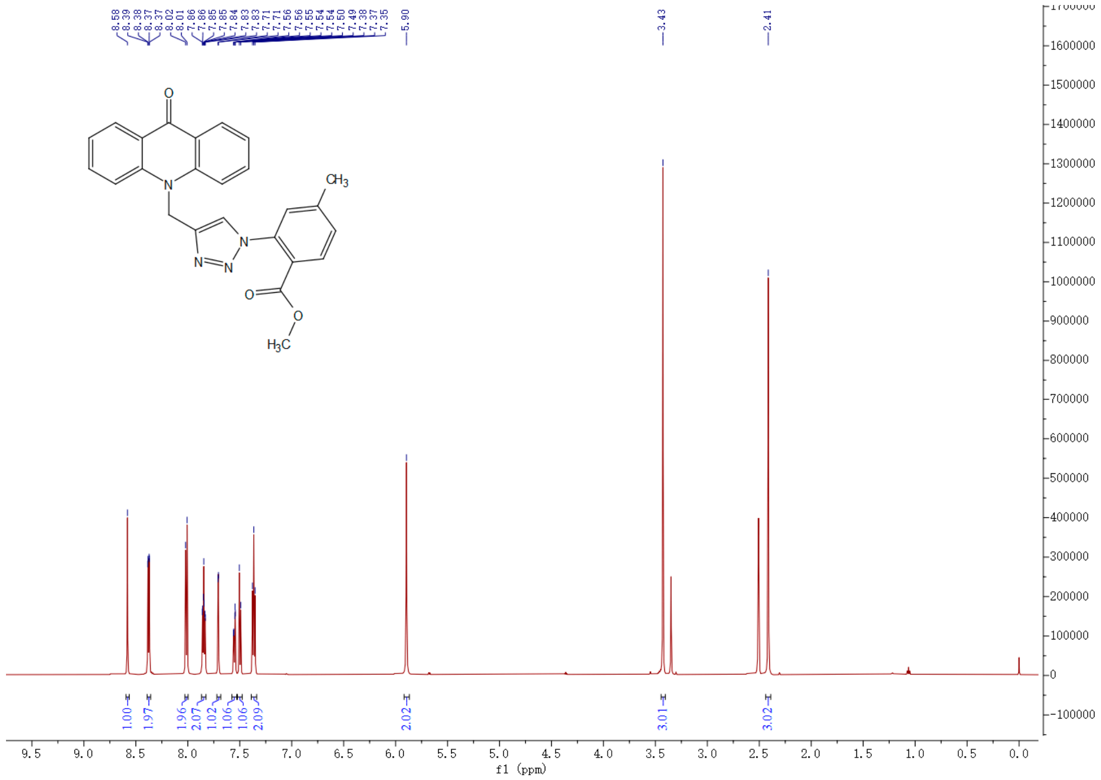

Figure S22. ^{1}H NMR of compound **L11**.

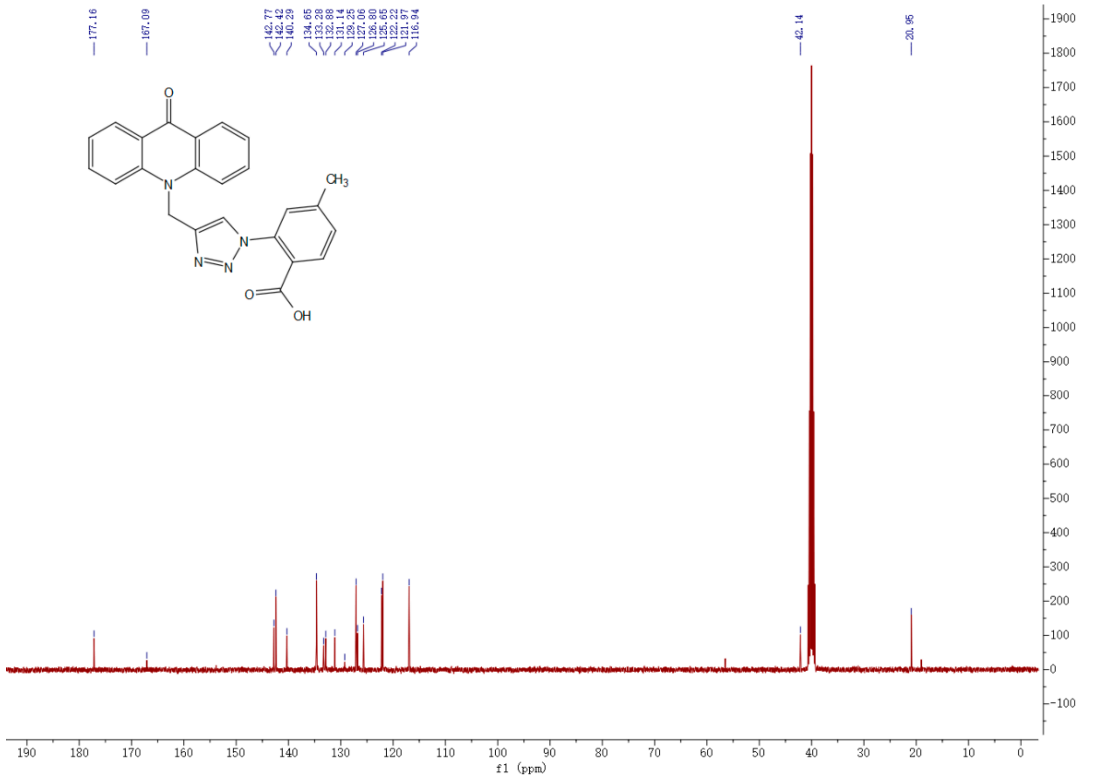

Figure S23. ^{13}C NMR of compound **L12**.

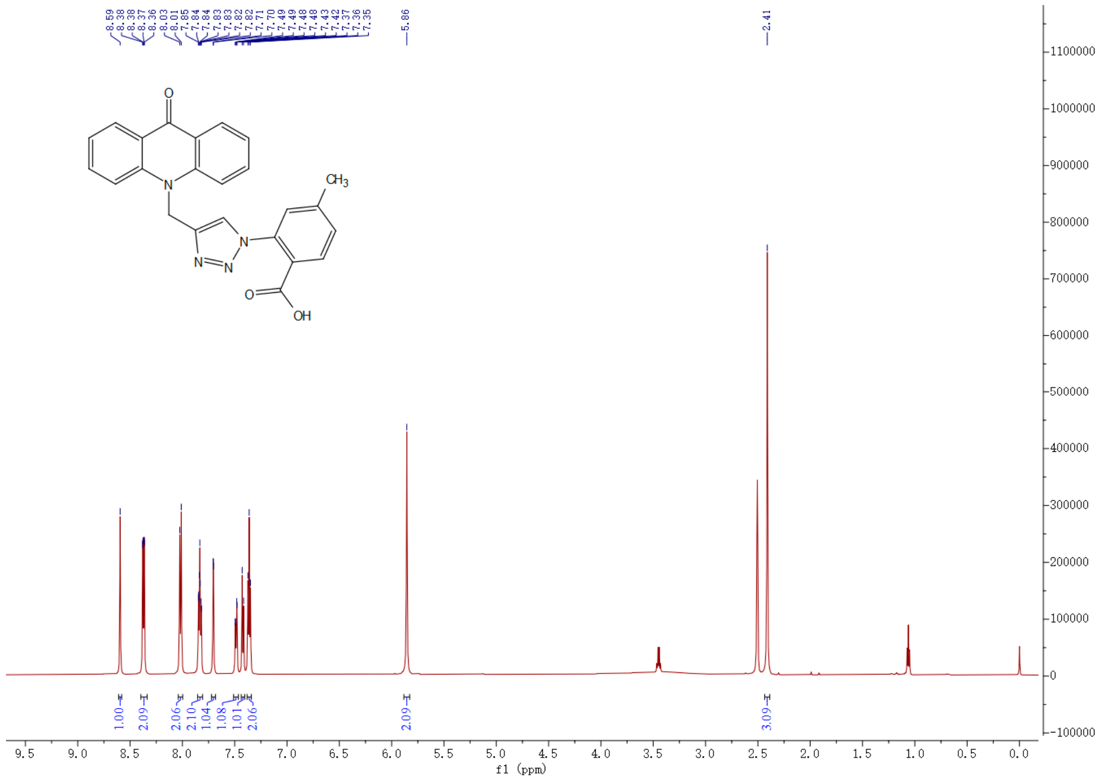

Figure S24. ^{1}H NMR of compound **L12**.

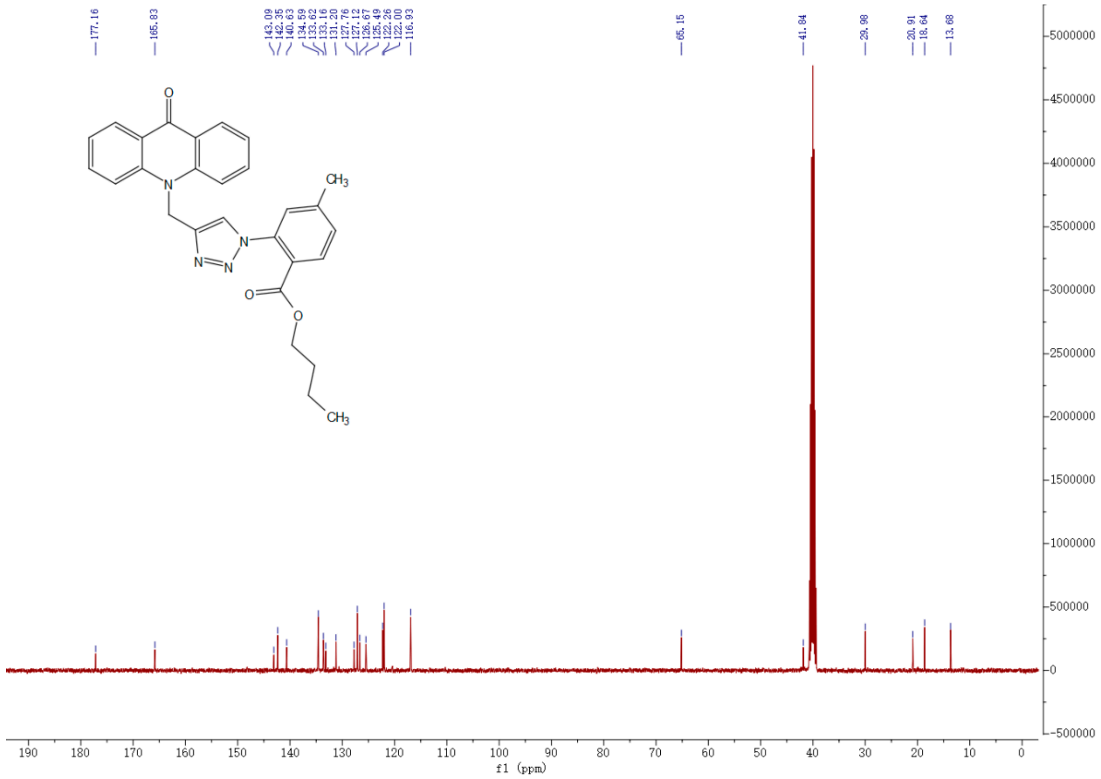

Figure S25. ^{13}C NMR of compound **L13**.

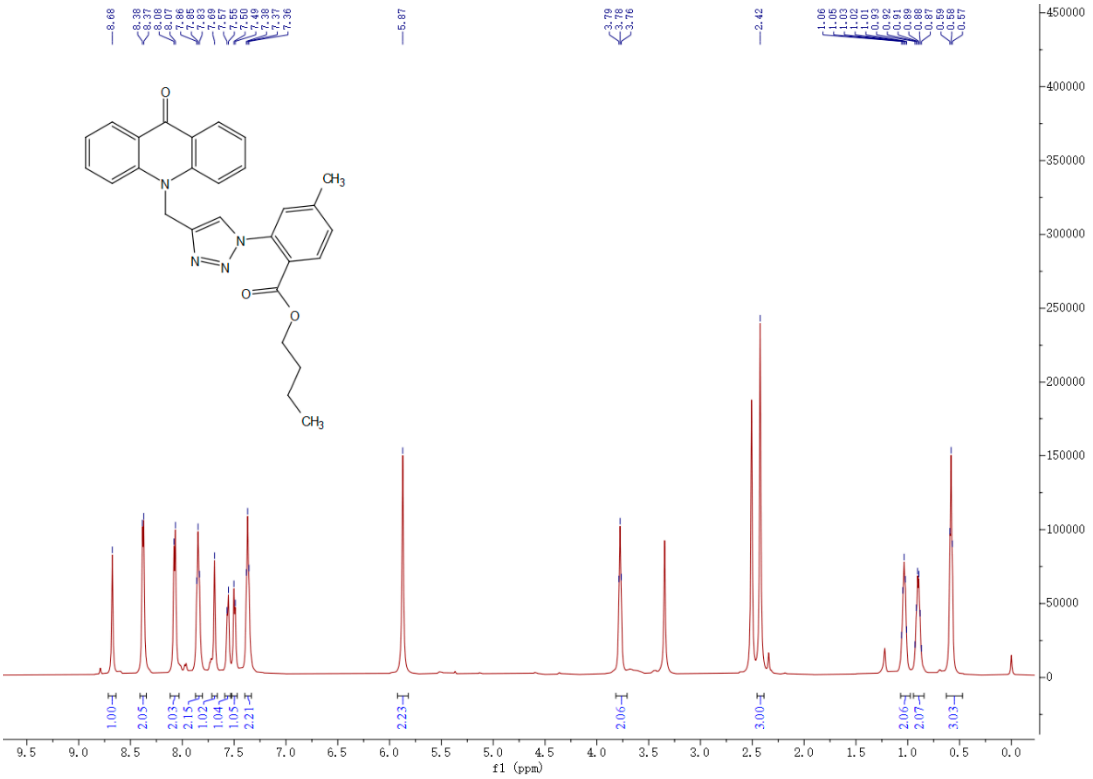

Figure S26. ^{1}H NMR of compound **L13**.

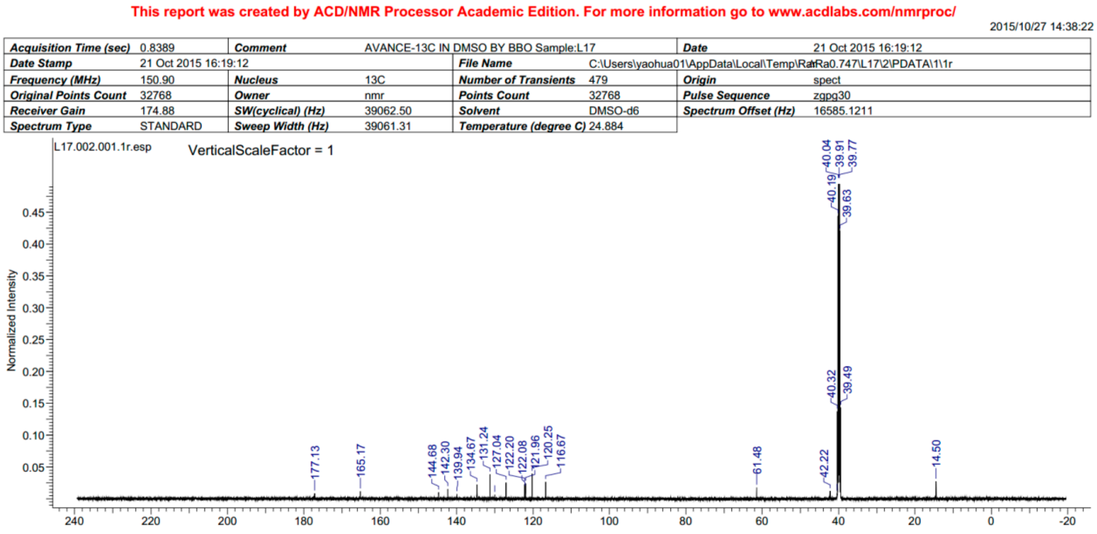

Figure S27. ^{13}C NMR of compound **L14**.

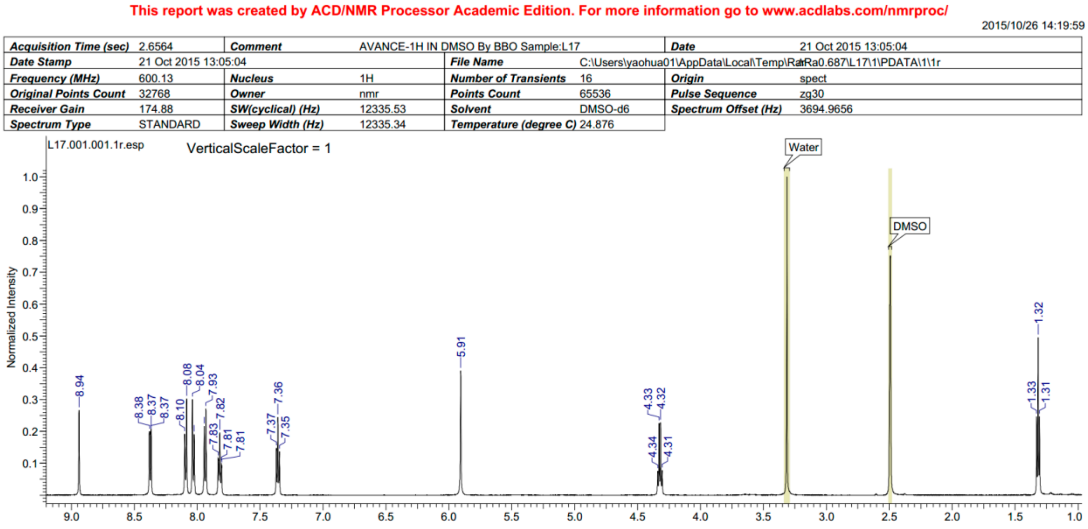

Figure S28. ^{1}H NMR of compound **L14**.

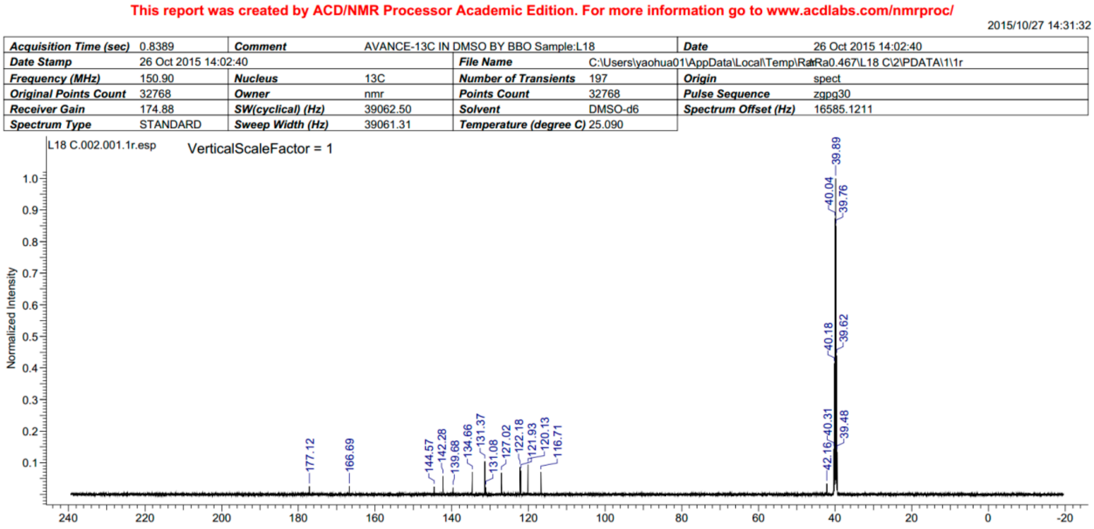

Figure S29. ^{13}C NMR of compound **L15**.

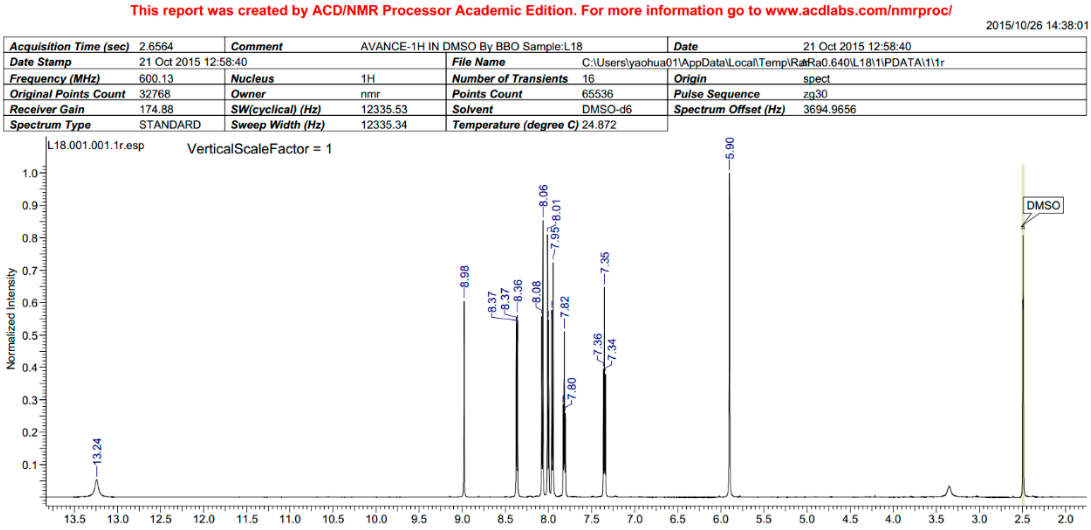

Figure S30. ^{1}H NMR of compound **L15**.

This report was created by ACD/NMR Processor Academic Edition. For more information go to www.acdlabs.com/nmrproc/

2015/10/27 14:28:57

Acquisition Time (sec)	0.8389	Comment	AVANCE-13C IN DMSO BY BBO Sample:L19			Date	26 Oct 2015 15:06:40
Date Stamp	26 Oct 2015 15:06:40			File Name	C:\Users\yaohua01\AppData\Local\Temp\RatRa0.698\L19 C\2\PDATA\1\1r		
Frequency (MHz)	150.90	Nucleus	13C	Number of Transients	1465	Origin	spect
Original Points Count	32768	Owner	nmr	Points Count	32768	Pulse Sequence	zgpg30
Receiver Gain	174.88	SW(cyclical) (Hz)	39062.50	Solvent	DMSO-d6	Spectrum Offset (Hz)	16585.1211
Spectrum Type	STANDARD	Sweep Width (Hz)	39061.31	Temperature (degree C)	25.101		

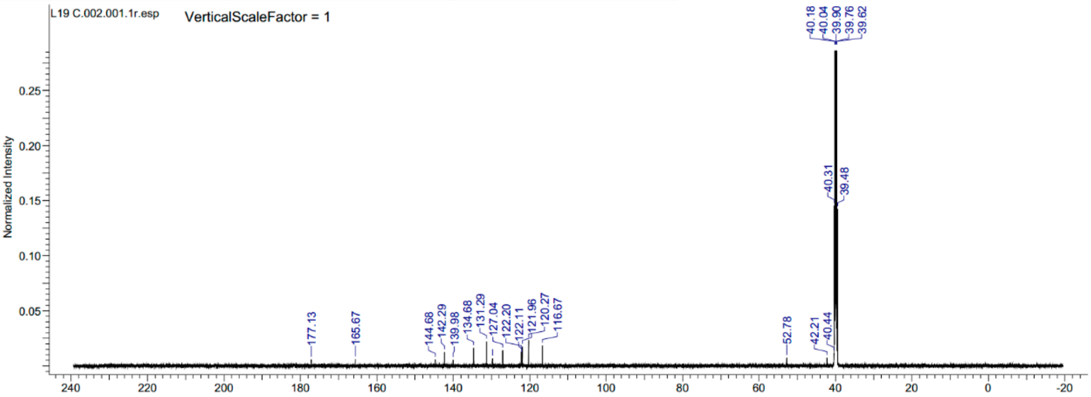

Figure S31. ^{13}C NMR of compound **L16**.

This report was created by ACD/NMR Processor Academic Edition. For more information go to www.acdlabs.com/nmrproc/

2015/10/26 14:57:12

Acquisition Time (sec)	2.6564	Comment	AVANCE-1H IN DMSO By BBO Sample:L19			Date	21 Oct 2015 13:09:20
Date Stamp	21 Oct 2015 13:09:20			File Name	C:\Users\yaohua01\AppData\Local\Temp\RatRa0.650\L19\1\PDATA\1\1r		
Frequency (MHz)	600.13	Nucleus	1H	Number of Transients	16	Origin	spect
Original Points Count	32768	Owner	nmr	Points Count	65536	Pulse Sequence	zg30
Receiver Gain	174.88	SW(cyclical) (Hz)	12335.53	Solvent	DMSO-d6	Spectrum Offset (Hz)	3694.9656
Spectrum Type	STANDARD	Sweep Width (Hz)	12335.34	Temperature (degree C)	24.878		

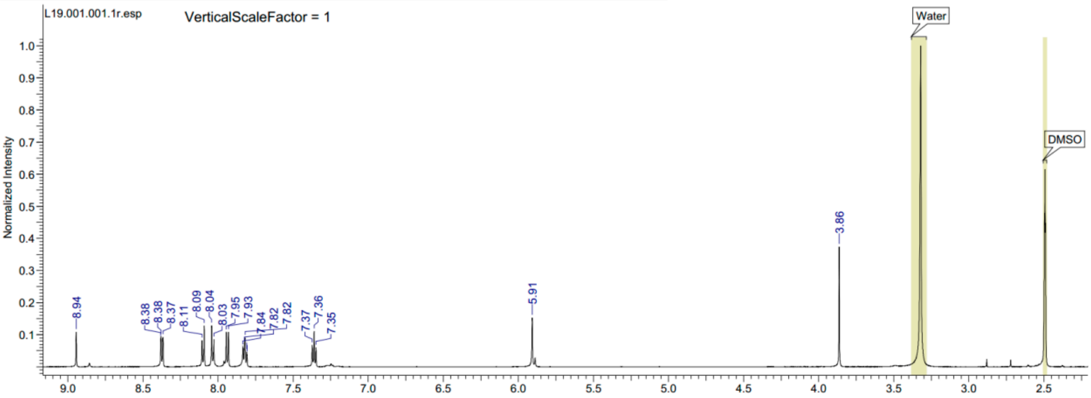

Figure S32. ^{1}H NMR of compound **L16**.

This report was created by ACD/NMR Processor Academic Edition. For more information go to www.acdlabs.com/nmrproc/

2015/10/27 14:22:59

Acquisition Time (sec)	0.8389	Comment	AVANCE-13C IN DMSO BY BBO Sample:L20			Date	21 Oct 2015 16:02:08
Date Stamp	21 Oct 2015 16:02:08			File Name	C:\Users\yaohua01\AppData\Local\Temp\RatRa0.078\L20\2\PDATA\1\1r		
Frequency (MHz)	150.90	Nucleus	13C	Number of Transients	389	Origin	spect
Original Points Count	32768	Owner	nmr	Points Count	32768	Pulse Sequence	zgpg30
Receiver Gain	174.88	SW(cyclical) (Hz)	39062.50	Solvent	DMSO-d6	Spectrum Offset (Hz)	16585.1211
Spectrum Type	STANDARD	Sweep Width (Hz)	39061.31	Temperature (degree C)	24.884		

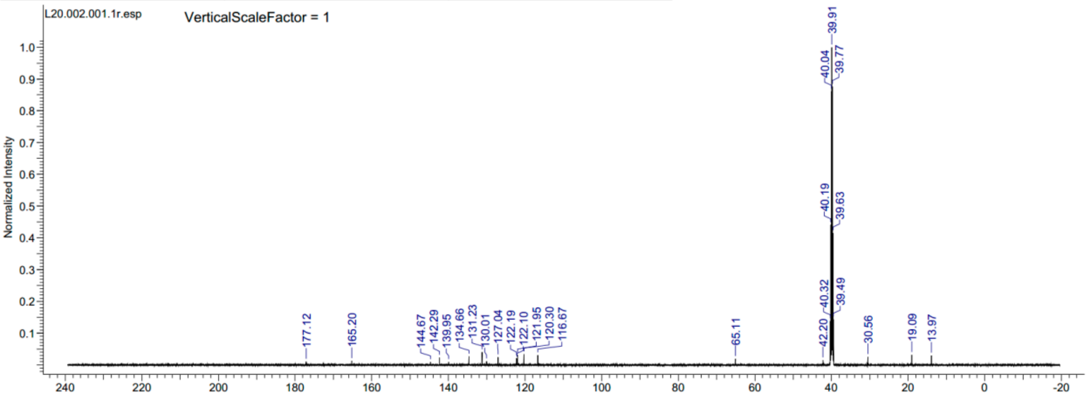

Figure S33. ^{13}C NMR of compound **L17**.

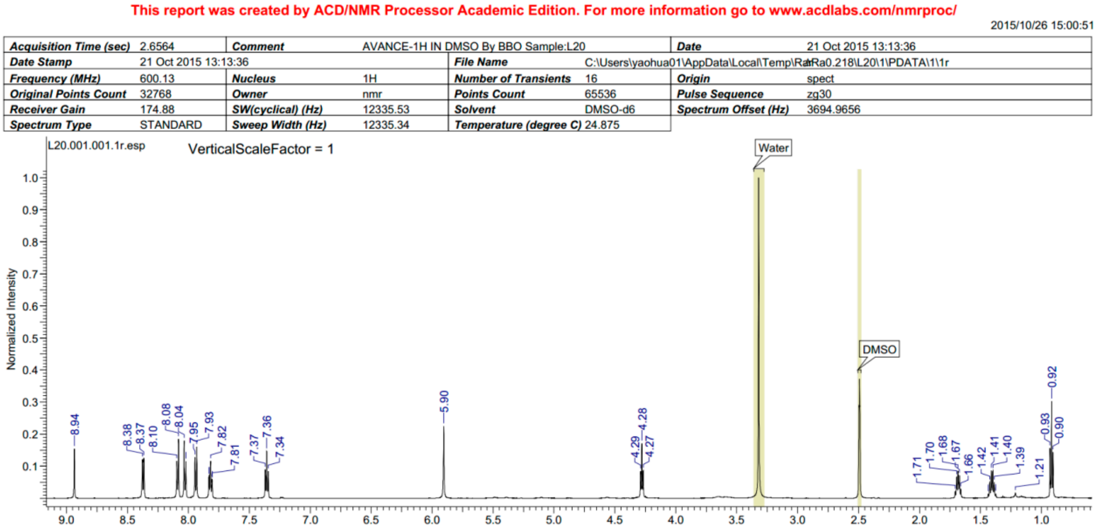

Figure S34. ^{1}H NMR of compound **L17**.

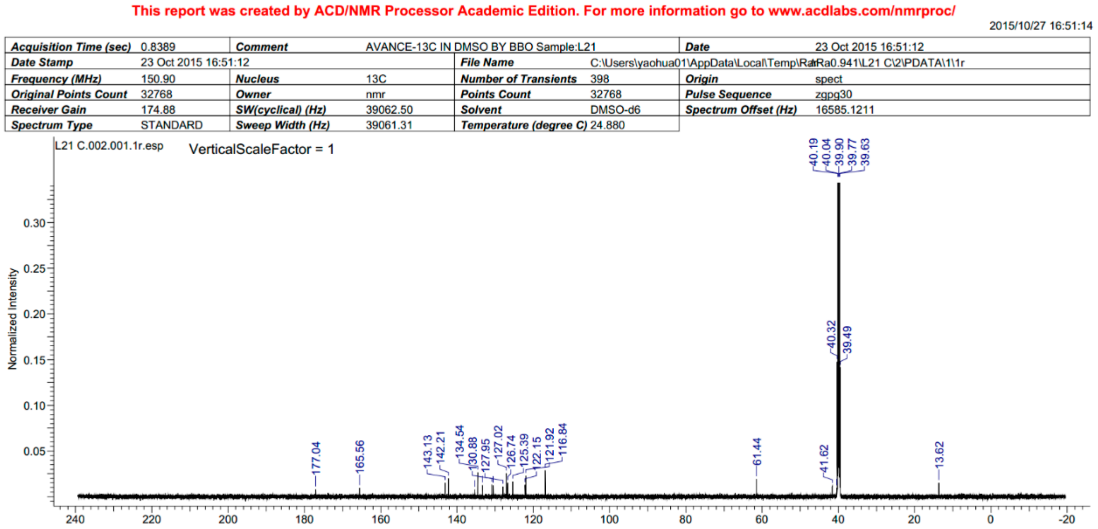

Figure S35. ^{13}C NMR of compound **L18**.

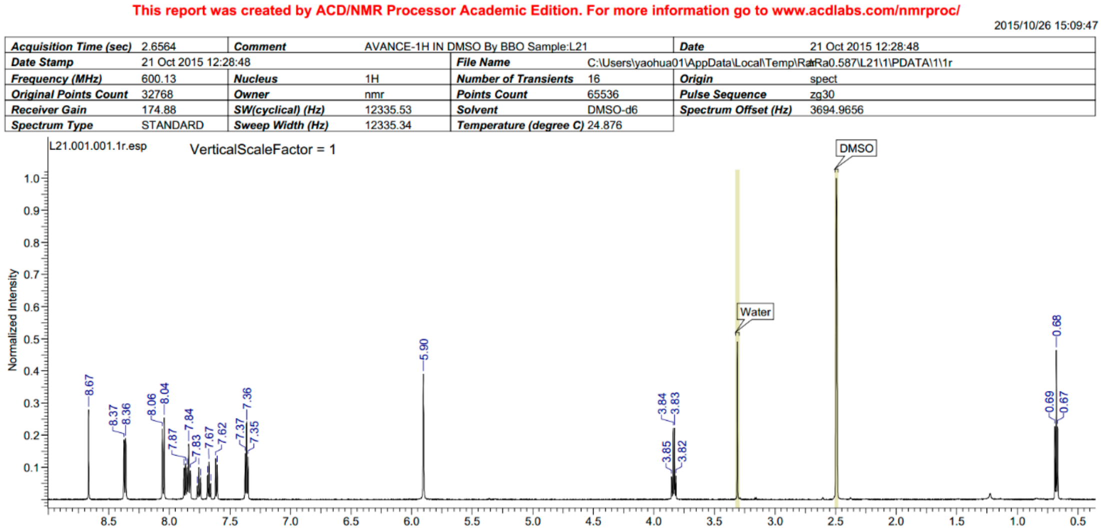

Figure S36. ^{1}H NMR of compound L18.

This report was created by ACD/NMR Processor Academic Edition. For more information go to www.acdlabs.com/nmrproc/

2015/10/27 9:47:34

Acquisition Time (sec)	0.8389	*Comment*	AVANCE-13C IN DMSO BY BBO Sample:L22			*Date*	23 Oct 2015 16:06:24
Date Stamp	23 Oct 2015 16:06:24			*File Name*	C:\Users\yaohua01\AppData\Local\Temp\RaRa0.408\L22 C\2\PDATA\1\1r		
Frequency (MHz)	150.90	*Nucleus*	13C	*Number of Transients*	295	*Origin*	spect
Original Points Count	32768	*Owner*	nmr	*Points Count*	32768	*Pulse Sequence*	zgpg30
Receiver Gain	174.88	*SW(cyclical) (Hz)*	39062.50	*Solvent*	DMSO-d6	*Spectrum Offset (Hz)*	16585.1211
Spectrum Type	STANDARD	*Sweep Width (Hz)*	39061.31	*Temperature (degree C)*	24.882		

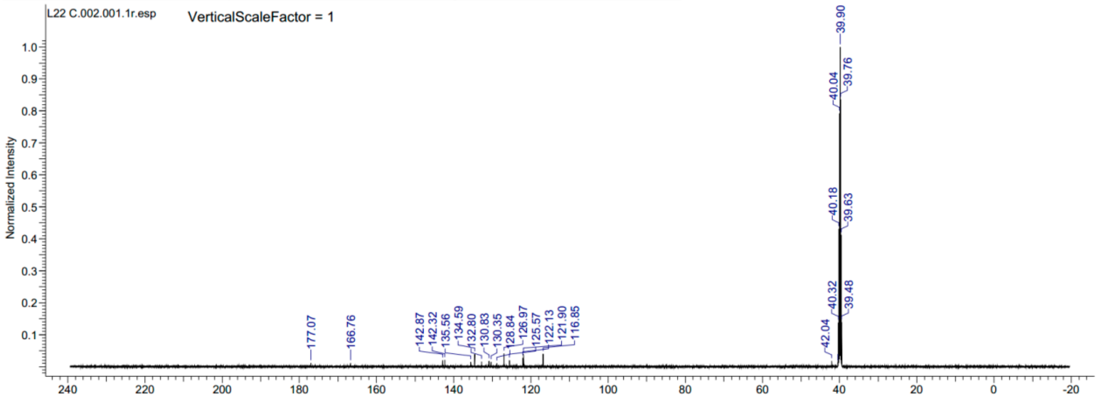

Figure S37. ^{13}C NMR of compound **L19**.

This report was created by ACD/NMR Processor Academic Edition. For more information go to www.acdlabs.com/nmrproc/

2015/10/26 16:17:03

Acquisition Time (sec)	2.6564	*Comment*	AVANCE-1H IN DMSO By BBO Sample:L22			*Date*	21 Oct 2015 13:26:24
Date Stamp	21 Oct 2015 13:26:24			*File Name*	C:\Users\yaohua01\AppData\Local\Temp\RaRa0.120\L22\1\PDATA\1\1r		
Frequency (MHz)	600.13	*Nucleus*	1H	*Number of Transients*	16	*Origin*	spect
Original Points Count	32768	*Owner*	nmr	*Points Count*	65536	*Pulse Sequence*	zg30
Receiver Gain	174.88	*SW(cyclical) (Hz)*	12335.53	*Solvent*	DMSO-d6	*Spectrum Offset (Hz)*	3694.9656
Spectrum Type	STANDARD	*Sweep Width (Hz)*	12335.34	*Temperature (degree C)*	24.878		

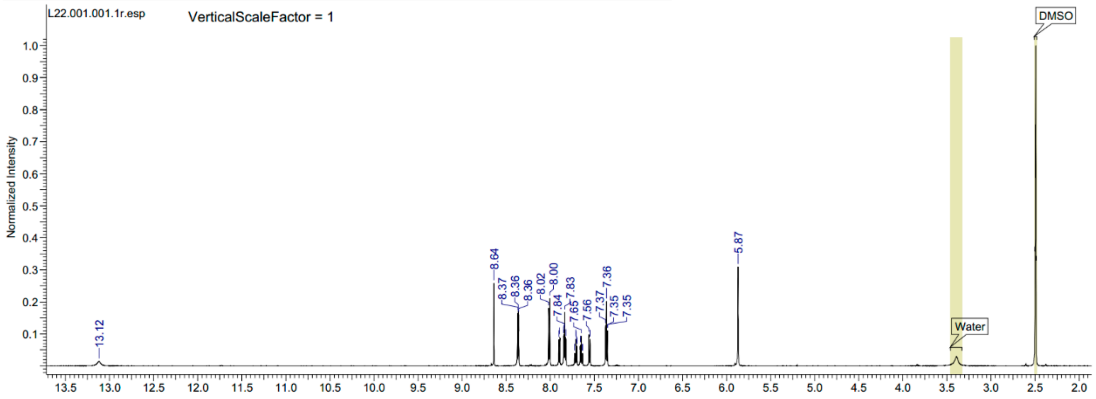

Figure S38. ^{1}H NMR of compound **L19**.

This report was created by ACD/NMR Processor Academic Edition. For more information go to www.acdlabs.com/nmrproc/

2015/10/27 9:36:47

Acquisition Time (sec)	0.8389	*Comment*	AVANCE-13C IN DMSO BY BBO Sample:L23			*Date*	26 Oct 2015 09:53:04
Date Stamp	26 Oct 2015 09:53:04			*File Name*	C:\Users\yaohua01\AppData\Local\Temp\RaRa0.980\L23 C\2\PDATA\1\1r		
Frequency (MHz)	150.90	*Nucleus*	13C	*Number of Transients*	473	*Origin*	spect
Original Points Count	32768	*Owner*	nmr	*Points Count*	32768	*Pulse Sequence*	zgpg30
Receiver Gain	174.88	*SW(cyclical) (Hz)*	39062.50	*Solvent*	DMSO-d6	*Spectrum Offset (Hz)*	16585.1211
Spectrum Type	STANDARD	*Sweep Width (Hz)*	39061.31	*Temperature (degree C)*	24.907		

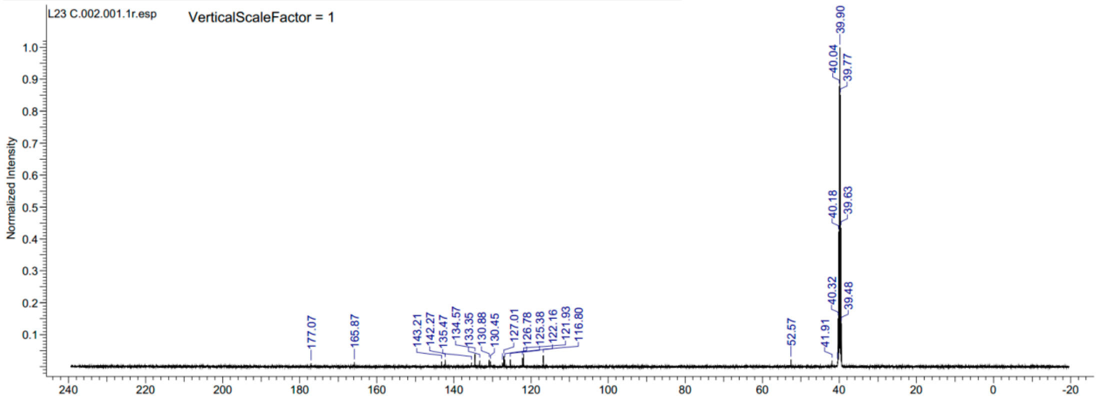

Figure S39. ^{13}C NMR of compound **L20**.

This report was created by ACD/NMR Processor Academic Edition. For more information go to www.acdlabs.com/nmrproc/

2015/10/26 16:52:09

**Acquisition Time (sec)**	2.6564	**Comment**	AVANCE-1H IN DMSO By BBO Sample:L23			**Date**	21 Oct 2015 12:54:24
**Date Stamp**	21 Oct 2015 12:54:24			**File Name**	C:\Users\yaohua01\AppData\Local\Temp\RaRa0.617\L23\1\PDATA\1\1r		
**Frequency (MHz)**	600.13	**Nucleus**	1H	**Number of Transients**	16	**Origin**	spect
**Original Points Count**	32768	**Owner**	nmr	**Points Count**	65536	**Pulse Sequence**	zg30
**Receiver Gain**	174.88	**SW(cyclical) (Hz)**	12335.53	**Solvent**	DMSO-d6	**Spectrum Offset (Hz)**	3694.9656
**Spectrum Type**	STANDARD	**Sweep Width (Hz)**	12335.34	**Temperature (degree C)**	24.873		

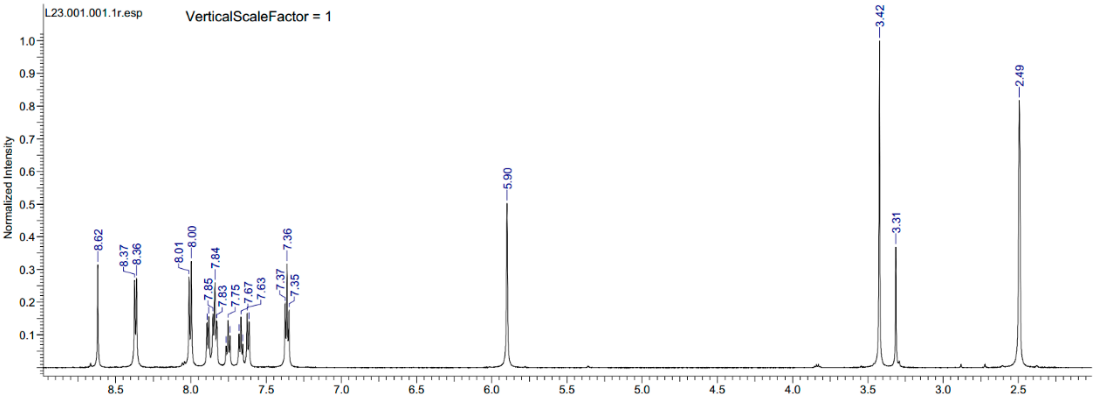

Figure S40. ^{1}H NMR of compound **L20**.

This report was created by ACD/NMR Processor Academic Edition. For more information go to www.acdlabs.com/nmrproc/

2015/10/27 10:46:47

**Acquisition Time (sec)**	0.8389	**Comment**	AVANCE-13C IN DMSO BY BBO Sample:L24			**Date**	21 Oct 2015 15:38:40
**Date Stamp**	21 Oct 2015 15:38:40			**File Name**	C:\Users\yaohua01\AppData\Local\Temp\RaRa0.414\L24\2\PDATA\1\1r		
**Frequency (MHz)**	150.90	**Nucleus**	13C	**Number of Transients**	103	**Origin**	spect
**Original Points Count**	32768	**Owner**	nmr	**Points Count**	32768	**Pulse Sequence**	zgpg30
**Receiver Gain**	174.88	**SW(cyclical) (Hz)**	39062.50	**Solvent**	DMSO-d6	**Spectrum Offset (Hz)**	16585.1211
**Spectrum Type**	STANDARD	**Sweep Width (Hz)**	39061.31	**Temperature (degree C)**	24.899		

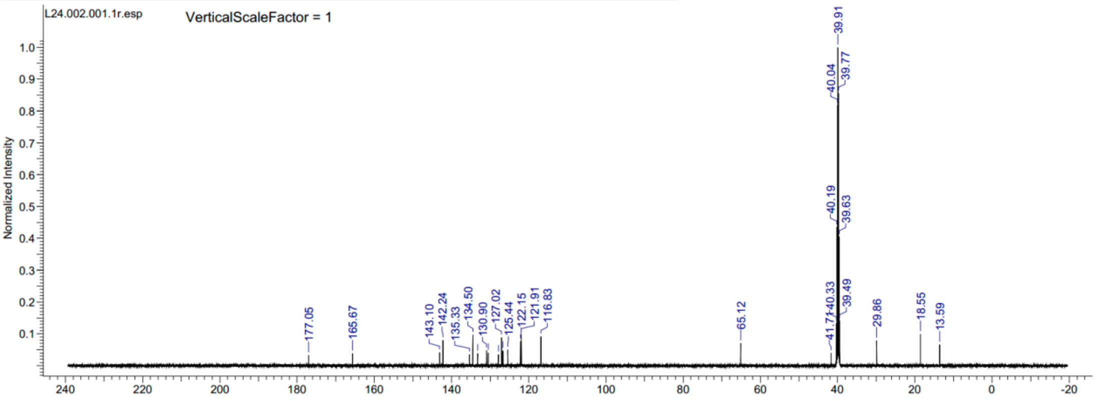

Figure S41. ^{13}C NMR of compound **L21**.

This report was created by ACD/NMR Processor Academic Edition. For more information go to www.acdlabs.com/nmrproc/

2015/10/26 16:37:36

**Acquisition Time (sec)**	2.6564	**Comment**	AVANCE-1H IN DMSO By BBO Sample:L24			**Date**	21 Oct 2015 13:17:52
**Date Stamp**	21 Oct 2015 13:17:52			**File Name**	C:\Users\yaohua01\AppData\Local\Temp\RaRa0.498\L24\1\PDATA\1\1r		
**Frequency (MHz)**	600.13	**Nucleus**	1H	**Number of Transients**	16	**Origin**	spect
**Original Points Count**	32768	**Owner**	nmr	**Points Count**	65536	**Pulse Sequence**	zg30
**Receiver Gain**	174.88	**SW(cyclical) (Hz)**	12335.53	**Solvent**	DMSO-d6	**Spectrum Offset (Hz)**	3694.9656
**Spectrum Type**	STANDARD	**Sweep Width (Hz)**	12335.34	**Temperature (degree C)**	24.882		

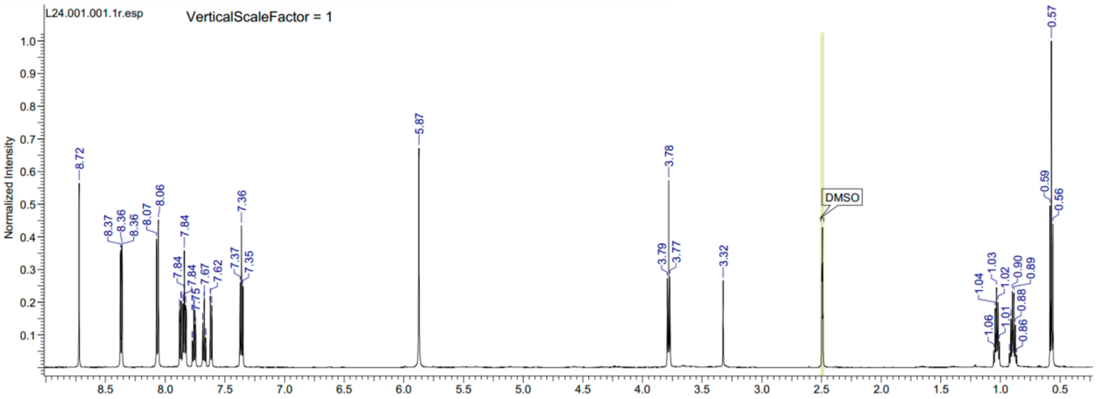

Figure S42. ^{1}H NMR of compound **L21**.

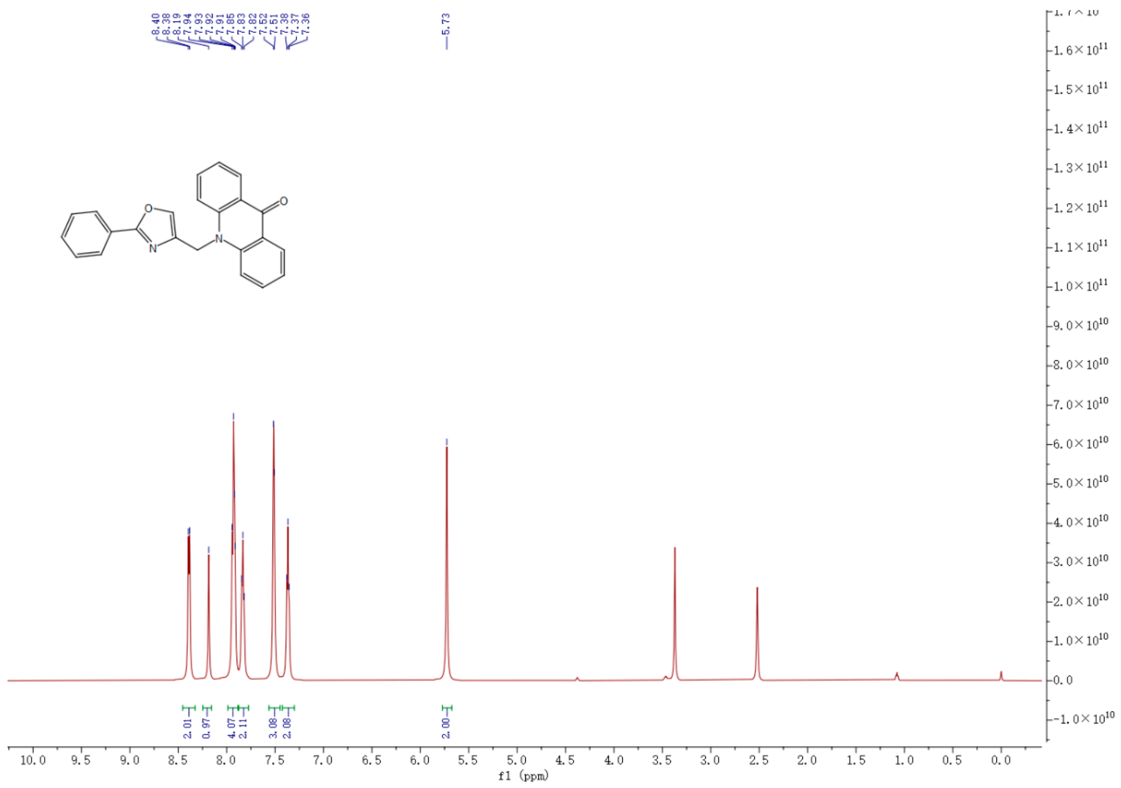

Figure S43. ^{1}H-NMR spectrum of compound **N1**.

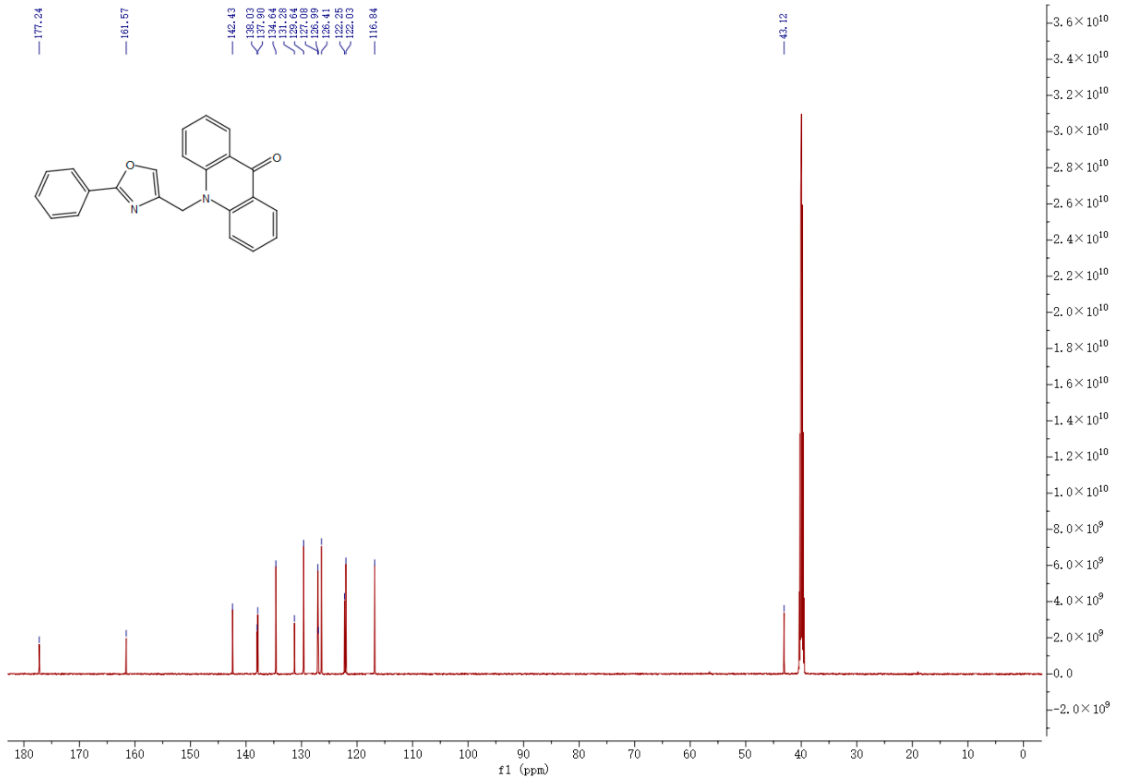

Figure S44. ^{13}C-NMR spectrum of compound **N1**.

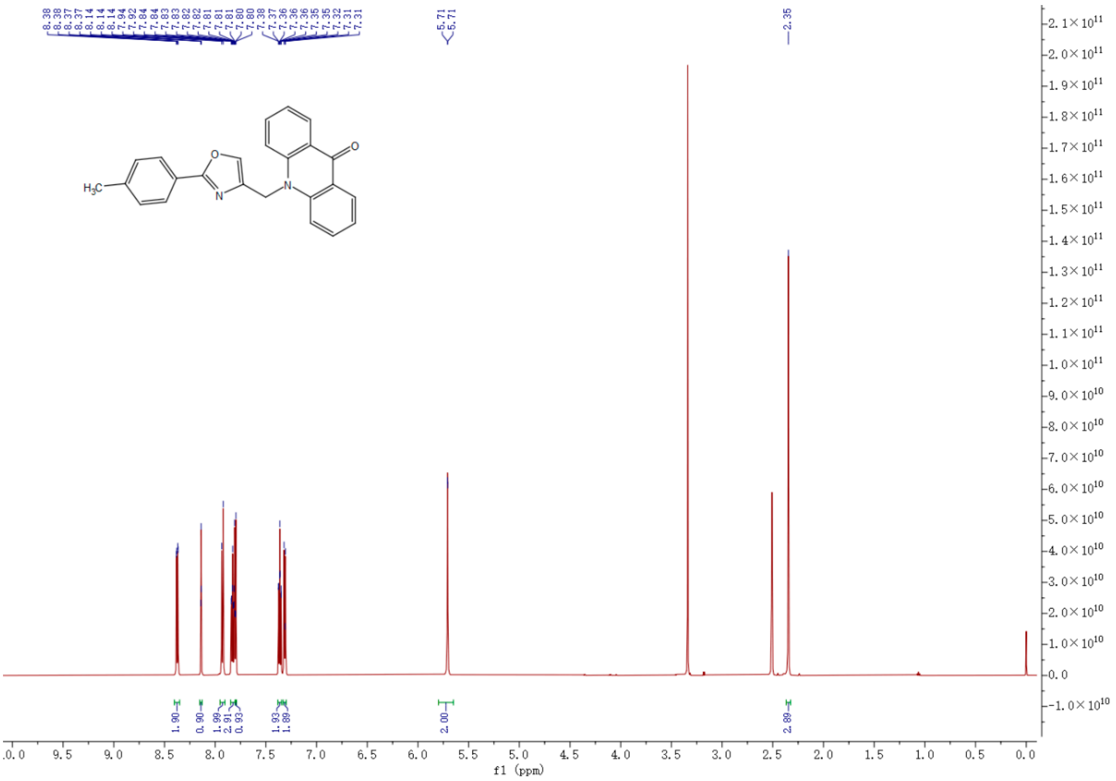

Figure S45. ^{1}H-NMR spectrum of compound **N2**.

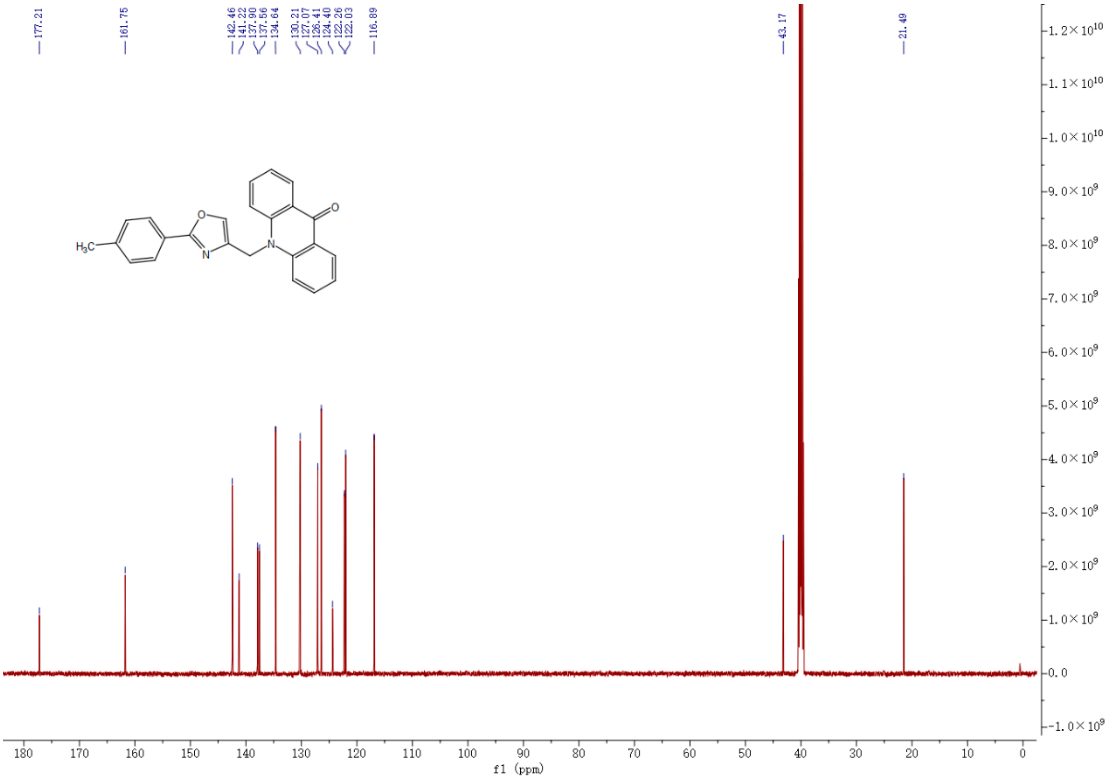

Figure S46. ^{13}C-NMR spectrum of compound **N2**.

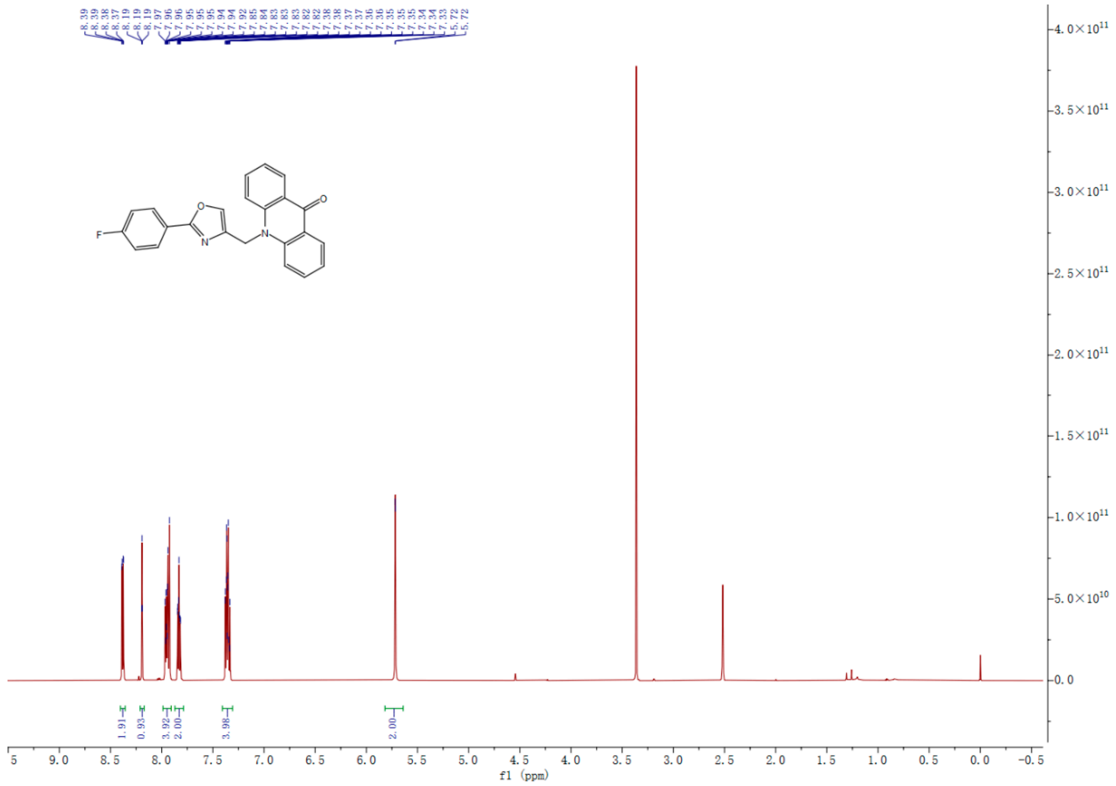

Figure S47. ^{1}H-NMR spectrum of compound **N3**.

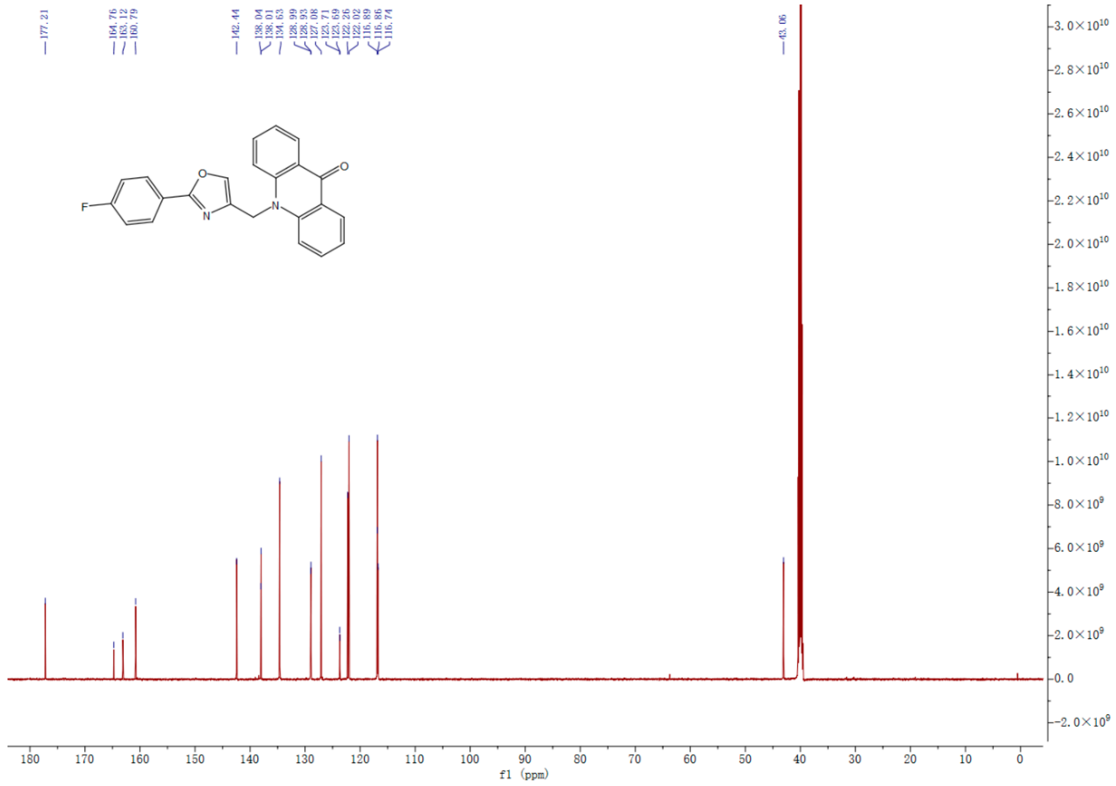

Figure S48. ^{13}C-NMR spectrum of compound **N3**.

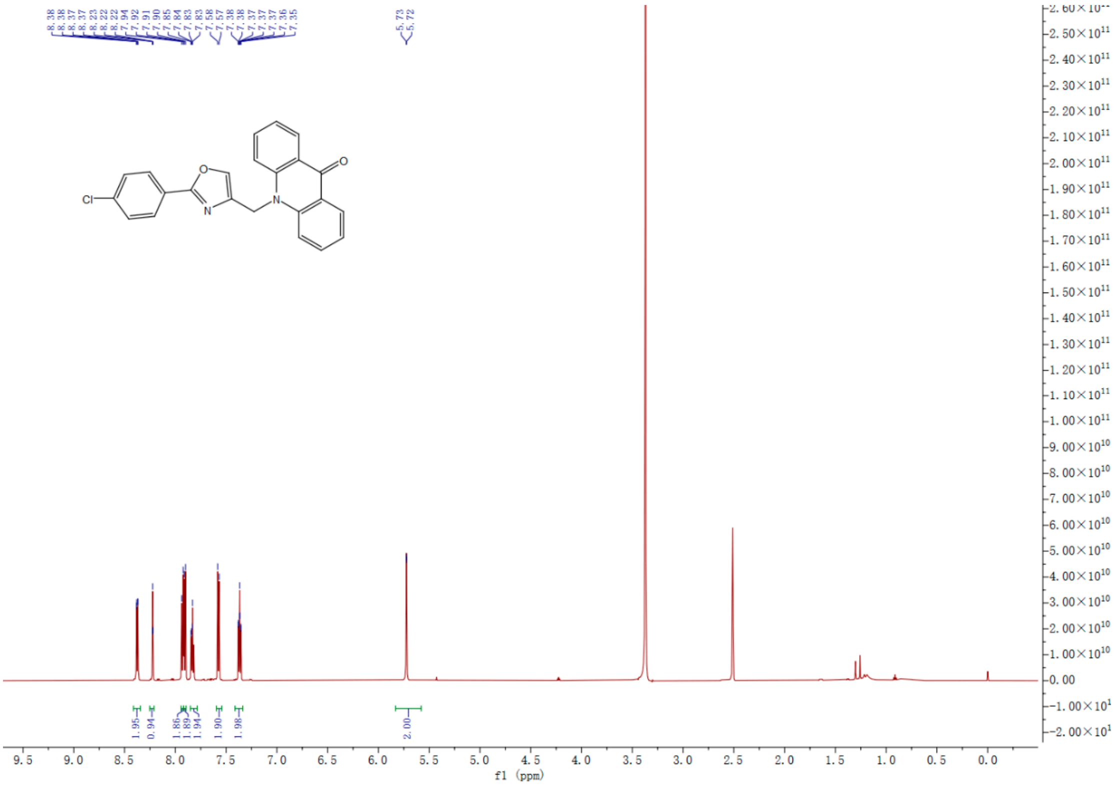

Figure S49. ^{1}H-NMR spectrum of compound **N4**.

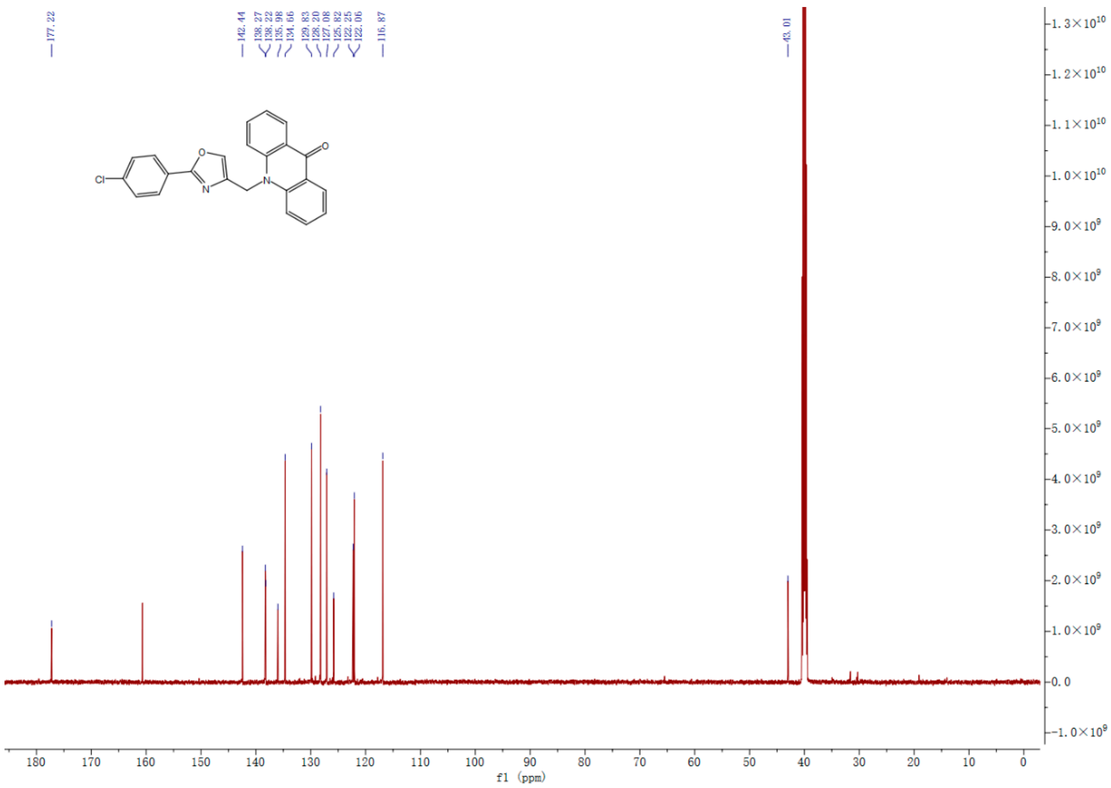

Figure S50. ^{13}C-NMR spectrum of compound **N4**.

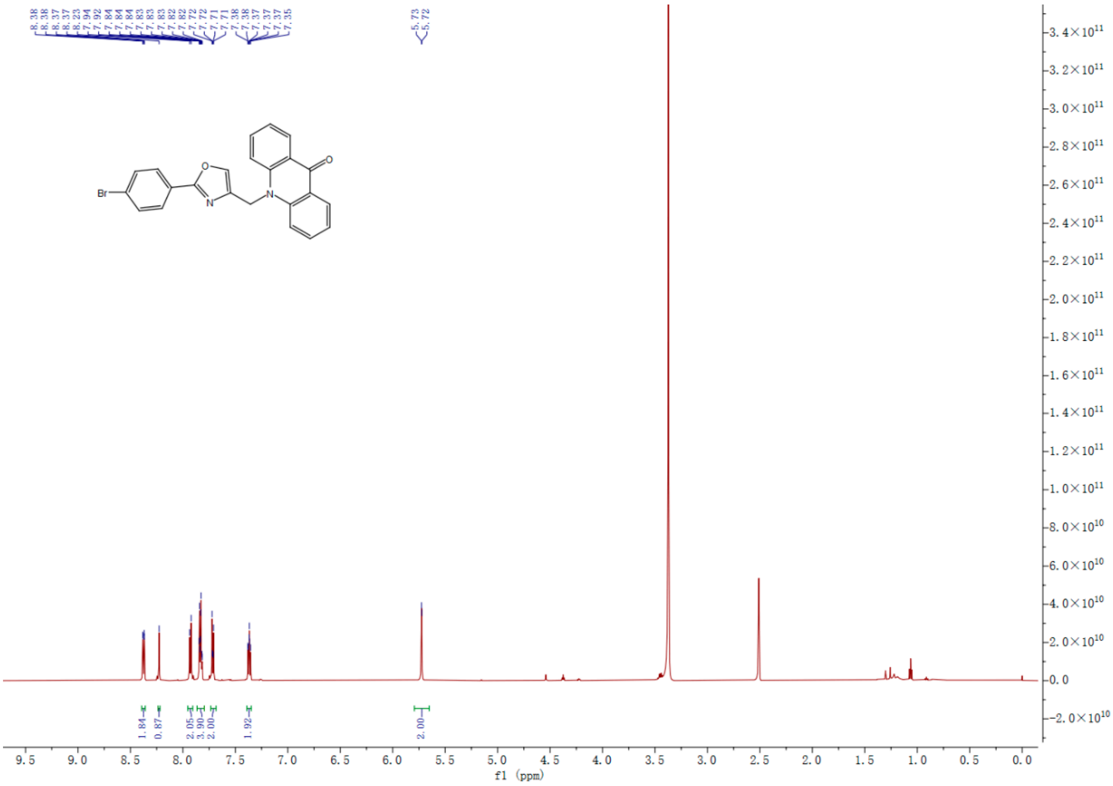

Figure S51. ^{1}H-NMR spectrum of compound **N5**.

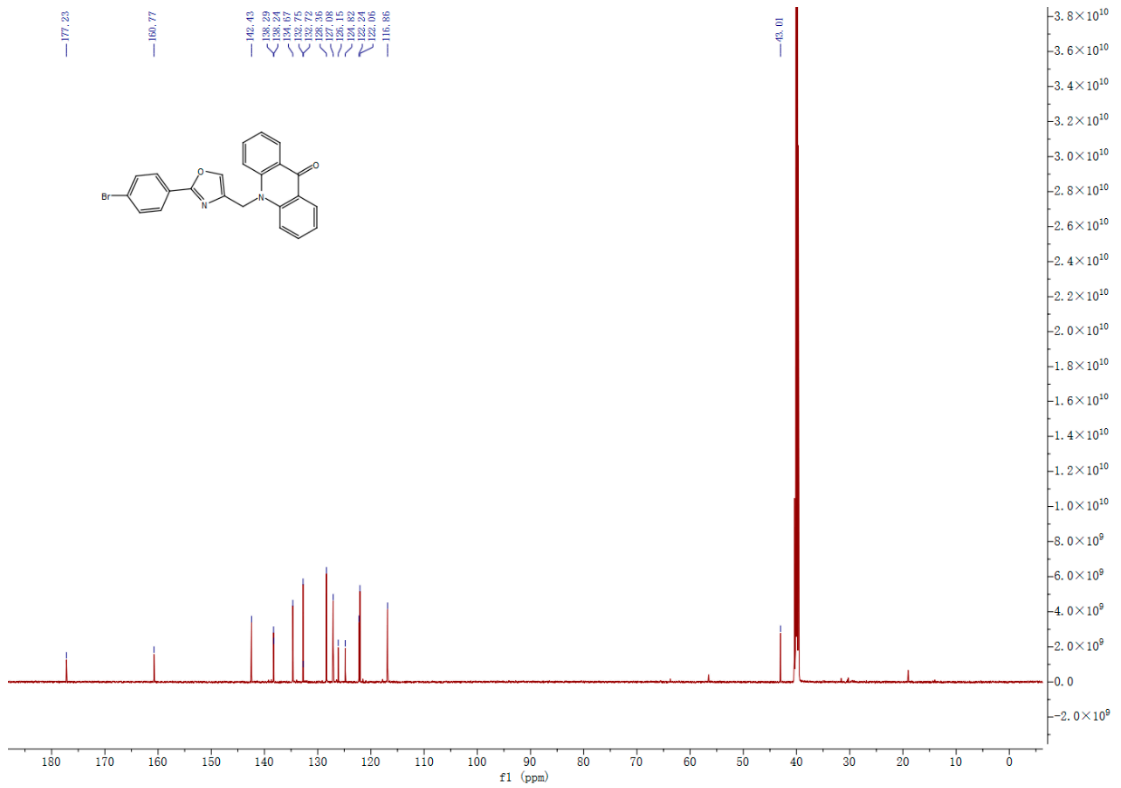

Figure S52. ^{13}C-NMR spectrum of compound **N5**.

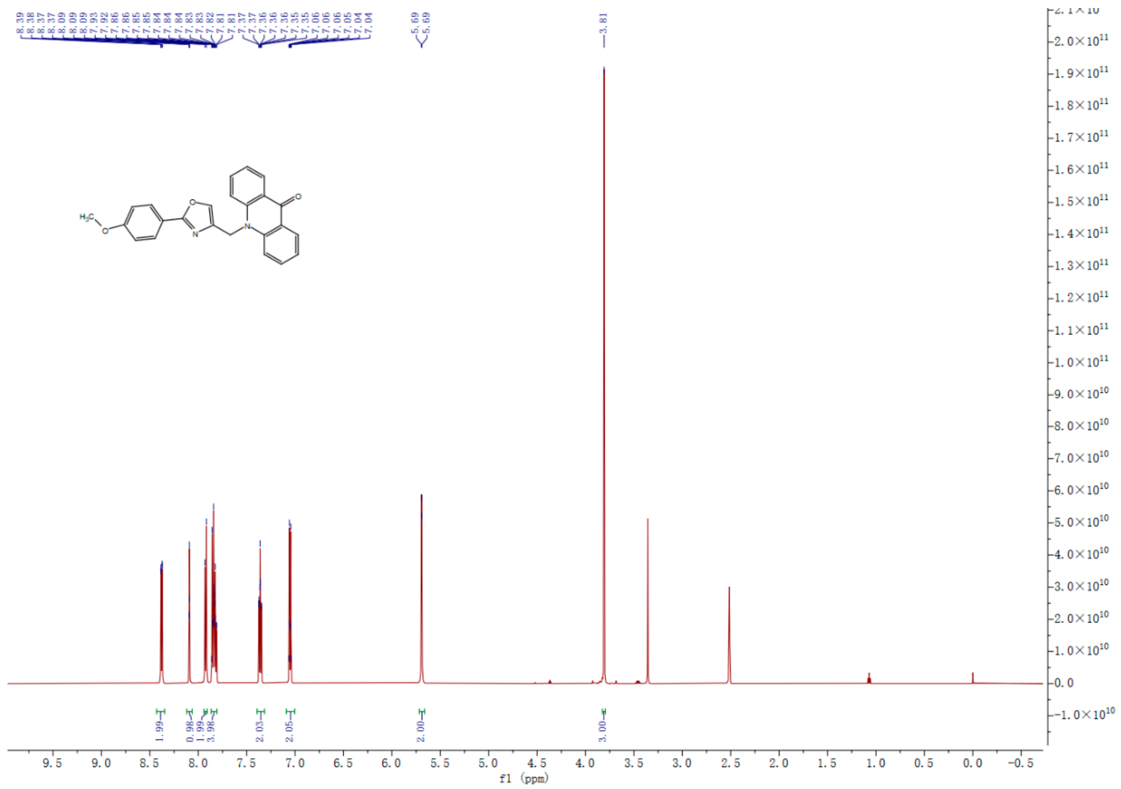

Figure S53. ^{1}H-NMR spectrum of compound **N6**.

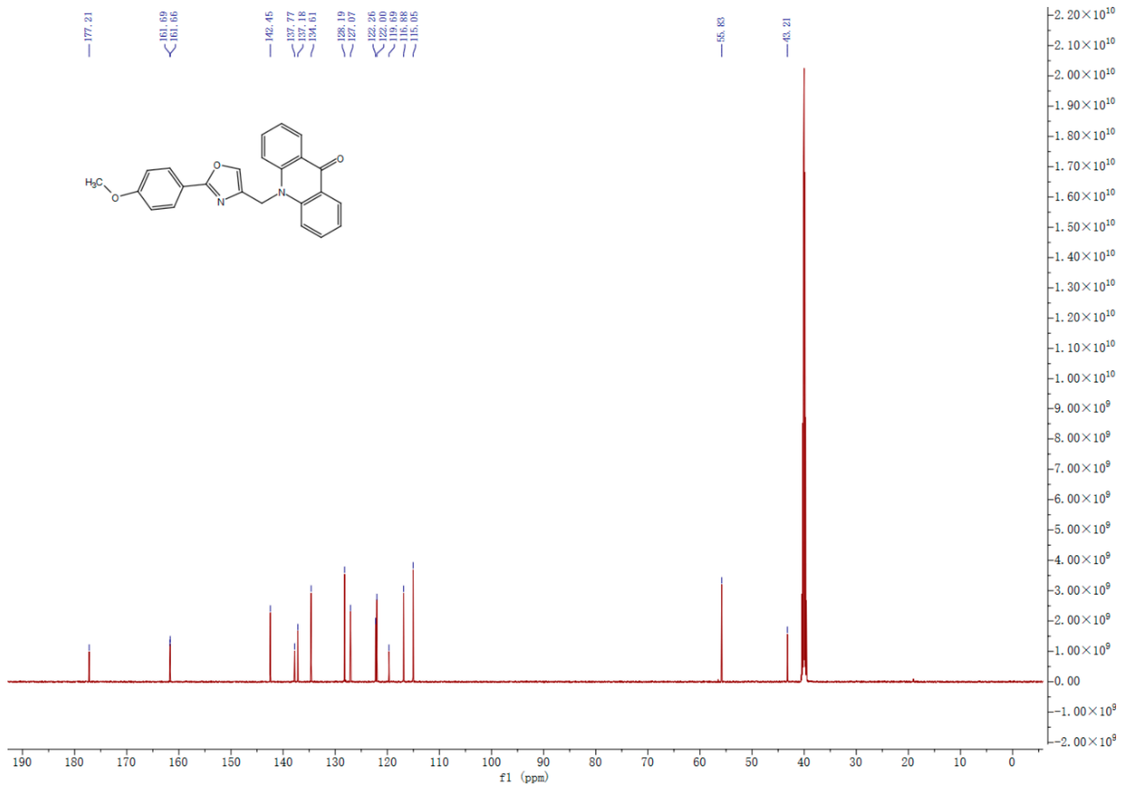

Figure S54. ^{13}C-NMR spectrum of compound **N6**.

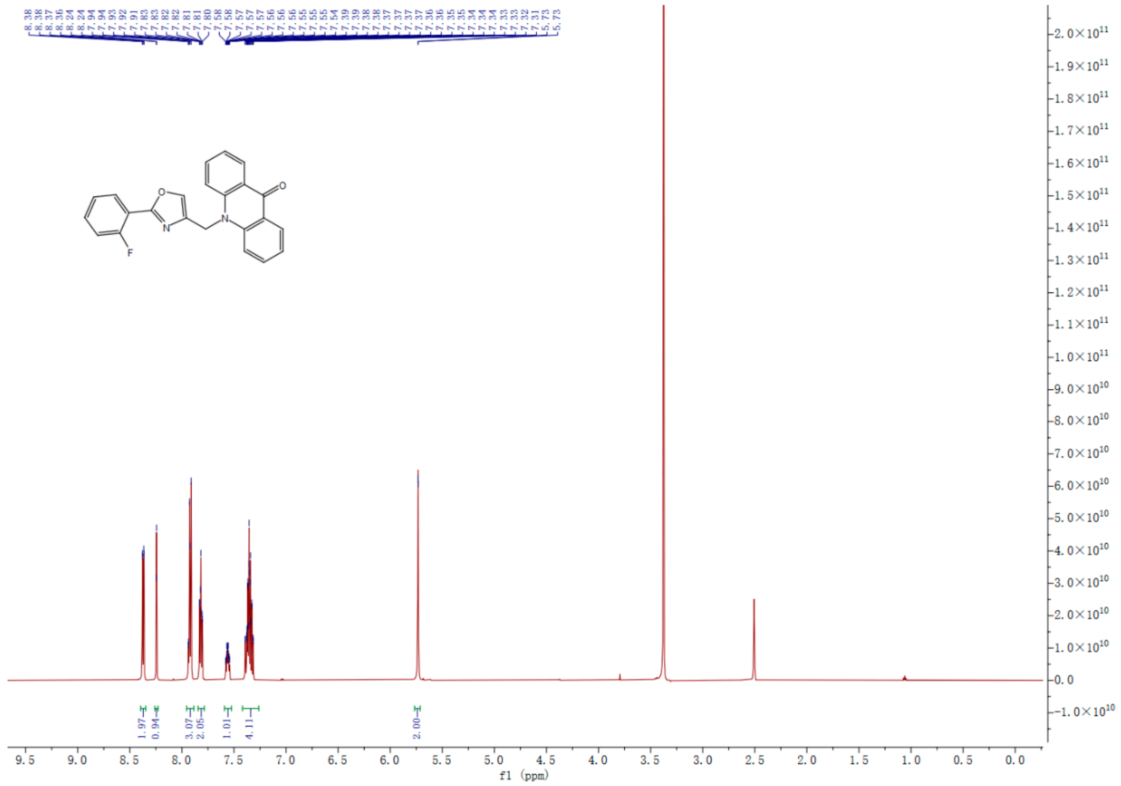

Figure S55. ^{1}H-NMR spectrum of compound **N7**.

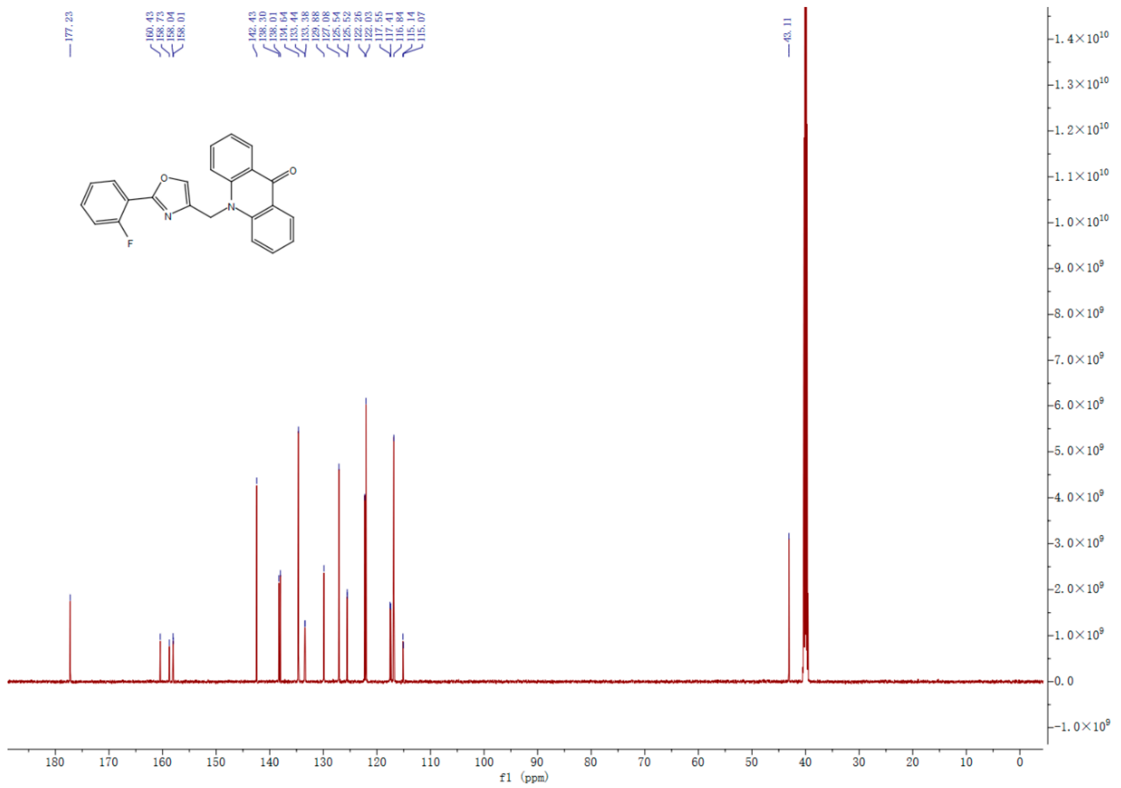

Figure S56. ^{13}C-NMR spectrum of compound **N7**.

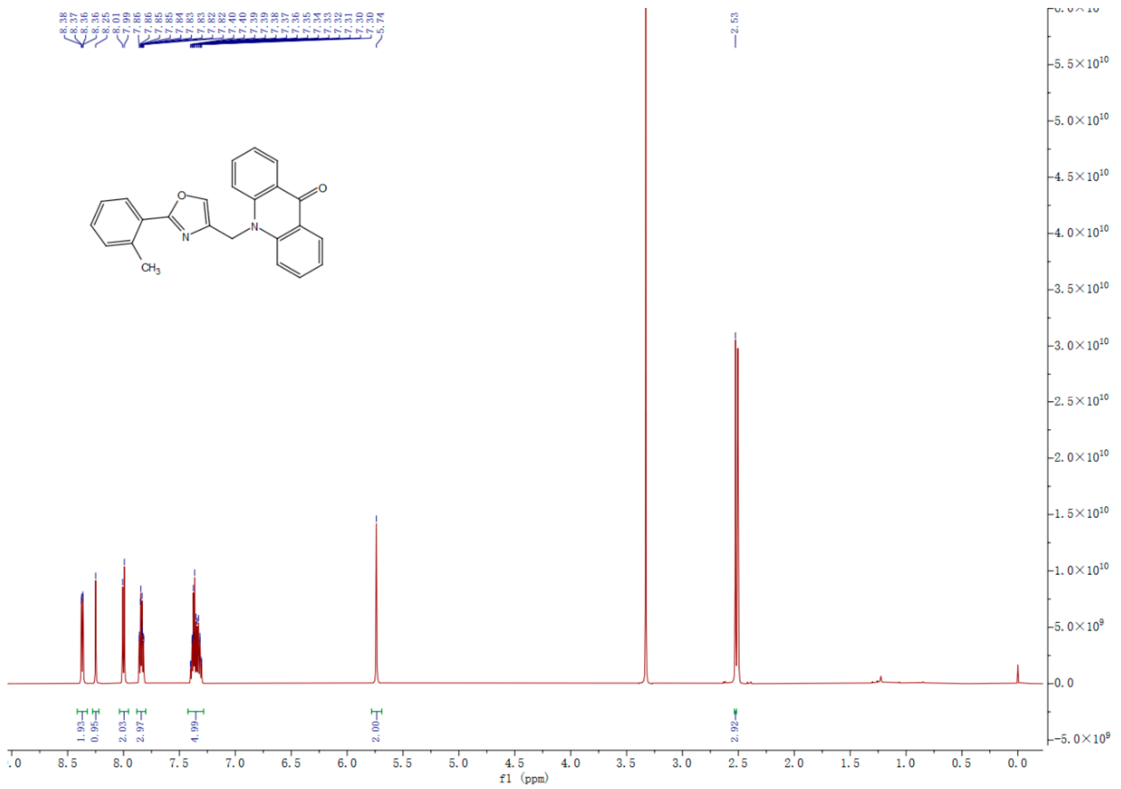

Figure S57. ^{1}H-NMR spectrum of compound **N8**.

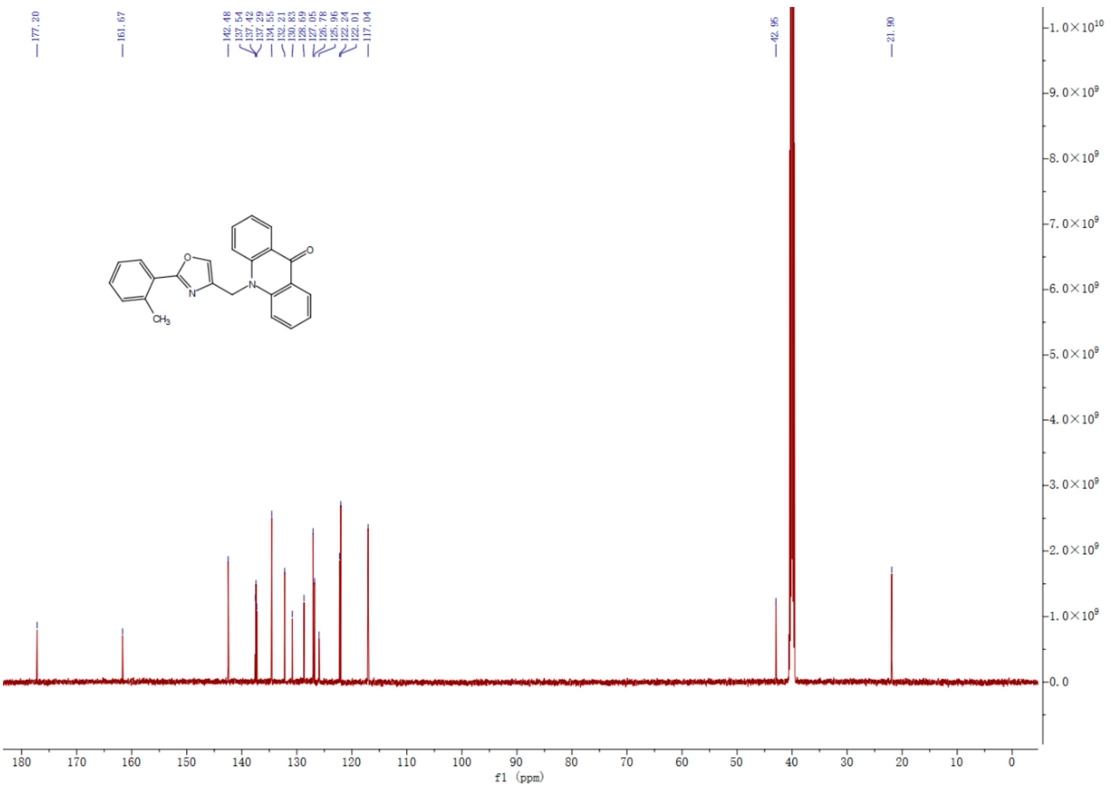

Figure S58. ^{13}C-NMR spectrum of compound **N8**.

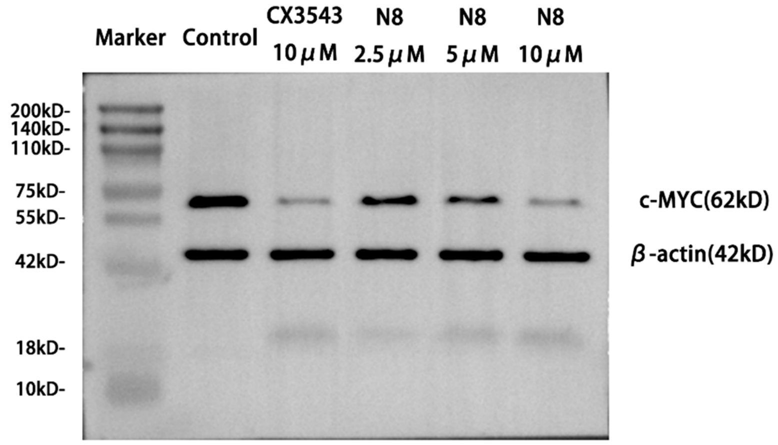

Figure S59. Full unedited gel for Figure 3i.

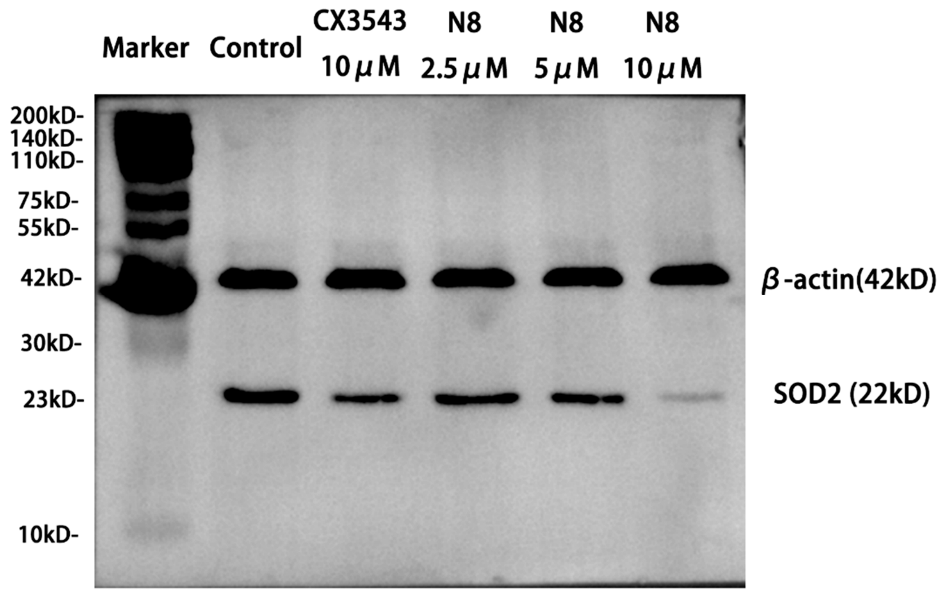

Figure S60. Full unedited gel for Figure 3k.

Table S3. Scores of molecular docking by Acridone derivatives and diverse G4 structures of different genes.

PDB_id	5w77	2m27	5zev	2o3m	7nwd	6ac7	7ys7	7x8n	6zx7	8jfq	6v0l
Gene id	c-MYC	VEGF	VEGF2	c-kit	c-kit2	PARP1	RET	k-ras	Bcl-2	EGFR	PDGFR-b
L-1	-6.56864	-5.61805	-5.85384	-5.50156	-6.65267	-6.24656	-5.20069	-5.77603	-5.73379	-6.06126	-5.8918
L2	-6.28859	-5.72593	-6.04668	-5.65163	-6.75401	-5.95605	-5.09654	-5.68876	-5.89311	-5.8363	-5.73753
L-3	-6.33087	-5.94196	-6.14747	-5.68892	-6.73451	-6.05327	-5.25033	-5.89108	-5.83088	-6.13213	-5.77398
L-4	-6.23019	-5.71335	-5.94413	-5.38709	-6.43916	-5.81193	-5.18945	-5.56877	-5.63196	-6.01703	-5.67359
L-5	-6.39616	-5.84929	-6.04147	-5.78005	-6.5284	-5.83353	-5.29293	-5.73576	-5.50746	-6.20472	-5.85581
L-6	-6.55991	-5.62845	-5.89503	-5.47913	-6.74357	-5.75274	-5.08509	-5.62864	-5.50611	-5.76787	-5.74786
L-7	-6.35974	-5.54907	-5.7017	-5.63825	-6.38781	-5.7474	-5.15686	-5.47109	-5.4203	-5.85397	-5.61363
L-8	-6.42458	-5.58457	-5.71924	-5.33689	-6.24537	-5.80804	-5.12978	-5.43065	-5.38158	-5.77677	-5.52714
L-9	-6.61806	-5.82924	-5.94788	-5.79581	-6.7114	-6.14055	-5.28216	-5.89279	-5.81771	-6.17939	-5.83432
L-10	-6.64544	-5.61632	-5.83939	-5.63784	-6.7756	-5.98292	-5.24096	-5.64569	-5.69529	-5.97239	-5.58559
L-11	-6.56743	-5.95031	-6.33679	-5.84886	-6.65279	-6.29827	-5.35458	-5.8751	-6.15573	-6.21671	-6.0853
L-12	-6.62926	-5.98916	-6.13425	-5.77286	-6.42107	-6.39762	-5.45485	-5.78476	-5.93625	-6.22678	-5.94748
L-13	-7.18691	-6.45052	-6.37711	-6.18827	-7.16834	-6.6573	-5.66273	-6.34831	-6.16344	-6.49936	-6.10042
L-14	-6.85612	-6.06415	-6.36585	-6.0617	-7.08203	-6.68473	-5.78582	-6.26337	-6.47266	-6.59458	-6.18545
L-15	-6.97908	-6.16301	-6.20792	-5.88841	-6.89278	-6.24847	-5.53395	-6.09464	-6.00032	-6.29296	-5.96325
L-16	-6.76296	-6.25766	-6.46339	-5.92241	-7.29761	-6.22346	-5.55473	-6.0027	-5.95603	-6.07334	-6.17535
L-17	-7.13871	-6.56957	-6.84043	-6.15161	-7.73619	-7.01549	-5.79902	-6.61547	-6.52292	-6.70651	-6.32013
L-18	-6.82375	-6.11282	-6.0106	-5.77095	-6.91644	-6.42452	-5.48341	-5.87786	-5.86631	-6.14264	-5.99256
L-19	-6.60542	-6.07468	-5.94774	-6.02362	-6.28388	-6.13167	-5.36817	-5.82454	-5.94762	-6.08965	-5.80258
L-20	-6.57395	-5.79684	-5.80777	-5.7754	-6.30284	-6.25647	-5.38421	-6.01585	-5.77888	-6.05605	-5.98284
L-21	-7.01649	-6.21275	-6.04636	-5.90566	-6.76149	-6.5669	-5.71287	-5.86082	-6.03615	-6.26038	-5.90004

**N-1**	-6.17299	-5.69313	-5.91756	-5.38391	-6.38423	-5.66749	-5.02482	-5.63106	-5.69194	-5.91961	-5.58913
**N-2**	-6.30186	-5.95285	-5.96547	-5.60969	-6.16972	-5.78552	-5.21037	-5.77651	-5.87241	-5.93784	-5.7039
**N-3**	-6.58843	-6.04572	-6.26519	-5.84327	-6.67691	-6.06692	-5.43532	-6.00694	-5.90471	-5.96958	-5.80198
**N-4**	-6.18447	-5.8032	-5.93745	-5.58254	-6.31122	-5.95974	-5.09082	-5.81254	-5.7035	-5.86925	-5.70411
**N-5**	-6.45512	-5.69723	-5.87228	-5.4746	-6.3102	-5.77961	-5.13383	-5.7897	-5.79297	-5.93797	-5.64619
**N-6**	-6.11453	-5.676	-6.08674	-5.57361	-6.69098	-5.86197	-5.10742	-5.80663	-5.83847	-6.03806	-5.66655
**N-7**	-6.35515	-5.9526	-6.23528	-5.75517	-6.65646	-5.80247	-5.11044	-5.92777	-5.87883	-6.11053	-5.87315
**N-8**	-6.2939	-5.56106	-5.84928	-5.43824	-6.72046	-5.83536	-5.09835	-5.78941	-5.72516	-5.95236	-5.67062
